# Semaglutide slows epigenetic aging in a randomized trial of HIV-associated lipohypertrophy

**DOI:** 10.1038/s41467-026-72861-3

**Published:** 2026-05-19

**Authors:** Michael J. Corley, Varun B. Dwaraka, Alina PS Pang, Danielle Labbato, Ryan Smith, Allison Ross Eckard, Grace A. McComsey

**Affiliations:** 1 University of California San Diego, Department of Medicine, Division of Geriatrics and Palliative Care, La Jolla, CA USA; 2 TruDiagnostic, Lexington, KY USA; 3 University Hospitals Cleveland Medical Center, Cleveland, OH USA; 4 Medical University of South Carolina, Charleston, SC USA; 5 Case Western Reserve University, Cleveland, OH USA

**Keywords:** Prognostic markers, HIV infections, Predictive markers

## Abstract

Glucagon-like peptide-1 (GLP-1) receptor agonists have attracted interest as gerotherapeutics, yet clinical-trial evidence for their effects on biological aging is lacking. We report a post hoc exploratory epigenetic age analysis of a 32-week, randomized, double-blind, placebo-controlled phase 2b trial (NCT04019197) of semaglutide in adults with human immunodeficiency virus (HIV)-associated lipohypertrophy (semaglutide *n* = 45; placebo *n* = 39). The parent trial’s primary endpoint was change in visceral adipose tissue, with secondary cardiometabolic and body-composition endpoints; epigenetic aging was not pre-specified. To address this gap, we profiled peripheral-blood DNA methylation (DNAm) at baseline and week 32 to assess semaglutide versus placebo on first-, second-, and third-generation epigenetic aging measures. In adjusted analyses, semaglutide reduced epigenetic aging across multiple second- and third-generation clocks, including PhenoAge ( − 4.9 years/year, *p* = 0.004), PCGrimAge ( − 3.1, *p* = 0.007), GrimAge V2 ( − 2.3, *p* = 0.009), OMICmAge ( − 2.2, *p* = 0.009), RetroAge ( − 2.2, *p* = 0.030), and DunedinPACE ( − 0.09 units, 9% slower, *p* = 0.01). Systems-based clocks showed parallel reductions in inflammation, brain, and heart aging measures. The post hoc design, modest sample size, HIV-specific cohort, and 32-week follow-up limit generalizability. Prospective trials are needed to determine whether GLP-1 receptor agonists can be repurposed as gerotherapeutics.

## Abstract (web summary)

Semaglutide is a glucagon-like peptide-1 (GLP-1) with a potential gerotherapeutic role. This post-hoc analysis of a phase 2b trial in adults with HIV associated lipohypertrophy reveals that semaglutide can modulate epigenetic biomarkers of aging.

## Introduction

Aging is the primary driver of chronic diseases, multimorbidity, and mortality worldwide, positioning interventions targeting biological aging as promising therapeutic strategies with potential to substantially improve human healthspan^1,2^. Within the emerging geroscience paradigm^2^, pharmacologic agents originally developed for metabolic indications, such as GLP-1 receptor agonists^3–6^, have garnered attention due to their potential dual roles in metabolic regulation and aging biology^7^. Semaglutide, a once-weekly GLP-1 receptor agonist, has emerged as an effective therapeutic for its marked weight reduction and cardiometabolic benefits, including reductions in visceral adipose tissue^3–5^. Given that obesity and adiposity embed an obesogenic epigenetic memory and are linked to accelerated epigenetic aging^8–10^, there is growing interest in whether semaglutide’s metabolic effects might slow or reverse aspects of biological aging. Despite extensive evidence supporting semaglutide’s pleiotropic benefits^11^, randomized clinical trial data evaluating its effects on aging biomarkers remain absent.

People with HIV (PWH) represent a unique population exhibiting accelerated biological aging, characterized by premature onset of age-related conditions, persistent low-grade inflammation, and metabolic dysfunction, even when HIV replication is effectively suppressed by antiretroviral therapy (ART)^12–14^. A common metabolic complication in this population is HIV-associated lipohypertrophy, defined by excessive accumulation of visceral and ectopic adipose tissue, which further exacerbates aging processes^15^. Within the geroscience framework^16^, the accelerated-aging phenotype in PWH provides an ideal clinical model to evaluate candidate geroprotective therapies, with findings potentially relevant to the general aging population. This setting may allow earlier insights into treatment effects, particularly via DNA-methylation based epigenetic clocks and other emerging aging biomarkers, which are recently being considered as outcome measures in double-blind, randomized geroscience trials^17^ and global competitions testing gerotherapeutics^18^.

In a completed phase 2b, randomized, double-blind, placebo-controlled trial^19^, we tested whether once-weekly semaglutide can slow epigenetic aging in PWH-associated lipohypertrophy, a population marked by visceral adiposity and accelerated epigenetic age. Using paired peripheral-blood methylomes collected at baseline and 32 weeks for placebo and semaglutide groups, we conducted a post hoc analysis spanning 17 first-, second-, and third-generation DNA-methylation clocks. Here, we test whether once-weekly semaglutide slows DNAm-based epigenetic age in this high-risk population. We find that a licensed GLP-1 receptor agonist modulates epigenetic biomarkers of aging, positioning semaglutide as a candidate gerotherapeutic and laying the groundwork for prospectively powered, mechanism-focused trials aimed at extending healthspan in populations vulnerable to accelerated aging.

## Results

### Post hoc epigenetic age analysis of a randomized trial of semaglutide

**Fig. 1:**
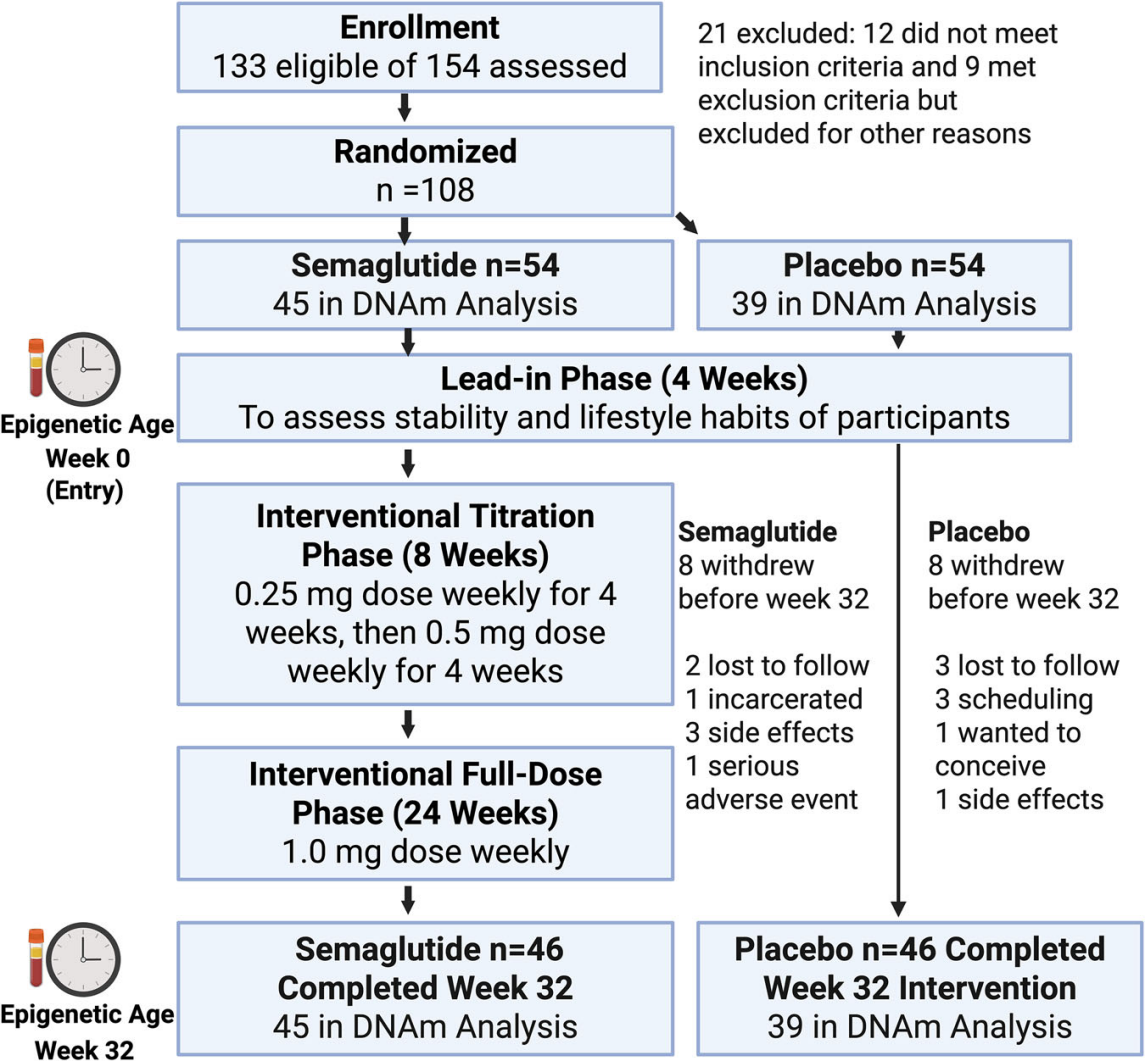
Consort diagram for the semaglutide trial in PWH. Epigenetic age was assayed from peripheral blood mononuclear cell samples collected at baseline (Week 0) and the post-interventional phase follow-up time point (Week 32). Of the total 92 participants that completed longitudinal Week 0 and Week 32 assessments, epigenetic age was assayed longitudinally for *n* = 45 semaglutide and *n* = 39 placebo participants. Created in BioRender. Corley, M. (2026). https://BioRender.com/sk6x1pm.

To determine whether semaglutide treatment could impact biological aging, we conducted a post hoc epigenetic analysis of participants enrolled in a previously reported 32-week, randomized, double-blind, placebo-controlled phase 2b clinical trial (NCT04019197) evaluating semaglutide in PWH and lipohypertrophy^19^ (Fig. 1). The trial aimed to evaluate the effects of the GLP-1 receptor agonist semaglutide on adipose tissue quantity and distribution in individuals with HIV-associated lipohypertrophy over a 32-week period. Eligible participants included adults aged 18 years or older with documented HIV-1 infection, stable ART for at least 12 weeks, and controlled HIV-1 RNA levels (< 400 copies per mL) for six months prior to screening. Additional inclusion criteria were a body mass index (BMI) of 25 kg/m² or greater and the presence of lipohypertrophy without type 1 or type 2 diabetes or cardiovascular disease. Participants were randomly assigned 1:1 to receive either once-weekly subcutaneous semaglutide (8-week dose titration followed by 24 weeks at 1.0 mg) or matching placebo. All weekly injections were provided in the clinic by a certified nurse. Randomization was performed using an online software program with block sizes of six, and treatment assignments were masked to participants, investigators, and research personnel. Of 154 individuals assessed for eligibility, 108 were randomized (54 in each group). Eight participants (15%) from each group withdrew prematurely. The study’s primary outcomes included changes in adipose tissue quantity across body compartments; abdominal visceral and subcutaneous adipose tissue were measured by L4-L5 non-contrast abdominal computed tomography (CT) scan imaging, while total body fat, trunk and peripheral fat were measured by whole body dual-energy X-ray absorptiometry scan. Secondary outcomes included metabolic measures (glucose metabolism, insulin resistance, lipid profiles), anthropometric changes (weight, BMI, waist-to-hip ratio), inflammation and immune activation markers, and safety. Results for the primary and secondary outcomes were published^19^. The trial is described in more detail at ClinicalTrials.gov (NCT04019197) and in “Methods”. Peripheral blood mononuclear cells (PBMCs) were collected at baseline and 32 weeks follow-up and biobanked.

We first quantified biological age using established first^20–23^, second^24–26^, and third- generation epigenetic clocks^27–29^ in biobanked longitudinal peripheral-blood mononuclear cells from 84 total participants (45 semaglutide, 39 placebo) using epigenetic DNA-methylation data obtained at baseline and week 32. We also utilized versions of first- and second-generation clocks built from DNAm principal components (PCs) (termed PC clocks) that show enhanced technical reliability and utility in longitudinal study designs such as randomized clinical trials^30^. First-generation clocks included Horvath’s original clock^20^, Horvath’s Skin & Blood clock^22^, Hannum^21^, PCHorvath1^30^, PCHorvath2^30^, and PCHannum^30^; second-generation clocks included PhenoAge^24^, PCPhenoAge^30^, PCGrimAge^25,30^, and DNAmFitAge^26^; and third-generation clocks included OMICmAge^31^, RetroAge^23^, SystemsAge^29^, AdaptAge^28^, CausAge^28^, and DamAge^28^. We additionally assessed pace-of-aging using DunedinPACE^27^ and IntrinsicCapacity^32^. At baseline, participants’ chronological age was highly correlated with epigenetic age estimates across epigenetic clocks (Pearson *r* = 0.83–0.99) (Supplementary Fig. 1).

For the epigenetic analysis, at baseline, the 84 participants (45 semaglutide, 39 placebo) were middle-aged, with a mean ± standard deviation (SD) age of 49 ± 12 years and well balanced between treatment arms (48 ± 13 vs. 50 ± 12 years). 42% were women overall, but men were slightly over-represented in the semaglutide group (67% vs. 49%). The cohort included 58% Black, 38% White and 11% Hispanic participants with nearly identical distributions across groups. Immunologically, CD4 counts were high (median 762 cells µL^−1^) and CD4/CD8 ratios near 1.0, reflecting immune reconstitution; nadir CD4 counts were lower, as expected, but similar between arms. Viral suppression was durable: only 9 % had HIV-1 RNA above the lower limit of quantification, and ART duration averaged ~14 years. Participants were obese (median BMI 32.9 kg m⁻²) with comparable anthropometry in each group. One-third were current smokers, another quarter former smokers. Glycemic control was normal (median glycated hemoglobin (HbA1c) 5.5%), although insulin resistance was evident (median fasting homeostatic model assessment of insulin resistance (HOMA-IR) 2.9), again without meaningful group differences. Estimated 10-year atherosclerotic cardiovascular disease risk was moderate at 4.7% (interquartile range 2.2–8.0). Inflammatory biomarkers showed low-grade activation: median high-sensitivity C-reactive protein (hsCRP) 4.1 µg/mL and soluble CD163 (sCD163) 605 pg mL⁻¹, with slightly higher values in placebo. Overall, baseline characteristics of participants assayed in the epigenetic analysis were well matched (Table 1).

**Table 1:** Baseline characteristics of the clinical trial population overall and by treatment group

Baseline characteristic		All participants (*N* = 84)	Semaglutide (*n* = 45)	Placebo (*n* = 39)
Age (years)	Mean (SD)	49 (12.5)	48 (13.1)	50 (11.8)
Sex	Male	49 (58%)	30 (67%)	19 (49%)
	Female	35 (42%)	15 (33%)	20 (51%)
Race or ethnicity	Black	49 (58%)	26 (58%)	23 (59%)
	White	32 (38%)	18 (40%)	14 (36%)
	Biracial	2 (2%)	0	2 (5%)
	Native American	1 (1%)	1 (2%)	0
	Hispanic	9 (11%)	4 (9%)	5 (13%)
HIV variables	CD4 T-cell count, cells per µl	762 (525, 1058)	830 (389.3, 1081)	728 (547, 923)
	CD8 T-cell count, cells per µl	806 (571, 1054)	762.5 (572, 1074)	847 (571, 1023)
	CD4/CD8 ratio	0.98 (0.61, 1.43)	1.04 (0.51, 1.31)	0.97 (0.61, 1.43)
	Nadir CD4, cells per µl	211 (97, 377)	266 (105.5, 509)	163 (62.25, 313)
	HIV-1 RNA (copies/mL) > LLQ	8 (9%)	3 (7%)	5 (13%)
	HIV duration, months	228.8 (139.7, 312.6)	200 (113.4, 491.2)	235.8 (158.4, 301.7)
	Antiretroviral therapy duration, months	175.1 (107.5, 223.8)	184.6 (97.49, 223.2)	160.5 (109.9, 225.6)
Anthropometric measurements	BMI, kg/m^2^	32.86 (28.82, 38.97)	32.80 (28.61, 35.93)	33.09 (28.99, 39.73)
Lifestyle variables	Current smoker	29 (35%)	13 (29%)	16 (41%)
	Past smoker	21 (25%)	12 (27%)	9 (23%)
	Never smoked	34 (40%)	20 (44%)	14 (36%)
Glucose metabolism and insulin resistance	HbA1c %	5.5 (5.2, 5.8)	5.4 (5.1, 5.8)	5.6 (5.4, 5.9)
	Fasting HOMA-IR	2.9 (2.0, 5.1)	2.6 (1.4, 4.6)	3.7 (2.2, 7.5)
	2-h OGTT HOMA-IR	10.3 (5.1, 26.8)	9.9 (4.9, 19.1)	10.4 (5.1, 42.0)
Cardiovascular disease risk	10-year atherosclerotic cardiovascular disease risk estimate, %	4.7 (2.2, 8.0)	4.5 (2.4, 7.7)	4.8 (1.9, 8.8)
Plasma biomarkers	hs-CRP (µg/mL)	4.142 (2.021, 7.976)	3.785 (1.743, 6829)	5.713 (2.663, 9.668)
	sCD163 (pg/L)	604.7 (456.4, 849.1)	591.9 (458.1, 752.8)	616.9 (446.9, 993.7)
	sCD14 (pg/L)	1785 (1505, 2122)	1683 (1398, 2085)	1927 (1650, 2130)

### Semaglutide slows epigenetic aging across multiple DNA methylation clocks

**Fig. 2:**
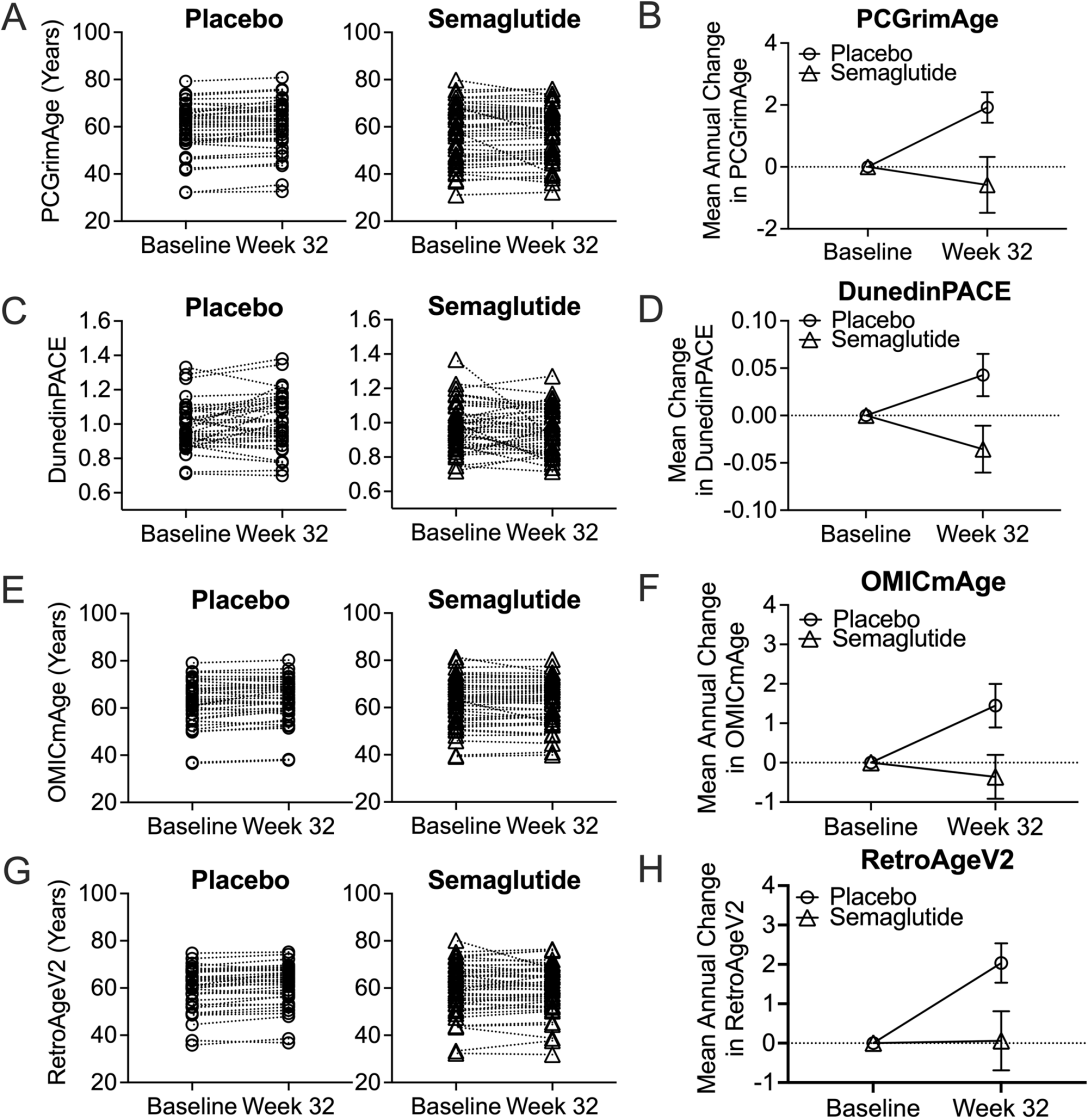
Change in epigenetic age from baseline to week 32 follow-up in PCGrimAge, DunedinPACE, OMICmAge, and RetroAge in the placebo and semaglutide groups. **A** PCGrimAge individual participant trajectories and **B** mean annual change from baseline to Week 32. **C** DunedinPACE individual participant trajectories and **D** mean change. **E** OMICmAge individual participant trajectories and **F** mean annual change. **G** RetroAgeV2 individual participant trajectories and **H** mean annual change. Calculated values are displayed in open circles for each participant in the placebo and open triangles for semaglutide group. *n* = 39 placebo and *n* = 45 semaglutide biologically independent participants, each representing a biologically independent participant enrolled in the trial with paired baseline and week 32 epigenetic age estimates. Individual participant-level data are presented as mean values ± SEM. Between-group differences in annual change from baseline to Week 32 were assessed using unpaired two-tailed Welch’s *t*-tests. The semaglutide group showed significantly less epigenetic aging than placebo on all four clocks: PCGrimAge (*t*(67.1) = 2.43, *P* = 0.018; mean difference −2.50 years, 95% CI: −4.55 to −0.45), DunedinPACE (*t*(81.9) = 2.35, *P* = 0.021; mean difference −0.078 pace-of-aging units, 95% CI: −0.145 to −0.012), OMICmAge (*t*(81.6) = 2.31, *P* = 0.024; mean difference −1.81 years, 95% CI: −3.36 to −0.25), and RetroAgeV2 (*t*(74.6) = 2.20, *P* = 0.031; mean difference −1.98 years, 95% CI: −3.77 to −0.18). *P*-values are two-sided and were not adjusted for multiple comparisons. Source data are provided as a Source Data file.

**Fig. 3:**
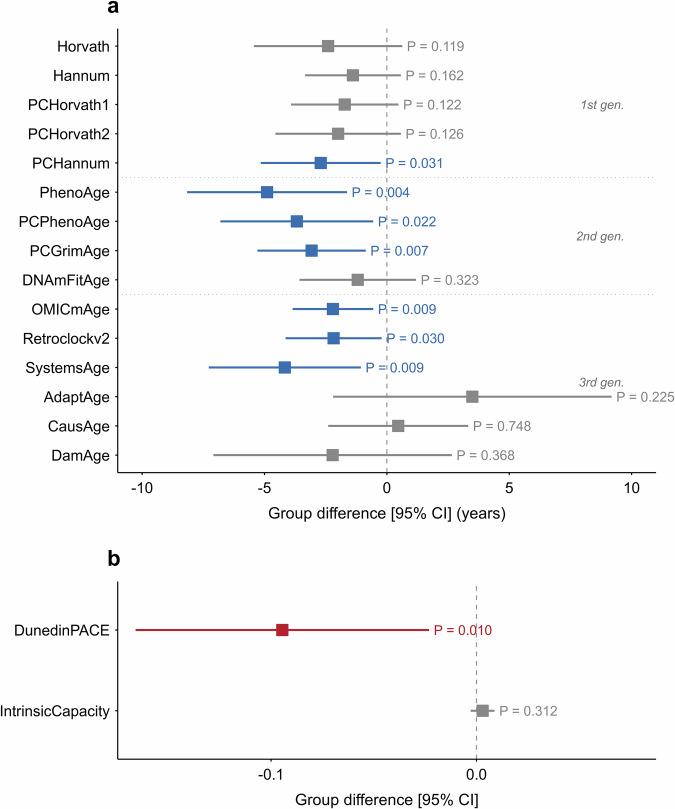
Semaglutide broadly decelerates epigenetic aging across multiple methylation clocks. Forest plot of adjusted treatment effects (semaglutide minus placebo) on annualized epigenetic age change for DNA methylation–based biomarkers of aging. Point estimates (filled squares) represent the adjusted mean difference derived from the randomization coefficient in ANCOVA models adjusting for sex, baseline BMI, and inflammation (hsCRP and sCD163). Horizontal lines indicate 95% confidence intervals. The dashed vertical line at zero represents no between-group difference. **a** Effects on 15 epigenetic clocks spanning first-generation (Horvath, Hannum), principal-component-based (PCHorvath1/2, PCHannum, PCPhenoAge), and second- and third-generation (PhenoAge, PCGrimAge, AdaptAge, CausAge, DamAge, DNAmFitAge, OMICmAge, RetroAge (Retroclockv2), SystemsAge). **b** DunedinPACE, a continuous pace-of-aging measure (units of years aged per chronological year), and Intrinsic Capacity epigenetic clock (IC score). Negative estimates indicate slower biological aging in the semaglutide group. In (**a**), blue-filled squares and horizontal lines denote statistically significant results (*p* < 0.05) and grey denotes non-significant results (*p* ≥ 0.05). In (**b**), red-filled squares and horizontal lines denote statistically significant results and grey denotes non-significant results. Blue and red distinguish clocks measured in epigenetic age units (years) from those measured on alternative scales (pace-of-aging units for DunedinPACE, IC score units for Intrinsic Capacity). *n* = 39 placebo and *n* = 45 semaglutide biologically independent participants, each representing a unique individual enrolled in the trial with paired baseline and Week 32 epigenetic age estimates. *P*-values are two-sided, unadjusted for multiple comparisons. Source data are provided as a Source Data file.

Semaglutide treatment resulted in significantly slower epigenetic aging compared with placebo, evidenced by stable or reduced age acceleration across multiple DNAm epigenetic clocks (PCGrimAge, DunedinPace, OMICmAge^31^, and RetroAge) while placebo showed consistent increases over 32 weeks (Fig. 2A–H). To formally test these differences and adjust for baseline clinical and inflammatory factors, we evaluated the impact of semaglutide treatment on biological aging using 17 DNAm-based epigenetic clocks spanning first-, second-, and third-generation epigenetic clocks. All epigenetic age estimates were annualized to reflect the projected rate of change over one calendar year (years/year) from the 32-week treatment period. In analysis of covariance (ANCOVA) models adjusting for baseline covariates (sex, BMI, plasma hsCRP, and plasma sCD163), semaglutide was associated with significantly reduced epigenetic aging relative to placebo across multiple generations of epigenetic clocks (Fig. 3a). The most pronounced effects were observed for second-, and third-generation epigenetic clocks. Among second-generation clocks, PhenoAge showed the largest reduction (−4.90 years/year [95% CI: −8.16 to −1.63], *P* = 0.004), followed by PCPhenoAge (−3.68 years/year [−6.80 to −0.56], *P* = 0.022) and PCGrimAge (−3.08 years/year [−5.29 to −0.86], *P* = 0.007). DNAmFitAge did not reach statistical significance (−1.19 years/year [−3.57 to 1.19], *P* = 0.323). For DunedinPACE, semaglutide was associated with a 0.09 lower pace of aging (units per year) relative to placebo (95% CI: −0.17 to −0.02, *P* = 0.010) (Fig. 3b), translating to an approximately 9% slower pace of biological aging in the semaglutide group. Among third-generation clocks, SystemsAge showed the largest effect (−4.17 years/year [95% CI: −7.28 to −1.06], *P* = 0.009), followed by OMICmAge (−2.20 years/year [−3.85 to −0.56], *P* = 0.009), a next-generation DNAm clock derived by modelling an electronic medical record–based aging phenotype with epigenetic, proteomic, metabolomic and clinical biomarker proxies^31^. RetroAge, an epigenetic clock designed to capture retrotransposon-associated aging^23^, also showed a significant response to semaglutide treatment (−2.18 years/year [−4.14 to −0.21], *P* = 0.030) (Fig. 3a). In contrast, causality-enriched epigenetic clocks^28^ AdaptAge, CausAge, and DamAge exhibited directionally heterogeneous and statistically non-significant changes: AdaptAge increased by 3.49 years/year ([−2.20 to 9.18], *P* = 0.225), CausAge by 0.46 years/year ([−2.40 to 3.32], *P* = 0.748), and DamAge decreased by 2.22 years/year ([−7.09 to 2.65], *P* = 0.368), highlighting the differential responsiveness of epigenetic clocks to semaglutide treatment. Among first-generation epigenetic clocks, only PCHannum showed a significant reduction in epigenetic age (−2.70 years/year [95% CI: −5.15 to −0.25], *P* = 0.031), whereas Horvath (−2.40 years/year [−5.44 to 0.63], *P* = 0.119), Hannum (−1.39 years/year [−3.34 to 0.57], *P* = 0.162), PCHorvath1 (−1.72 years/year [−3.92 to 0.47], *P* = 0.122), and PCHorvath2 (−1.99 years/year [−4.55 to 0.57], *P* = 0.126) showed directionally consistent but nominally non-significant reductions (Fig. 3a). Unadjusted results are shown in Supplementary Fig. 2. Importantly, DNA methylation–based immune cell-type deconvolution analysis revealed no significant differences in estimated proportions of major peripheral immune cell populations between treatment groups over the 32 weeks (Supplementary Fig. 3), suggesting that the observed epigenetic age reductions were not confounded by shifts in blood cell composition.

### Intrinsic capacity (IC) clock shows no significant change with semaglutide treatment

A DNAm-based epigenetic clock of IC was recently developed using elastic net regression in the INSPIRE-T cohort, identifying 91 CpG sites predictive of IC scores derived from clinical assessments^32^. This IC epigenetic clock showed strong age-related decline and only modest overlap with traditional epigenetic clocks, suggesting it captures a distinct axis of biological aging linked to physical and cognitive resilience. Hence, we evaluated the responsiveness of this first-generation IC clock to semaglutide in our study. In adjusted ANCOVA models controlling for age, sex, BMI, hsCRP, and sCD163, semaglutide treatment did not significantly alter IC clock estimates relative to placebo over the 32-week period (0.003 IC units [95% CI: −0.003 to 0.009], *P* = 0.312) (Fig. 3b). The responsiveness of this first-generation IC epigenetic clock to semaglutide remains uncertain and warrants further investigation in larger, longitudinal cohorts.

### Semaglutide reduction in epigenetic age as measured by GrimAge and GrimAgeV2 using Biolearn

**Fig. 4:**
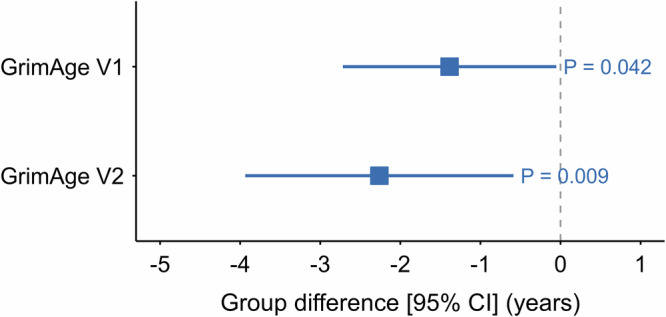
Effect of semaglutide on GrimAge V1 and V2 epigenetic aging measures calculated using the Biolearn platform. Forest plot of adjusted treatment effects (semaglutide minus placebo) on annualized epigenetic age change for the GrimAge V1 and V2 DNA methylation clocks computed using the Biolearn framework. GrimAge V1 and V2 refer to versions 1 and 2 of the DNA methylation-based GrimAge epigenetic age mortality-risk clocks. Point estimates (filled squares) represent the adjusted mean difference. Horizontal lines indicate 95% confidence intervals. The dashed vertical line at zero represents no between-group difference. Negative values indicate slower epigenetic aging in the semaglutide group. Blue-filled squares and horizontal lines denote statistically significant results (*p* < 0.05). Between-group differences were estimated using ANCOVA, adjusting for sex, baseline body mass index (BMI), high-sensitivity C-reactive protein (hsCRP), and soluble CD163 (sCD163). *n* = 39 placebo and *n* = 45 semaglutide biologically independent participants, each with paired baseline and Week 32 peripheral-blood mononuclear cell (PBMC) samples. *P*-values are two-sided, unadjusted for multiple comparisons. Source data are provided as a Source Data file.

As an orthogonal computational approach to assess biological aging with second-generation mortality-based epigenetic clocks, we used Biolearn^33^, an open-source library for biomarkers of aging, to examine whether semaglutide treatment significantly impacted DNAm-based age estimates for GrimAge V1^25^ and the updated GrimAge V2^34^, which extends V1 by incorporating additional DNAm surrogates for hemoglobin A1c and C-reactive protein. Using adjusted ANCOVA models controlling for baseline sex, BMI, hsCRP, and sCD163, semaglutide was associated with significant reductions in both GrimAge V1 and GrimAge V2 (Fig. 4). Participants randomized to semaglutide exhibited a 1.39 years/year lower GrimAge V1 estimate compared to placebo (95% CI: −2.72 to −0.05, *P* = 0.042), with a more pronounced effect for GrimAge V2 (−2.26 years/year [−3.94 to −0.59], *P* = 0.009) (Fig. 4), consistent with the enhanced sensitivity of V2 to cardiometabolic and inflammatory components. These findings, derived using an independent modelling framework, provide orthogonal confirmation that semaglutide may attenuate biological aging as captured by mortality risk–associated epigenetic signatures.

### Semaglutide broadly decelerates multi-system epigenetic aging across 11 system clocks

**Fig. 5:**
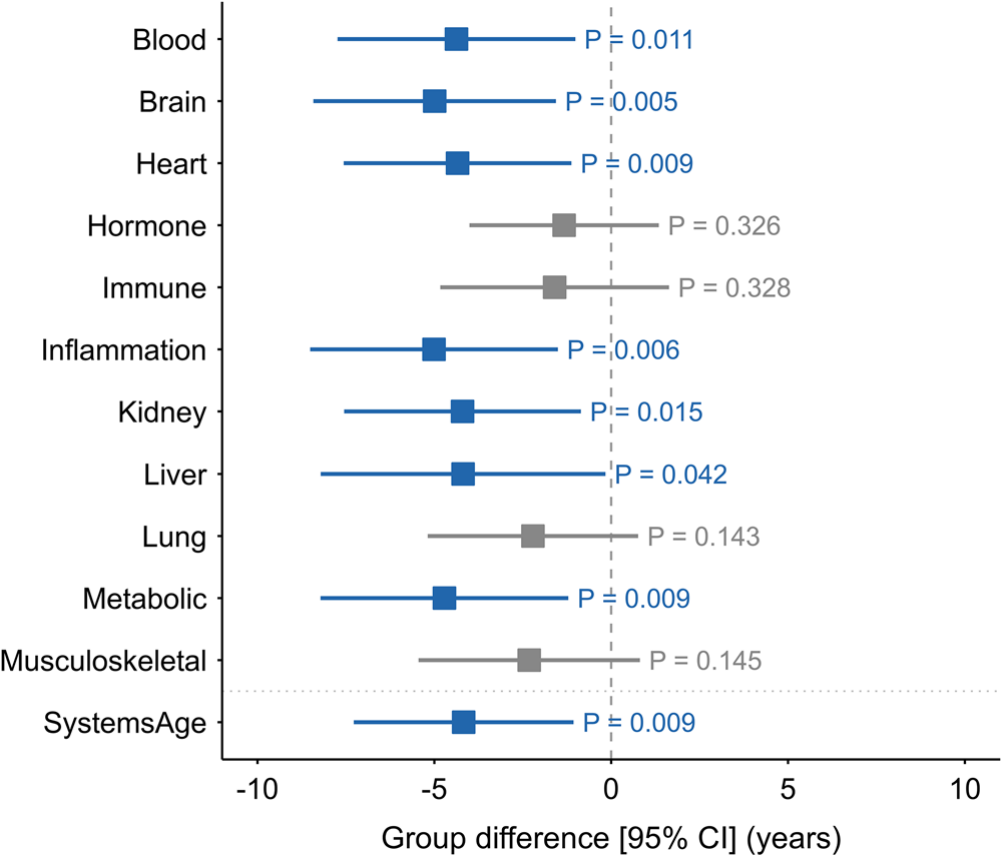
Semaglutide confers a multi-organ deceleration of system-level epigenetic aging. Forest plot of the between-group difference (semaglutide minus placebo) in annualized epigenetic age (years per year) across 11 organ-system DNAm clocks and a composite SystemsAge index. Point estimates (filled squares) represent the adjusted mean difference derived from the randomization coefficient in ANCOVA models controlling for baseline BMI, hsCRP, sCD163, and sex. Horizontal lines indicate 95% confidence intervals. The dashed vertical line at zero represents no between-group difference. Negative values indicate slower system-specific epigenetic aging in the semaglutide group. System-specific clocks were derived from a single blood methylation assay that deconvolves biological aging across distinct physiological systems. *n* = 39 placebo and *n* = 45 semaglutide biologically independent participants, each representing a unique individual enrolled in the trial with paired baseline and Week 32 epigenetic age estimates. No adjustment for multiple comparisons was applied, as these epigenetic aging analyses were exploratory and post hoc. Source data are provided as a Source Data file.

Because GLP-1 receptor agonists like semaglutide have demonstrated pleiotropic benefits that span cardiometabolic, renal, hepatic and neuroprotective domains^11,35^, we next evaluated whether these effects extended to biological aging across distinct physiological systems. We applied a panel of 11 DNAm-based systems epigenetic clocks derived from a single blood methylation assay that deconvolves biological aging across 11 distinct physiological systems, including the Heart, Lung, Kidney, Liver, Brain, Immune, Inflammatory, Blood, Musculoskeletal, Hormone and Metabolic^29^. Each system clock captures both all-cause mortality risk and systems-specific decline; for example, the Heart clock predicts cardiovascular events, while the Brain clock tracks cognitive function and neuroimaging correlates. In adjusted ANCOVA models controlling for age, sex, BMI, hsCRP, and sCD163, semaglutide treatment was associated with consistent reductions in epigenetic age across all 7 systems and the composite Systems Age clock (Fig. 5). The largest effects were observed in the Inflammation (−5.01 years [95% CI: −8.51 to −1.51], *p* = 0.006), Brain (−4.99 years [−8.42 to −1.56], *p* = 0.005), and Metabolic (−4.72 years [−8.22 to −1.21], *p* = 0.009) clocks. Substantial reductions were also observed in the Blood (−4.37 years [−7.74 to −1.01], *p* = 0.011), Heart (−4.34 years [−7.56 to −1.12], *p* = 0.009), Kidney (−4.20 years [−7.55 to −0.86], *p* = 0.015), and Liver (−4.19 years [−8.21 to −0.16], *p* = 0.042) clocks. Reductions in epigenetic age were also observed in the Lung (−2.21 years [−5.19 to 0.76], *p* = 0.14), Musculoskeletal (−2.32 years [−5.45 to 0.81], *p* = 0.15), Immune (−1.60 years [−4.83 to 1.64], *p* = 0.33), and Hormone (−1.33 years [−4.01 to 1.35], *p* = 0.33) system clocks, though none reached statistical significance. These findings highlight the potential of semaglutide to exert geroprotective effects that extend across multiple physiological systems.

## Discussion

This post hoc analysis study provides randomized clinical trial evidence that once-weekly semaglutide can modulate biological aging in a high-risk population, as measured by DNAm-based epigenetic clocks. Over the 32-week intervention period in individuals with HIV-associated lipohypertrophy, a population characterized by metabolic dysfunction and accelerated biological aging, semaglutide treatment led to attenuation of biological age-related DNAm signatures based on epigenetic clock estimates. Notably, the most pronounced effects were observed in second-generation clocks predictive of morbidity and mortality risk and in third-generation measures of biological aging rate (DunedinPACE) suggesting that semaglutide may exert pleiotropic effects that extend beyond metabolic regulation to influence biological aging.

Our results align well with emerging geroscience paradigms, which propose that lifestyle and metabolic interventions capable of reducing adiposity, systemic inflammation, and insulin resistance could significantly modulate aging trajectories and improve healthspan^2^. Notably, similar epigenetic clock signals have been observed with caloric restriction (Comprehensive Assessment of Long-term Effects of Reducing Intake of Energy (CALERIE) trial^36^) and multimodal lifestyle interventions (DO-HEALTH trial^37^), supporting the broader concept that targeted metabolic modulation can impact aging pathways. Given the considerable burden of multimorbidity and mortality driven by age-related chronic conditions worldwide, identifying pharmacological agents like semaglutide capable of slowing biological aging represents an advancement for geroscience^17^. Further longitudinal studies specifically examining the effects of GLP-1 receptor agonists on validated biomarkers of aging are warranted to clarify their role within the expanding geroscience therapeutic landscape.

Semaglutide’s multi-organ impact^3–5,38^, observed consistently across system-specific epigenetic clocks, further underscores its gerotherapeutic potential. Strong reductions in biological age within inflammation, brain, cardiovascular, hepatic, and renal systems suggest pleiotropic geroprotective mechanisms consistent with emerging literature indicating broad systemic benefits of GLP-1 receptor agonists. Indeed, we observed previously that semaglutide treatment markedly decreased inflammation-associated biomarkers (interleukin-6 and sCD163) previously linked to morbidity and mortality in HIV^39^. These findings align with emerging mechanistic studies in murine models showing that GLP-1 receptor agonists can reverse obesogenic memory through anti-inflammatory and metabolic reprogramming of adipose tissue, targeting pathways such as C–C motif chemokine ligand 2 (CCL2)/C–C chemokine receptor type 2 (CCR2) that drive epigenetic and metabolic dysfunction^40^. Recent proteomic studies have also revealed that semaglutide alters circulating proteins involved in lipid metabolism, inflammation, and cardiovascular risk pathways independent of weight loss, suggesting broader reprogramming effects that could synergize with epigenetic aging deceleration^6^. These data suggest a plausible mechanistic link wherein semaglutide mitigates chronic immune activation and inflammatory signaling pathways central to aging acceleration, particularly relevant in populations experiencing chronic inflammation such as PWH.

Our results suggest that semaglutide’s geroprotective effects on epigenetic clocks may stem from its potent reduction of visceral adipose tissue, which appears to drive epigenetic age acceleration more strongly than systemic inflammation in our cohort. Recent studies have demonstrated that adipose tissue retains a persistent obesogenic epigenetic memory, characterized by stable transcriptional and chromatin accessibility changes even after significant weight loss, predisposing individuals to adverse metabolic responses upon weight regain^10^. Improved adipose tissue function and enhanced insulin sensitivity achieved through semaglutide treatment could thus disrupt or partially reverse stochastic epigenetic signatures that govern the aging process^41^ and associations with disease outcomes^42^. Further mechanistic investigations into adipocyte-specific methylation patterns, chromatin remodeling, and transcriptional responses to semaglutide will be critical to confirm its role in counteracting adipose-derived epigenetic memory and reducing susceptibility to metabolic dysfunction and accelerated biological aging.

Our study also highlights important methodological insights regarding epigenetic clocks as outcome measures^43–45^. We observed heterogeneous responsiveness across different classes of epigenetic biomarkers. While second-generation mortality-linked clocks, pace of aging, and multi-system indices demonstrated significant treatment effects, clocks designed to capture resilience (AdaptAge, IC clock) or causally-driven aging (CausAge, DamAge) were less responsive. This finding emphasizes the need for careful biomarker selection in geroscience trials, acknowledging unique epigenetic clock-specific attributes. Moreover, despite these promising findings, several limitations of our findings should be considered. This study represents a post hoc analysis, limited by sample size and short follow-up duration. Longer-term trials with larger cohorts are necessary to validate durability, translate epigenetic changes into clinical outcomes, and assess generalizability beyond the unique HIV-associated lipohypertrophy population. Additionally, exploring alternative GLP-1 agonists, dosing or treatment regimens (e.g., microdosing) could enhance applicability to broader populations interested in preventive aging interventions.

ART history represents an important consideration in interpreting these findings, as specific ART classes and cumulative exposure, particularly earlier thymidine analogs and certain protease inhibitors, have been linked to lipohypertrophy, metabolic dysfunction, inflammation, and epigenetic alterations in PWH. Although our analysis did not explicitly stratify by detailed ART exposure, participants were randomized and maintained on stable ART regimens prior to enrollment, reducing the likelihood of systematic imbalance between treatment arms; thus, observed differences in epigenetic aging trajectories are unlikely to be driven solely by ART effects. Nonetheless, heterogeneity in prior ART exposure, duration of HIV infection, and cumulative metabolic injury may have influenced baseline epigenetic age or modified responsiveness to semaglutide. Notably, the attenuation of epigenetic age acceleration across multiple clocks despite lifelong ART exposure suggests that GLP-1 receptor agonism may act downstream of HIV- and ART-related changes, counteracting shared metabolic and inflammatory aging pathways. This distinction is particularly relevant given growing interest in repurposing reverse transcriptase inhibitors as gerotherapeutics to suppress age-associated transposable element reactivation, a process increasingly implicated in genomic instability, inflammation, and epigenetic aging. Future studies incorporating ART exposure switches will be critical for disentangling ART-related versus adiposity-driven mechanisms of aging and for optimizing geroscience-guided interventions in this population.

Our study has several important limitations that warrant consideration. First, DNAm was measured in PBMCs, and although we applied validated DNAm-based cell deconvolution methods to estimate leukocyte subtypes, residual confounding due to subtle shifts in immune cell composition cannot be fully excluded. Relatedly, organ- and system-specific epigenetic clocks were derived from blood-based methylation profiles and therefore represent indirect, integrative biomarkers of organ-system aging rather than direct measures of tissue-resident epigenetic change. As such, these clocks should not be interpreted as definitive evidence of tissue-level aging or rejuvenation, and future studies incorporating biopsy-based methylation profiling of adipose, muscle, or other relevant tissues will be important for validation. Second, while semaglutide was studied within a randomized controlled trial, our epigenetic analyses did not explicitly stratify by detailed ART history, which may independently influence adiposity, metabolic health, immune function, and epigenetic aging in PWH. Although prior analyses of the parent trial suggest minimal impact of ART regimen on clinical outcomes, ART-specific epigenetic effects remain an important area for future investigation in larger cohorts. Third, epigenetic clocks are statistical predictors rather than mechanistic measures of aging, and changes in clock-derived age acceleration should not be interpreted as direct evidence of altered lifespan or causative aging processes. Instead, these measures likely reflect downstream or correlative methylation changes at CpG sites sensitive to metabolic, inflammatory, or treatment-related perturbations. In addition, the biological significance of small methylation differences detected by high-density arrays, such as the Illumina EPICV2 platform, remains uncertain at individual CpG resolution, even when aggregated into validated clock metrics. Finally, despite the longitudinal trial design, serial biospecimens following the trial were not examined, precluding assessment of longer-term epigenetic aging trajectories. Replication in larger, more diverse cohorts with extended follow-up, complementary geroscience biomarkers, and direct tissue profiling will be essential to further establish the clinical relevance and mechanistic basis of semaglutide-associated changes in epigenetic aging biomarkers.

These findings support semaglutide’s potential as a gerotherapeutic capable of influencing fundamental aging processes. By demonstrating semaglutide’s significant impact across multiple validated epigenetic aging clocks, our results provide evidence for systematically evaluating GLP-1 receptor agonists through Food and Drug Administration-approved drug repurposing frameworks, accelerating their clinical translation as geroscience-guided therapies to enhance healthspan and prevent chronic age-related diseases.

## Methods

### Ethical approval

The current work complies with the ethical principles of the Declaration of Helsinki. This study used datasets from Dr. McComsey’s biobank (project grant R01DK121619) approved by the University Hospitals Cleveland Medical Center Committee. The Institutional Review Board (IORG000040) approved the study. Participants received the study drug free of charge. Participants received financial compensation for time and travel: $25 for interim visits and $50–$75 for comprehensive study visits. Parking was provided for all study visits. All participants signed a written informed consent form.

### Study design and participants

This post hoc exploratory epigenetic analysis leveraged a completed clinical trial. The Once-Weekly Semaglutide in PWH-Associated Lipohypertrophy study was a single-center, randomized, double-blind, placebo-controlled phase 2b trial (ClinicalTrials.gov NCT04019197) conducted at University Hospitals Cleveland Medical Center (Cleveland, OH). The primary trial objective was to evaluate semaglutide’s effects on body fat distribution in PWH over 32 weeks. Key inclusion criteria were: age ≥18 years, documented HIV-1 infection on stable ART for ≥12 weeks, HIV-1 RNA < 400 copies/mL for ≥6 months, BMI ≥ 25 kg/m², and clinical evidence of lipohypertrophy (central fat accumulation) with waist circumference >95 cm (men) or >94 cm (women) and waist-to-hip ratio >0.94 (men) or >0.88 (women). Participants with diabetes or active cardiovascular disease were excluded, as were those pregnant or with uncontrolled comorbidities. A total of 154 individuals were screened, 108 were randomized (54 to each group). Eight participants (15%) in each arm discontinued prematurely, leaving 92 who completed 32 weeks; of these, 84 had paired samples available for epigenetic analysis. Randomization was 1:1 to semaglutide or placebo, stratified by sex, using block sizes of six via an online randomization system. Both participants and investigators were blinded to treatment assignment. Semaglutide was administered by experienced clinic nurses subcutaneously once weekly, with dose escalation (0.25 mg for 4 weeks, to 0.5 mg for 4 weeks, then to 1.0 mg), then 1.0 mg weekly through week 32. The placebo group received volume-matched saline injections on the same schedule. The trial protocol was approved by the Institutional Review Board, and all participants provided written informed consent. Sex was recorded by self-report at enrollment. Original clinical trial protocol available at ClinicalTrials.gov NCT04019197.

### Outcomes and assessments

Results for the primary and secondary outcomes were published^19^. While the parent trial’s primary outcomes were changes in adipose tissue volume (measured by CT) and body composition (DXA scans) at 32 weeks, our current analysis focuses on epigenetic aging markers as secondary/exploratory outcomes. PBMCs were collected by phlebotomy at baseline and week 32 visits, isolated by Ficoll gradient, and cryopreserved at −150 °C until analysis. Genomic DNA was extracted from thawed PBMC samples using Qiagen kits. Genome-wide DNAm profiling was performed using the Infinium MethylationEPICV2 BeadChip (Illumina), which covers >850,000 CpG sites, following manufacturer protocols. Briefly, 500 ng of genomic DNA per sample was bisulfite-converted (Zymo EZ DNA Methylation kit) and hybridized to the EPIC array, with arrays scanned on an iScan instrument to produce raw intensity data. To pre-process the methylation data, we used the R minfi v1.54.1 pipeline^46^, and low-quality samples were identified using the qcfilter() function from the R ENmix v1.44.3 package^47^, using default parameters. 100% of the original samples passed the QA/QC (*p* < 0.05) and were deemed to be high-quality samples.

### Epigenetic clock calculations

We examined three generations of epigenetic clocks: first-generation clocks that estimate chronological age (Horvath1, Horvath2, and Hannum clocks); second-generation clocks that predict mortality/morbidity risk (PhenoAge and GrimAge, including a principal-component version of GrimAge denoted PCGrimAge); and a third-generation clock known as DunedinPACE, which measures the pace of aging (years aged per chronological year). To improve robustness for longitudinal analysis, we used principal component-based versions of the first- and second-generation clocks (denoted PC clocks) wherever applicable. These PC clocks leverage principal components of DNAm data associated with the original clock algorithms, enhancing technical reliability and reducing noise in repeated measures. Published epigenetic clocks were calculated according to published methods from processed DNAm data. To calculate the principal component-based epigenetic clock for the Horvath multi-tissue clock, Hannum clock, DNAmPhenoAge clock, GrimAge clock, and telomere length, we used the custom R script available via GitHub (https://github.com/MorganLevineLab/PC-Clocks). Non-principal component-based (non-PC) Horvath, Hannum, and DNAmPhenoAge epigenetic metrics were calculated using the methyAge function in the ENMix R package. The pace of aging clock, DunedinPACE, was calculated using the PACEProjector function from the DunedinPACE package available via GitHub (https://github.com/danbelsky/DunedinPACE). Supplementary Fig. 1 shows cross-clock agreement with chronological age. We used a 12-cell immune deconvolution method to estimate cell type proportions^48^. Cell immune deconvolution results shown in Supplementary Fig. 3. For Biolearn, DNAm beta values (ssNoob-normalized) and matched sample metadata were imported into R (v4.2.2). Python integration was managed via the reticulate package, linking to a virtual environment with Biolearn installed. Missing CpGs were imputed using dataset-wide means via impute_from_average(), and the resulting matrix was combined with metadata into a GeoDataobject. The GrimAgeV1 and GrimAgeV2 models, obtained from the Biolearn ModelGallery, were applied using default parameters. Both models are based on Cox proportional hazards regression, trained to predict time-to-death from DNAm profiles. Internally, the models first extract a subset of CpGs relevant to DNAm surrogates for plasma proteins and smoking pack-years, followed by transformation through weighted linear combinations. These component predictors are then integrated into a multivariate Cox-PH model to estimate mortality risk, which is scaled to generate biological age equivalents.

### Statistics and reproducibility

Since this was a post hoc study, no statistical method was used to predetermine sample size for the epigenetic aging analyses. Participants were randomized 1:1 to semaglutide or placebo. Eight participants (15%) in each arm discontinued prematurely, leaving 92 who completed 32 weeks; of these, 84 had paired samples available for epigenetic analysis. No epigenetic data were excluded from the analyses. Epigenetic clock calculations were performed blinded to treatment allocation. Both participants and investigators were blinded to treatment assignment. For each participant and each clock, we computed the annualized rate of epigenetic age change by dividing the difference between the Week 32 and baseline epigenetic age estimates by the duration of follow-up expressed in years, yielding units of epigenetic years per calendar year. For DunedinPACE, which is already a per-year rate, we computed the change between Week 32 and baseline values (so a negative change indicates slowing of aging). Group comparisons of these rates were first assessed with Student’s *t*-tests (two-sided) for an initial view. The primary analysis used ANCOVA to estimate the effect of treatment (semaglutide vs. placebo) on epigenetic aging rate, adjusting for prespecified covariates. Our model for each aging measure included the baseline value of that measure (to adjust for regression to the mean), treatment group, sex, baseline BMI, baseline hsCRP, and baseline sCD163. Interaction terms between treatment and key baseline factors (e.g., baseline epigenetic age acceleration or baseline BMI) were explored to see if the treatment effect varied by these factors. Because the study is exploratory for epigenetic outcomes, we did not adjust *p*-values for multiple comparisons across the different clocks; instead, we focus on consistency of the pattern of results. Because of the imbalance in sex distribution between treatment arms with epigenetic data (67% male in the semaglutide group vs. 49% in placebo), sex was included as a covariate in all ANCOVA models. Sex-stratified subgroup analyses of epigenetic aging outcomes were not pre-specified and the study was not powered to detect sex-specific effects. All analyses were conducted in R (v4.2.2).

### Reporting summary

Further information on research design is available in the Nature Portfolio Reporting Summary linked to this article.

## Supplementary information

Supplementary Information

Reporting summary

Transparent Peer Review file

## Source data

Source data

## Data Availability

The DNA methylation data generated in this study for longitudinal semaglutide and placebo samples, have been deposited in the GEO database under accession number: GSE327270. The clinical trial data are available under restricted access due to data privacy laws. All controlled-access clinical trial datasets provided by Dr. McComsey can be applied for by emailing grace.mccomsey@uhhospitals.org. Data use agreements and institutional IRB will be required. The processesd data are available in the Source data. Source data are provided with this paper.

## Code availability

All epigenetic clock algorithms were applied using publicly available code and R/Python packages. Principal component–based epigenetic clocks (PCHorvath1, PCHorvath2, PCHannum, PCPhenoAge, PCGrimAge) were calculated using the PC-Clocks repository (https://github.com/albertchen42/PC-Clocks). Non-PC Horvath, Hannum, and DNAmPhenoAge clocks were calculated using the methyAge function in the ENmix R package (v1.44.3). DunedinPACE was calculated using the PACEProjector function from the DunedinPACE R package (v0.99.0; https://github.com/danbelsky/DunedinPACE). GrimAge V1 and V2 were computed using the Biolearn Python library (https://bio-learn.github.io/). OMICmAge was calculated using code available at https://github.com/LaskySuLab/OMICmAge/. CausAge, DamAge, and AdaptAge were calculated using algorithms available in the Supplementary Information of the original publication and via the ClockBase platform (www.clockbase.org) and the Biolearn Python package (https://bio-learn.github.io/). RetroAge was calculated using retroelement-based clock coefficients and scripts deposited on Zenodo (DOI: 10.5281/zenodo.11099870), which support DNA methylation EPIC v1.0 and v2.0 datasets. SystemsAge was calculated using the methylCIPHER package (https://github.com/HigginsChenLab/methylCIPHER). The Intrinsic Capacity epigenetic clock was calculated using code available at https://github.com/msfuentealba/IC_clock. DNAmFitAge and additional epigenetic metrics were calculated via TruDiagnostic’s DNAm Analysis Software, available at no cost for research purposes upon request (https://www.trudiagnostic.com/softwarerequest/); access requires agreement to a standard noncommercial software license for academic, nonprofit research use. DNA methylation preprocessing was performed using the minfi R package (v1.54.1). No custom analysis code beyond the application of these published tools was developed for this study.
