# Supplementary material for: Semaglutide slows epigenetic aging in a randomized trial of HIV-associated lipohypertrophy
Source: Nat Commun. 2026 May 19;17:6606. doi: 10.1038/s41467-026-72861-3 (PMC13381876; doi:10.1038/s41467-026-72861-3)
Supplement: Supplementary file 1 — Supplementary Information [file 41467_2026_72861_MOESM1_ESM.pdf]

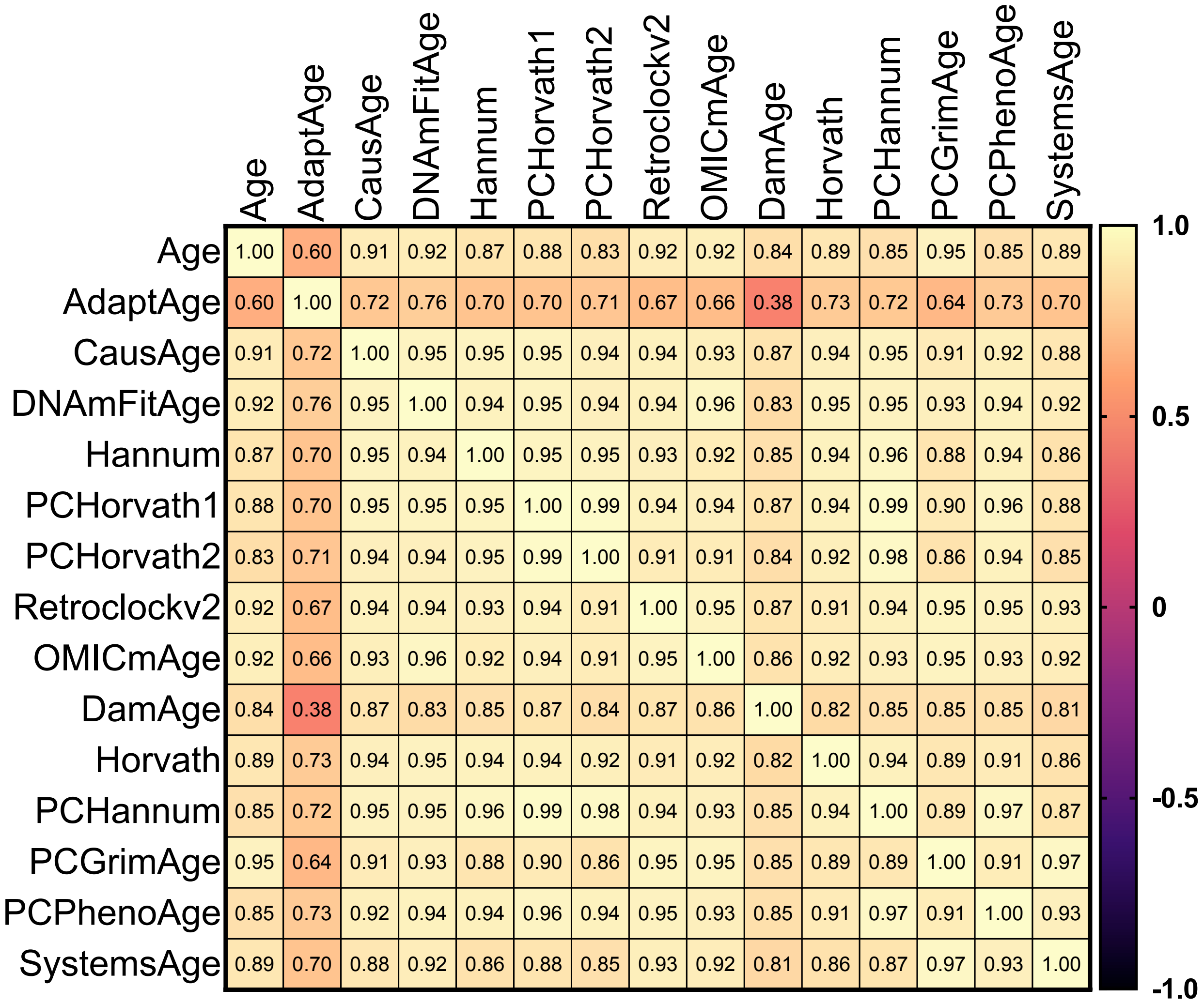

Supplemental Figure 1: Correlation matrix of participants observed chronological age across multiple epigenetic clocks. Heatmap showing pairwise Pearson correlation coefficients (*r*) between predicted epigenetic age (from each DNA methylation–based clock) and chronological age at baseline. Values within each cell represent correlation coefficients. The color scale ranges from dark purple (lowest correlation) through red to yellow (highest correlation, *r* = 1.00). Correlations were calculated using Pearson's product-moment correlation; P values, if reported, are two-sided and uncorrected for multiple comparisons. n = 84 biologically independent participants with baseline epigenetic age estimates.

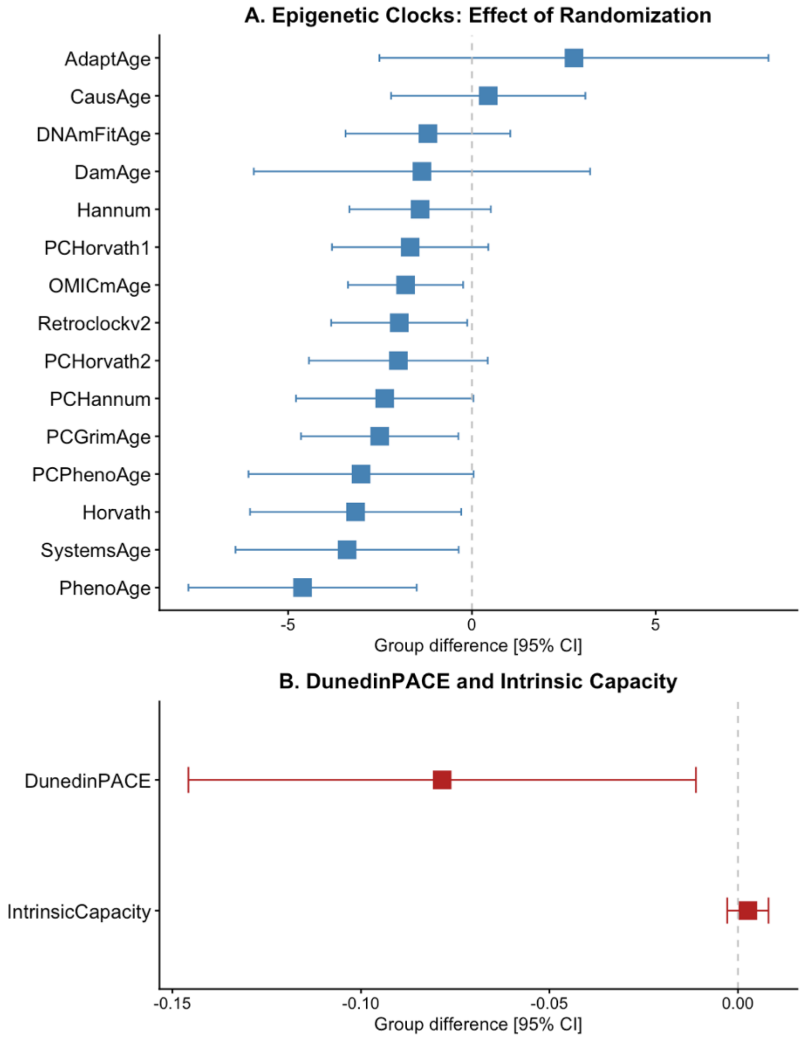

Supplemental Figure 2: Semaglutide broadly decelerates epigenetic aging across multiple methylation clocks (unadjusted analysis). Forest plot displaying the estimated difference (semaglutide minus placebo) in annualized epigenetic age change for DNA methylation–based biomarkers of aging. Point estimates (filled squares) represent mean between-group differences. Horizontal lines indicate 95% confidence intervals. The dashed vertical line at zero represents no between-group difference. **(A)** Effects on epigenetic clocks spanning first-generation (Horvath, Hannum), principal component–based (PCHorvath1/2, PCHannum, PCPhenoAge), and second- and third-generation (PhenoAge, PCGrimAge, AdaptAge, CausAge, DamAge, DNAmFitAge, OMICmAge, Retroclockv2, SystemsAge) epigenetic clocks. **(B)** DunedinPACE, a continuous pace-of-aging measure (units of years aged per chronological year), and Intrinsic Capacity epigenetic clock (IC score). Negative estimates indicate slower biological aging in the semaglutide group. n = 39 placebo and n = 45 semaglutide biologically independent participants, each with paired baseline and Week 32 epigenetic age estimates. No adjustment for multiple comparisons was applied, as these analyses were exploratory and post hoc. P values are two-sided.

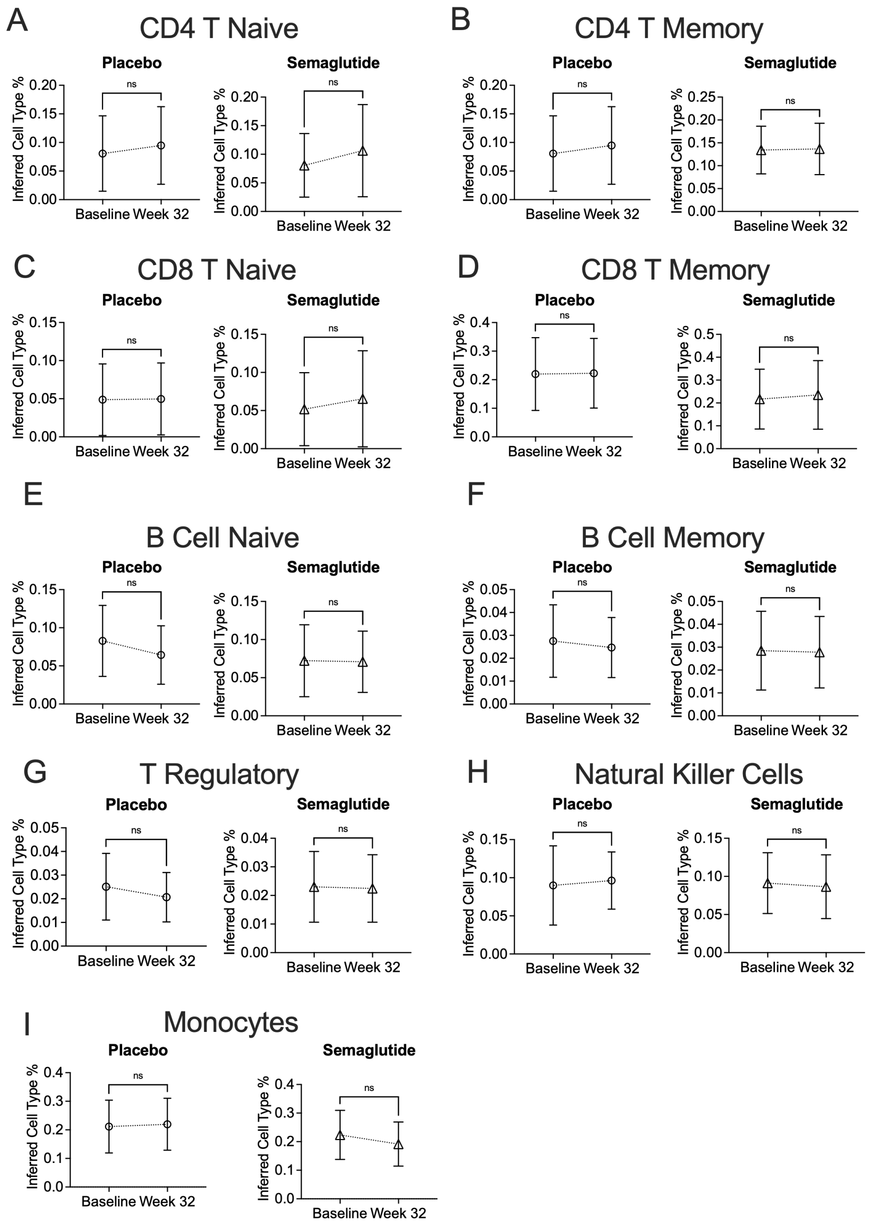

Supplemental Figure 3: Immune cell-type DNA methylation deconvolution analysis shows no significant differences between treatment groups over time. Estimated proportions of major peripheral immune cell populations were derived using a 12-cell-type DNA methylation deconvolution method and compared between placebo (circles) and semaglutide (triangles) groups at baseline and Week 32. Panels show **(A)** CD4 T naïve, **(B)** CD4 T memory, **(C)** CD8 T naïve, **(D)** CD8 T memory, **(E)** B cell naïve, **(F)** B cell memory, **(G)** regulatory T cells, **(H)** natural killer cells, and **(I)** monocytes. Error bars represent mean ± SEM. Within-group longitudinal comparisons (baseline vs. Week 32) were assessed using two-sided paired *t*-tests. None reached statistical significance. n = 39 placebo and n = 45 semaglutide biologically independent participants, each with paired baseline and Week 32 samples. P values are two-sided.

CONSORT 2010 Checklist

Completed for: Corley et al. Semaglutide Slows Epigenetic Aging in a Randomized Trial of HIV-Associated Lipohypertrophy

Section/Topic	Item No	Checklist item	Reported on page No
Title and abstract			
	1a	Identification as a randomised trial in the title	Title
	1b	Structured summary of trial design, methods, results, and conclusions (for specific guidance see CONSORT for abstracts)	Abstract
Introduction			
**Background and objectives**	2a	Scientific background and explanation of rationale	Introduction, ¶1–2
	2b	Specific objectives or hypotheses	Introduction, ¶3
Methods			
**Trial design**	3a	Description of trial design (such as parallel, factorial) including allocation ratio	Methods (Study Design); Results ¶1
	3b	Important changes to methods after trial commencement (such as eligibility criteria), with reasons	Methods (Study Design);
**Participants**	4a	Eligibility criteria for participants	Methods (Study Design); Results ¶1
	4b	Settings and locations where the data were collected	Methods (Study Design): single-centre, University Hospitals Cleveland Medical Center
**Interventions**	5	The interventions for each group with sufficient details to allow replication, including how and when they were actually administered	Methods (Study Design); Results ¶1
**Outcomes**	6a	Completely defined pre-specified primary and secondary outcome measures, including how and when they were assessed	Methods (Outcomes and Assessments); Results ¶1–2
	6b	Any changes to trial outcomes after the trial commenced, with reasons	Methods (Study Design); Results ¶1
**Sample size**	7a	How sample size was determined	Methods (Statistics): post hoc analysis; no predetermination for epigenetic outcomes
	7b	When applicable, explanation of any interim analyses and stopping guidelines	Not applicable
**Randomisation:**			
**Sequence generation**	8a	Method used to generate the random allocation sequence	Methods (Study Design): online randomisation software program
	8b	Type of randomisation; details of any restriction (such as blocking and block size)	Methods (Study Design): block sizes of six
**Allocation concealment mechanism**	9	Mechanism used to implement the random allocation sequence (such as sequentially numbered containers), describing any steps taken to conceal the sequence until interventions were assigned	Methods (Study Design): online randomisation system; masked to participants, investigators, and research personnel
**Implementation**	10	Who generated the random allocation sequence, who enrolled participants, and who assigned participants to interventions	Methods (Study Design): injections administered by experienced clinic nurses; randomisation via online system
**Blinding**	11a	If done, who was blinded after assignment to interventions (for example, participants, care providers, those assessing outcomes) and how	Methods (Study Design): double-blind; participants and investigators blinded; epigenetic clock calculations performed blinded

	11b	If relevant, description of the similarity of interventions	Methods (Study Design): volume-matched saline injections as placebo
**Statistical methods**	12a	Statistical methods used to compare groups for primary and secondary outcomes	Methods (Statistics): Exploratory epigenetic analyses for ANCOVA adjusting for sex, BMI, hsCRP, sCD163;
	12b	Methods for additional analyses, such as subgroup analyses and adjusted analyses	Methods (Statistics): Biolearn orthogonal analysis; systems clocks
Results			
**Participant flow (a diagram is strongly recommended)**	13a	For each group, the numbers of participants who were randomly assigned, received intended treatment, and were analysed for the primary outcome	Results ¶1; Figure 1 (CONSORT diagram): 154 screened → 108 randomised → 84 epigenetic analysis
	13b	For each group, losses and exclusions after randomisation, together with reasons	Results ¶1; Methods (Statistics): 8 per arm (15%) discontinued prematurely
**Recruitment**	14a	Dates defining the periods of recruitment and follow-up	ClinicalTrials.gov NCT04019197; 32-week follow-up
	14b	Why the trial ended or was stopped	Not reported (trial completed per protocol)
**Baseline data**	15	A table showing baseline demographic and clinical characteristics for each group	Table 1
**Numbers analysed**	16	For each group, number of participants (denominator) included in each analysis and whether the analysis was by original assigned groups	Results: n = 45 semaglutide, n = 39 placebo; analyzed as randomized
**Outcomes and estimation**	17a	For each primary and secondary outcome, results for each group, and the estimated effect size and its precision (such as 95% confidence interval)	Results: effect sizes with 95% CIs and P-values for all 17 clocks (Figures 2–5)
	17b	For binary outcomes, presentation of both absolute and relative effect sizes is recommended	Not applicable (all outcomes are continuous)
**Ancillary analyses**	18	Results of any other analyses performed, including subgroup analyses and adjusted analyses, distinguishing pre-specified from exploratory	Results: systems clocks (Figure 5); GrimAge V1/V2 via Biolearn (Figure 4); IC clock; immune cell deconvolution (Suppl. Fig. 3); all exploratory/post hoc
**Harms**	19	All important harms or unintended effects in each group (for specific guidance see CONSORT for harms)	Reported in parent trial (ref 19, Eckard et al. Lancet Diabetes Endocrinol 2024)
Discussion			
**Limitations**	20	Trial limitations, addressing sources of potential bias, imprecision, and, if relevant, multiplicity of analyses	Discussion, ¶5–7 (post hoc design, sample size, PBMC-only, no multiple comparison adjustment, ART history)
**Generalisability**	21	Generalisability (external validity, applicability) of the trial findings	Discussion, ¶5 and Abstract: HIV-specific cohort, 32-week follow-up may limit generalizability
**Interpretation**	22	Interpretation consistent with results, balancing benefits and harms, and considering other relevant evidence	Discussion, ¶1–4
Other information			
**Registration**	23	Registration number and name of trial registry	Methods; Results ¶1: ClinicalTrials.gov NCT04019197

**Protocol**	24	Where the full trial protocol can be accessed, if available	ClinicalTrials.gov NCT04019197; parent trial ref 19
**Funding**	25	Sources of funding and other support (such as supply of drugs), role of funders	Acknowledgments/Funding: NIH R01DK121619, CTSC UM1TR004528, Pendleton Trust, SD CFAR P30 AI036214

Effects of GLP-l Receptor Agonists on Cardiometabolic Alterations in HIV-associated Lipohypertrophy

Protocol Number: GLP1RA-HIV-LH

National Clinical Trial (NCT) Identified Number: 04019197

Principal Investigators: Grace McComsey MD, Allison Eckard MD

Principal Investigators:

Grace A McComsey, MD, FIDSA
Associate Chief Scientific Officer, University Hospitals Health System
Director, UH Clinical Research Center
Director, Dahms Clinical Research Unit
John Kennell Endowed Chair
Chief, Pediatric Infectious Diseases, and Rheumatology
Professor of Pediatrics and Medicine
Case Western Reserve University
216-844-5936

Allison Ross Eckard, MD, FIDSA, AAHIVS
Associate Professor of Pediatrics and Medicine
Divisions of Infectious Diseases
Medical University of South Carolina
135 Rutledge Ave, Ste 1217
Charleston, SC 29425-7520
Telephone 843-876-1920
Fax 843-792-6680

Table of Contents

STATEMENT OF COMPLIANCE 4
1. PROTOCOL SUMMARY 5
1.1 Synopsis 5
1.2 Schema 6
1.3 Schedule of Activities (SoA) 7
2 INTRODUCTION 8
2.1 Study Rationale 8
2.2 Background 8
2.3 Risk/Benefit Assessment 13
2.3.1 Known Potential Risks 13
2.3.2 Known Potential Benefits 14
2.3.3 Assessment of Potential Risks and Benefits 14
3 OBJECTIVES AND ENDPOINTS 18
4 STUDY DESIGN 20
4.1 Overall Design 20
4.2 Justification for Dose 21
4.3 End of Study Definition 21
5 STUDY POPULATION 22
5.1 Inclusion Criteria 22
5.2 Exclusion Criteria 24
5.3 Lifestyle Considerations 25
5.4 Screen Failures 25
5.5 Strategies for Recruitment and Retention 25
6 STUDY INTERVENTION 29
6.1 Study Intervention(s) Administration 29
6.1.1 Study Intervention Description 29
6.1.2 Dosing and Administration 29
6.2 Preparation/Handling/Storage/Accountability 30
6.2.1 Acquisition and accountability 30
6.2.2 Formulation, Appearance, Packaging, and Labeling 30
6.2.3 Product Storage and Stability 30
6.2.4 Preparation 30
6.3 Measures to Minimize Bias: Randomization and Blinding 31
6.4 Study Intervention Compliance 31
6.5 Concomitant Therapy 31
6.5.1 Rescue Medication 31
7 STUDY INTERVENTION DISCONTINUATION AND PARTICIPANT DISCONTINUATION/WITHDRAWAL 32
7.1 Discontinuation of Study Intervention 32
7.2 Participant Discontinuation/Withdrawal from the Study 32

7.3 Lost to Follow-Up 32
8 STUDY ASSESSMENTS AND PROCEDURES 33
8.1 Efficacy Assessments 33
8.2 Safety and Other Assessments 37
8.3 Adverse Events and Serious Adverse Events 38
8.3.1 Definition of Adverse Events (AE) 38
8.3.2 Definition of Serious Adverse Events (SAE) 38
8.3.3 Classification of an Adverse Event 38
8.3.4 Time Period and Frequency for Event Assessment and Follow-Up 35
8.3.5 Adverse Event Reporting 40
8.3.6 Serious Adverse Event Reporting 40
8.3.7 Reporting Events to Participants 40
8.3.8 Events of Special Interest 41
8.3.9 Reporting of Pregnancy 41
8.4 Unanticipated Problems 41
8.4.1 Definition of Unanticipated Problems (UP) 41
8.4.2 Unanticipated Problem Reporting 42
8.4.3 Reporting Unanticipated Problems to Participants 42
9 STATISTICAL CONSIDERATIONS 43
9.1 Statistical Hypotheses 43
9.2 Sample Size Determination 43
9.3 Populations for Analyses 43
9.4 Statistical Analyses 44
9.4.1 General Approach 44
9.4.2 Analysis of the Primary Efficacy Endpoint(s) 44
9.4.3 Analysis of the Secondary Endpoint(s) 45
9.4.4 Safety Analyses 45
9.4.5 Baseline Descriptive Statistics 45
9.4.6 Planned Interim Analyses 45
5
9.4.7 Tabulation of Individual participant Data 46
9.4.8 Exploratory Analyses 46
10 SUPPORTING DOCUMENTATION AND OPERATIONAL CONSIDERATIONS 47
10.1 Regulatory, Ethical, and Study Oversight Considerations 47
10.1.1 Informed Consent Process 47
10.1.2 Study Discontinuation and Closure 48
10.1.3 Future Use of Stored Specimens and Data 43
10.1.4 Key Roles and Study Governance 49
10.1.5 Safety Oversight 49
10.1.6 Clinical Monitoring 50
10.1.7 Quality Assurance and Quality Control 45

10.1.8 Data Handling and Record Keeping 51
10.1.9 Protocol Deviations 51
10.1.10 Publication and Data Sharing Policy 51
10.1.11 Conflict of Interest Policy 52
10.2 Additional Considerations 47
10.3 Abbreviations 52
10.4 Protocol Amendment History 54
11 REFERENCES 49

STATEMENT OF COMPLIANCE

The trial will be conducted in accordance with International Conference on Harmonisation Good Clinical Practice (ICH GCP), applicable United States (US) Code of Federal Regulations (CFR), and the National Institute of Diabetes and Digestive and Kidney Diseases (NIDDK) terms and Conditions of Award. The Principal Investigators will assure that no deviation from, or changes to the protocol will take place without prior agreement from the Investigational New Drug (IND) or Investigational Device Exemption (IDE) sponsor, funding agency and documented approval from the Institutional Review Board (IRB), except where necessary to eliminate an immediate hazard(s) to the trial participants. All personnel involved in the conduct of this study have completed Human Subjects Protection and ICH GCP Training.

The protocol, informed consent form(s), recruitment materials, and all participant materials will be submitted to the IRB for review and approval. Approval of both the protocol and the consent form must be obtained before any participant is enrolled. Any amendment to the protocol will require review and approval by the IRB before the changes are implemented to the study. All changes to the consent form will be IRB approved; a determination will be made regarding whether a new consent needs to be obtained from participants who provided consent, using a previously approved consent form.

1. PROTOCOL SUMMARY

1.1 SYNOPSIS

**Title:**	Effects of GLP-l Receptor Agonists on Cardiometabolic Alterations in HIV-associated Lipohypertrophy
**Study Description:**	In this study, we will conduct a randomized, double-blinded, placebo-controlled trial to assess the effect of GLP-1 receptor agonist (semaglutide) on visceral and ectopic fat, insulin resistance, inflammation markers, and the downstream effect on cardiovascular risk in HIV-infected individuals.
**Objectives:**	**Primary Objective:** To characterize the effects of semaglutide on visceral and ectopic fat and elucidate potential mechanisms by which these effects occur in HIV-infected individuals with lipohypertrophy.<br>**Secondary Objectives:** To examine the effect of semaglutide on lean body mass, inflammation, immune activation, gut integrity, and cardiovascular disease risk in HIV-infected individuals with lipohypertrophy and assess the sustainability of semaglutide effects after treatment discontinuation.
**Endpoints:**	**Primary Endpoints:** Visceral and ectopic fat changes at 32 weeks.<br>**Secondary Endpoints:** Changes in lean body mass, markers of inflammation, immune activation, gut integrity, and cardiovascular disease assessment after 32 weeks of treatment and changes in outcomes 24 weeks after treatment discontinuation (at 56 weeks).
**Study Population:**	104 HIV-infected individuals with lipohypertrophy<br><br>Participants from all ethnic, racial, and sex/gender categories will be eligible and included, with the goal of an equal proportion of males and females and half minority populations.
**Phase:**	2b
**Description of Sites/Facilities Enrolling Participants:**	Participants will be enrolled at the HIV clinic at Case Western Reserve University in Cleveland, OH.
**Description of Study Intervention:**	Semaglutide 1.0 mg given by subcutaneous injection once weekly, or matching placebo, for 32 weeks.
**Study Duration:**	5 years.
**Participant Duration:**	56 weeks.

1.2 SCHEMA

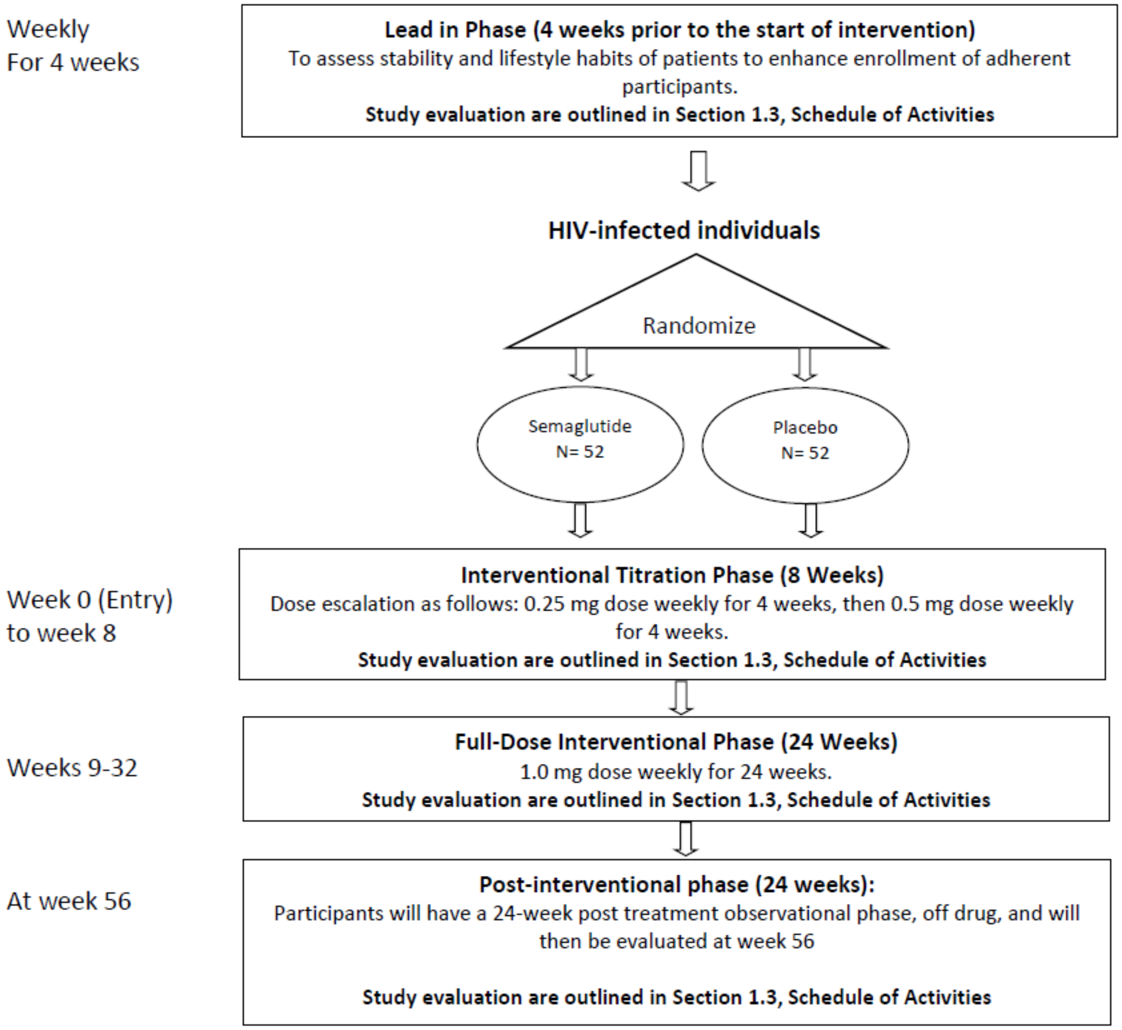

1.3 SCHEDULE OF ACTIVITIES (SOA)

	Screening	Lead-In Phase				Interventional Phase									
							Titration Phase		Full dose Phase						Post TX FU
Evaluation (weeks)		-4	-3	-2	-1	0	T1-T8	weekly*	9	14	20	26	31	32	56
Written consent, inclusion/exclusion	X														
Family history of CVD, diabetes, HTN						X								X	X
Safety monitoring							X	X	X	X	X	X	X	X	
Smoking, alcohol, illicit drug history						X					X			X	X
Detailed dietary, physical activity		X	X	X	X	X					X			X	X
Height, weight, anthropometric measurements,	X	X	X	X	X	X			X		X			X	X
blood pressure/pulse	X	X	X	X	X	X	X (wk 5)		X		X			X	X
Study drug SC injection by trained professional						X	X	X	X	X	X	X	X		
Hematology, chemistries, lipase, urine (b-HCG)	X					X			X	X	X	X		X	X
HgbA1C						X			X					X	X
HIV-1 RNA and CD4 count	X					X								X	X
Lipids, HOMA-IR						X			X					X	X
Oral glucose tolerance test						X			X		X			X	X
DXA (whole body)						X								X	X
CT scan abdomen (VAT, SAT, TAT)						X								X	X
Calcium Score						X								X	X
Adipokines						X					X			X	X
Gut hormones (4-hour mixed-meal tolerance test)					X								X		X
Gut integrity markers						X								X	
Natriuretic peptides						X					X			X	X
Resting energy expenditure					X				X		X			X	X
Inflammation and immune activation						X					X			X	X
AGE, EndoPat					X									X	X
Cognivue						X								X	X
Pulse wave velocity						X								X	X
plasma, serum, urine, stool storage						X			X		X			X	X
PBMC						X					X			X	X

* Weekly visits will be from week 9-32
*Fasting required for visits -1, 0, wk 9, wk 20, wk 31, wk 32, and wk 56
*Week 9 Chem 23, lipase, oral glucose ONLY

*Premature study discontinuation-week 56 evaluations will be followed
*Cognivue assessment +/- 4 weeks

2 INTRODUCTION

2.1 STUDY RATIONALE

Despite the advent of safer antiretroviral therapy agents with low potential for mitochondrial toxicity, lipohypertrophy (accumulation of central and ectopic fat) remains a common and significant challenge facing HIV providers and threatens the well-being of individuals living with HIV. Limited progress has been made in understanding and managing lipohypertrophy. Initially linked to the use of protease inhibitors (PIs), we have recently reported similar gains in peripheral and central fat after initiation of successful HIV treatment with both PIs and integrase inhibitors. These observations have challenged current beliefs and raised the concerns that fat accumulation may indeed be due to HIV itself, directly and/or indirectly, through the heightened inflammatory state that accompanies HIV. The role of alteration in gut hormone secretion and in gut epithelial barrier dysfunction in HIV-associated metabolic disorders is largely unknown, but it is plausible since chronic inflammation (such as that seen in HIV) has been shown to affect gut hormone secretions and gut integrity markers.

Studies of glucagon-like peptide-1 receptor agonists (GLP-1RAs) in diabetics have shown them to be safe, well-tolerated, with very low to no concerns about drug-drug interactions, and, importantly, have caused weight loss that occurs preferentially via losses in visceral fat. Some GLP-1RAs have even shown to decrease clinical CVD events in diabetics. Thus, this promising class of drugs may offer a powerful tool to fight the triple threat facing the success of long-term HIV treatment: namely, 1) excess fat accumulation and ectopic fat deposition, 2) insulin resistance and a high prevalence of diabetes, and 3) endothelial dysfunction and cardiovascular disease (CVD) risk. GLP-1RAs act by partially-delineated mechanisms, several of which will be studied in this proposal.

We will conduct a randomized, double-blinded, placebo-controlled clinical trial to assess whether a potent and safe GLP-1RA may positively affect visceral fat and ectopic fat accumulation, insulin resistance, inflammation markers, and the downstream effect on CVD in people living with HIV.

2.2 BACKGROUND

Significance of the trial:

Body composition alterations in HIV: Despite new, safer antiretroviral therapy (ART), certain aspects of adipose tissue alterations, previously termed "lipodystrophy," continue to frequently occur in HIV-1. With older ART, specifically, thymidine nucleoside reverse transcriptase inhibitors (NRTIs), fat abnormalities were dominated by lipoatrophy, a devastating loss of fat from limbs and face that subsequently was linked by us and others to mitochondrial toxicity.[1-3] Subsequent avoidance of these NRTIs led to an abrupt decrease in the incidence of lipoatrophy.[4,5] However, the other facet of fat alterations, namely lipohypertrophy (or abdominal visceral fat accumulation), frequently associated with insulin resistance and ectopic fat deposition in the liver and pericardial regions, continues to be very prominent and a substantial threat to the success of ART. That is, these fat alterations not only cause major cosmetic concerns, but they are also associated with heightened inflammation and increased risk of cardiovascular disease (CVD).[6] Initially thought to be linked to the use of protease inhibitors (PIs), we recently showed that subjects initiating therapy with integrase inhibitors (INSTI) suffered the same extent of visceral fat accumulation when compared to subjects initiating PI-based therapies (see preliminary data section).[7] Moreover, we recently showed that the odds of severe weight gain (generally defined as gains of ≥10% in weight over 96 weeks after ART initiation) were higher when subjects initiated HIV therapy with INSTI versus PIs.[8] In these studies, we also showed that viral load prior to ART initiation was a main independent predictor of fat gain, in that subjects starting therapy with an HIV-1 RNA >100,000 copies/mL (or in a high viral load stratum) experienced 2 to 3 times the fat gain of those starting at a lower viral load, regardless of the ART drug class. In addition, levels of inflammatory markers such as interleukin-6 (IL-6) were positively correlated with total fat changes. These studies illustrate the fact that lipohypertrophy remains an important issue for people living with HIV (PLWH) and that HIV-related factors are important determinants of these fat alterations.

Ectopic fat depots: Besides visceral fat accumulation, ectopic lipid deposition has been implicated in the pathophysiology of insulin resistance and obesity-related disorders. Hepatic fat is strongly correlated with the amount of visceral adipose tissue (VAT) in a prediabetic population[9] and has been associated with CVD independent of classical risk factors.[10] Cardiac ectopic fat can secrete cytokines like other visceral fat and may contribute to detrimental cardiac remodeling.[11-13] In fact, epicardial fat (adipose tissue located between the myocardium and the visceral pericardium; also known as the visceral pericardium) has been shown to contain more mRNA and protein for IL-1β, IL-6, monocyte chemoattractant protein-1, and TNF-

α than subcutaneous adipose tissue (SAT), and cytokine concentrations are significantly correlated with the accumulation of inflammatory cells, such as T-lymphocytes and macrophages.[14,15] Ectopic cardiac fat volume represents a novel marker of CVD risk.[16] In fact, pericardial fat volume actually is better correlated with coronary calcifications than VAT.[17] We have shown that in PLWH on ART, pericardial fat was associated with insulin resistance (independent of VAT) and with the presence of coronary calcifications.[18,19]

Management of lipohypertrophy in HIV: A challenge in the modern treatment era: The mechanism and management of lipohypertrophy continue to present major challenges to HIV providers and PLWH. Diet and exercise have not been successful at reversing fat accumulation, nor were switches between different ART classes. Currently one medication, the growth hormone-releasing hormone, tesamorelin, is FDA-approved specifically for the treatment of lipohypertrophy in HIV; however, it is not widely used due to its high cost, the transient nature of the improvement in fat (which returns to baseline after discontinuation of the drug), and the concern about worsening insulin resistance. The FDA also warns that the long-term cardiovascular safety and potential long-term CVD benefit are currently unknown. Lastly, tesamorelin does not significantly decrease weight, a downside in the current obesity epidemic, which includes PWLW.[20] Thus, novel therapeutic interventions that could decrease weight, attenuate VAT accumulation, and potentially improve CVD risk are urgently needed.

GLP-1RA: a multifaceted approach to treat cardiometabolic abnormalities in HIV: Glucagon-like peptide-1 (GLP-1) is a naturally-occurring neuropeptide and an incretin secreted from intestinal L cells in response to the presence of nutrients in the lumen of the small intestine. GLP-1 has potent blood glucose-lowering action only during hyperglycemia, as it induces insulin secretion, stimulates insulin sensitivity, reduces glucagon secretion in a glucose-dependent manner, and improves β-cell function.[21-24] The first glucagon-like peptide-1 receptor agonist (GLP-1RA), exenatide, was approved in the United States in 2005 as an adjunct to diet and exercise to improve glycemic control in adults with type 2 diabetes mellitus. There are now multiple GLP-1RAs available and are used extensively in clinical practice in this population. GLP-1RAs are well-tolerated, improve glycemic control with a low risk of hypoglycemia, improve indices of β-cell function, improve systolic blood pressure, and are associated with sustained weight loss.[25-30] With obesity and diabetes frequently occurring in tandem, weight loss became a desired side effect of the drug. The FDA approved the GLP-1RA, liraglutide, in 2014 for chronic weight management in obese (body mass index (BMI) ≥30 kg/m^2) or overweight (BMI ≥27 kg/m^2) adults who have ≥1 weight-related comorbidity such as hypertension, diabetes, or dyslipidemia as an adjunct to diet and exercise. In the 56-week SCALE trial, non-diabetic obese or overweight subjects were randomized to placebo vs. liraglutide at 3 mg daily, a higher dose than the 1.2-1.8 mg used in diabetics.[31] Liraglutide provided significantly greater weight loss of 8% vs. 2.6% in placebo (P<0.0001). No serious adverse events occurred; hypoglycemia events were rare, and none required professional intervention. The durability and excellent safety record of GLP-1RAs were also shown in a 5-year study of weekly exenatide subcutaneous injections; the weight reduction effect was sustained, and there were no major hypoglycemia or safety concerns. Importantly, the GLP-1RAs, semaglutide and liraglutide, have been shown to improve cardiovascular outcomes in type 2 diabetes.[32-34]

<u>Preliminary data:</u> **Lipohypertrophy in the era of contemporary ART is linked to higher HIV-1 RNA and not to ART class:** We investigated the % change in abdominal SAT and VAT by CT scan in a 96-week study of 328 HIV-infected, ART-naïve participants who received tenofovir disoproxil fumarate-emtricitabine (TDF/FTC) and were randomized to receive either a PI (ritonavir-boosted atazanavir (ATV/r) or darunavir (DRV/r)) or the INSTI, raltegravir (RAL), as part of the AIDS Clinical Trials Group (ACTG) study, A5260s, a sub-study of A5257.36 At week 96, a 30% increase in VAT was seen in all arms regardless of regimen (Figure 1). Participants with a baseline HIV-RNA >100,000 copies/mL had double to triple the amount of VAT gain compared to those with lower HIV-RNA levels, despite no observed difference in VAT changes based on ART regimen. In an analysis of the large parent study A5257, we found that the odds of severe weight/BMI gains (≥10% gains from baseline) over 96 weeks of ART, were higher for subjects initiating RAL when compared to the PIs.[8] These studies highlight the current unfortunate reality that no available HIV treatment is void of metabolic complications, and total and visceral fat accumulation occurs with all available ARTs.

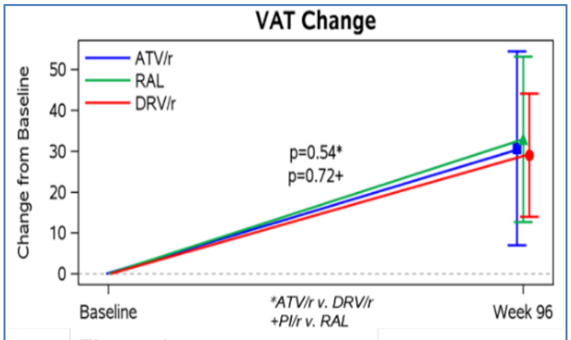

Figure 1.

<u>**Enhanced inflammation in HIV:**</u> We investigated the effect of ART on systemic inflammation by evaluating changes in inflammatory markers with ART initiation in ACTG 5224s, the metabolic sub-study of A5202, in which 1857 HIV-infected, ART-naïve PLWH were randomized to receive either abacavir (ABC)/lamivudine (3TC) or TDF/FTC with either efavirenz (EFV) or ATV/r.37 By week 96, soluble tumor necrosis factor receptor (sTNFR)-I, sTNFR-II, soluble vascular cellular adhesion molecule-1 (sVCAM-1), and soluble intercellular adhesion molecule-1 (sICAM-1) decreased significantly within each arm but without significant differences between arms. However, overall, high-sensitivity C-reactive protein (hsCRP) did not decrease significantly, and, in fact, at 96 weeks, participants who received ABC/3TC/EFV had a significantly higher level of hsCRP than at baseline.

<u>**Pre-ART and time-updated inflammation markers predict clinical events**</u>: In A5224s, we assessed the relationship between inflammation markers and incident clinical events.38 Pre-ART hsCRP, IL-6, sTNFR-I, and sTNFR-II were significantly associated with an increased risk of clinical events (even after adjustment for ART, CD4 count, and HIV RNA). Moreover, on-study changes in sTNFR-I and sTNFR-II were significantly associated with increased event risk, independently of ART, CD4 count, and HIV RNA.

<u>**Inflammation markers are correlated with diabetes and CVD markers in HIV**</u>**:** We measured inflammation markers and carotid intima-media thickness (cIMT), a surrogate measure of subclinical atherosclerosis, in 94 PLWH and HIV-uninfected, matched controls.39 cIMT, hsCRP, IL-6, and sVCAM-1 were significantly higher in PLWH. Also, hsCRP, TNF-α, and sVCAM-1 were independently associated with cIMT. In another study,[40] we found that at week 48 after ART initiation, PLWH in the highest quartile of sTNFR-I had increased odds of developing diabetes, compared to those in the lowest, even after adjustment for age, CD4, BMI, and PI use (Figure 2). Similar results were seen for sTNFR-II. Particularly pertinent to this current application, among measured inflammatory markers, only TNF-α receptors were predictive of incident diabetes,

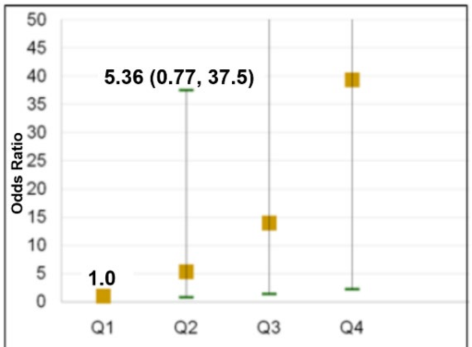

Figure 2: Odd Ratios for Incident Diabetes at Week 48 by Quartile of sTNFR-I

supporting the hypothesis that chronic TNF-α exposure in HIV could lead to decreased GLP-1 secretion, which ultimately could cause abnormalities in glucose metabolism.

Trunk fat correlates with monocyte activation and systemic inflammation: As part of a randomized, placebo-controlled trial of rosuvastatin in 147 PLWH on ART with mostly undetectable HIV RNA and with low-density lipoprotein (LDL) cholesterol <130 mg/dL, measurements were obtained at study entry for trunk and limb fat (as measured by dual-energy x-ray absorptiometry (DXA)), monocyte activation markers (sCD14 and sCD163), and markers of systemic inflammation. Overall, 50% of participants were on a PI-containing regimen. Trunk fat correlated with insulin resistance (as measured by homeostatic model assessment of insulin resistance (HOMA-IR); r=0.58, P<0.0001), sCD14 (r=0.19, P=0.018), sCD163 (r=0.23, P=0.005), hsCRP (r=0.30, P<0.0001), IL-6 (r=0.20, P=0.017), and sTNFR-I (r=0.26, P=0.0015). The study supports the hypothesis that central fat accumulation in HIV is linked to monocyte activation and systemic inflammation.

Importance of the trial:

The pathogenesis of lipohypertrophy in HIV remains elusive. Obtaining visceral adipose tissue from PLWH has been a major hurdle and cannot be done electively as part of a human subjects research protocol (without another medical reason necessitating surgery). Past investigations have focused on the role of ART agents (in particular, PIs) on the central fat accumulation seen in successfully-treated HIV infection. Recently, it became apparent that fat accumulation is not specific to any class of antiretrovirals, and, thus, the focus has shifted to include the possible direct and indirect effects of the virus, which are known to cause a chronic inflammatory state. Yet, the pathogenesis remains elusive. Even more, it remains unclear whether the excess visceral fat accumulation in HIV is merely due to a “return to health” phenomenon with an accompanying improvement in appetite and normalization of the resting energy expenditure (REE) after successful ART. And, therefore, simply represents a manifestation of obesity in PLWH.

For the first time in HIV, we will be able to carefully study the secretion of two gut hormones, GLP-1 and glucose-dependent insulinotropic polypeptide (GIP), stratified by HIV status. These metabolic hormones are released from the gut upon nutrient ingestion and are known for their insulin-stimulating effects, as well as their link to visceral fat accumulation and obesity. These have never been assessed comprehensively in PLWH in conjunction with measurements of visceral fat and glucose metabolism. In addition, since chronic inflammation (such as that seen in HIV) has been shown to alter the secretion of these hormones, we will also be able to study the relationship between these metabolic gut hormones, markers of inflammation and immune activation, and metabolic endpoints (VAT and insulin resistance). The data acquired from our proposed study will not only shed light on the pathogenesis of HIV-related lipohypertrophy but will also investigate a novel therapeutic intervention that could attenuate this concerning phenomenon and its associated co-morbidities.

Regardless of the underlying causes, there is a pressing need for effective treatment of the body composition changes and cardiometabolic abnormalities associated with HIV infection. Evidence-based guidelines to manage these complications are lacking, as is the understanding of the consequences of reversing the excess fat (VAT and ectopic fat) on the downstream CVD risk. We and others have previously shown that several single-agent strategies (e.g. growth hormone-releasing factor, glitazones, metformin, and statins) have each proven to be of partial benefit on certain aspects of HIV-related cardiometabolic abnormalities. However, a single therapy is needed to comprehensively reverse the numerous abnormalities contributing to this increased cardiometabolic risk among PLWH. GLP-1RAs are appealing candidates for such a therapy because of their proven multifaceted effect in the obese (HIV-uninfected)

population, and, specifically, in light of the recent finding that a heightened inflammatory state (such as seen in HIV) impairs secretion of GLP-1.[35] Thus, GLP-1RAs could be an excellent tool to fight the triple threat facing the success of long-term HIV management: 1) excess fat accumulation and ectopic fat deposition, 2) insulin resistance and high prevalence of diabetes, and 3) heightened inflammation, endothelial dysfunction, and CVD. The planned comprehensive studies will assess the efficacy and safety of GLP-1RA in PLWH, the mechanisms by which GLP-1RA improves the metabolic profile, and the downstream effects on CVD risk in HIV.

There are multiple reasons why studying GLP1-RAs in the HIV population is of paramount importance. Despite a proven safety and efficacy record of GLP-1 RAs in HIV-uninfected populations, such data are lacking in the setting of HIV infection. PLWH represent an aging population with a high risk for metabolic and cardiovascular alterations. All PLWH are currently maintained on a combination of antiretroviral drugs, none of which have been proven to be benign on cardiometabolic indices. In addition, even virologically-controlled HIV infection is associated with a state of heightened inflammation and immune activation which may alter gut hormone secretion and, thus, the efficacy of the GLP1RAs. In addition, the proven beneficial CVD effect of GLP-1RA among diabetics led to the FDA approving the use of some of these drugs for CVD prevention in this population. HIV itself and/or antiretrovirals have been associated with endothelial dysfunction, arterial stiffness, and a high risk of CVD events. It is crucial to study the effect of GLP1-RAs in the setting of HIV infection, where heightened inflammation and metabolically-toxic antiretrovirals could significantly alter the efficacy of the intervention. In other words, we cannot extrapolate results from interventions (like GLP1-RAs) in HIV-uninfected populations and apply them to our patients with HIV who are very different. The addition of a parallel study, with similar intervention and evaluations, in an HIV-uninfected control group will add insight to the specificity of the intervention by HIV status.

2.3 RISK/BENEFIT ASSESSMENT

2.3.1 KNOWN POTENTIAL RISKS

The potential risks for taking part in this study are:

- **Risk of loss of confidentiality:** There is a risk of loss of confidentiality. We will make every effort to minimize this risk (section below and 10.1.3 outline the different measures taken to decrease this risk).

- **Blood tests:** There is a minimal risk associated with obtaining blood, including discomfort, bruising, bleeding, fainting, and infection.

- **Risks of fasting:** Some individuals find fasting or abstaining from smoking or caffeine to be bothersome. It may make some individuals feel anxious, irritable, or hungry. Participants who are required to take their morning medications with food should wait until after the visit has been completed to take their medications.

- **Risk of 4-hour MMTT:** Some participants may feel a temporary sense of abdominal fullness and discomfort. Some may also have nausea, vomiting or diarrhea, but this is rare. Participants may also not like the taste of the Ensure Plus® shake, and it may leave an unpleasant taste in their mouth.

- **Risks of radiation from abdominal CT scan (for visceral fat and liver fat assessments):** We are exposed to radiation daily both from natural (sun and earth) and manmade sources. The average radiation dose from these sources for those living in the United States is 363 millirems per year. Exposure of up to 5,000 millirems of radiation is allowed in individuals who use radiation in their work (such as radiologic technologists and radiologists). Moreover, there is no evidence that a dose up to 5,000 millirems per year is associated with any risk. By comparison, the radiation dose that participants will receive from a CT scan of the abdomen (liver and fat) in this study is approximately 590 millirems (390 for the limited liver and 200 for single slice for fat). The scanning machines will not cause any physical discomfort other than from having to lie still on the table for the duration of the test.

- **Coronary CT scan for measurements of pericardial fat:** A non-contrast, coronary CT scan will be utilized for measurements of pericardial fat. The dose of radiation used with this new technology is estimated to be 80 mrems, which is less than 1/10th of a diagnostic CT scan of the thorax.

- **Whole-body DXA scan:** Measurements of whole-body DXA for assessments of fat and lean mass is negligible (~3 mrems of radiation).

- **PWV:** This is a non-invasive test and does not carry any known risks.

- **Semaglutide intervention:** The most commonly reported side effects noted with semaglutide are gastrointestinal-related, including nausea, vomiting, diarrhea, abdominal pain, and constipation, which tend to improve with time. Minor hypoglycemia (suggestive symptoms and glucose <55 mg/dL) occur in a very small number of people. Hypersensitivity reactions to semaglutide or any of its components can occur and, in studies, occurred at a rate of 1%. In all GLP-1RAs, injection site reactions (mostly pruritis, tenderness, nodule or redness) are possible, but tend to be mild and occur in similar frequency to placebo injections. Injection site reactions were only reported in 0.2% of semaglutide-treated subjects in clinical trials. Renal dysfunction has also been described, usually in people with known pre-existing renal disease. Use of GLP-1RAs can also increase the risk of gallbladder and bile duct disease, and cholelithiasis has been reported with the use of semaglutide. Semaglutide was also associated with an increase in retinopathy complications in diabetics in a small percentage of participants over the course of a 2-year trial with diabetes (3% compared to 1.8% in placebo over a 2-year trial. Rare cases of pancreatitis have been reported with the use of GLP-1RAs. Other adverse reactions that have been reported in small numbers include fatigue, dysgeusia, and dizziness. Semaglutide may also slow gastric emptying (gastroparesis) which could potentially impact absorption of concomitantly administered oral medications. In clinical trials, a mean increase in heart rate of 2-3 beats/minute were reported with semaglutide. The FDA has added a warning in the package insert for all GLP-1RAs about a possible increased risk of thyroid cancers, specifically medullary thyroid carcinoma. Data in this regard come from research in rodents and happened in a dose- and duration-dependent manner. It is unclear how this risk will translate in human use. There is no evidence of such tumors in semaglutide-treated subjects; however, such (rare) cases have been observed with the use of another GLP-1RA, liraglutide.

- **Cognivue** is an FDA-cleared computerized testing tool rooted in adaptive psychophysics and designed to assess early signs of cognitive impairment. It received FDA-clearance in June of 2015 as a Computerized Cognitive Assessment Aid Class 2 Medical Device.

Cognivue® consists of 3 sub-batteries of 10 separately scored sub-tests presented in a 10-minute automated sequence. The first sub-battery measures visual salience and adaptive measures adaptive motor control. These results do not count towards the final score. They are used only to adapt the remaining sub-tests to the response characteristics of the individual subject. This ensures that only cognition is evaluated, and the subject is not at a disadvantage because of visual or motor deficits. The subsequent 2 sub-batteries include perceptual processing (letter, word, shape, and motion discrimination), and memory processing (letter, word, shape, and motion memory). After the test, a two-page report is provided: it includes an overall score, with a subsequent breakdown into six key cognitive domains aligned with current standards: visuospatial, executive function, naming/language, memory, delayed recall, and abstraction. The second page has information on 2 additional important parameters: Reaction Time and Speed Processing. There are no risks associated with this test. Subjects will be provided with the questionnaire on a device in a private room in the SIU and asked to complete the questions. This device uses a turn wheel for patients to answer the questions to their individual pace. A two-page report will be provided to the study team. These results will not be shared with subject or provider as it is not a clinical test, and no clinical intervention will result. The lower the score the higher the risk of neurocognitive impairment is present however the goal is to identify the mild impairment which is has an unclear clinical significance. Cognitive impairment can be a result from many underlying conditions including inflammation, metabolic conditions and lifestyle choices. It has been shown in HIV that co-morbidities have led to higher neurocognitive dysfunction and higher inflammation. We believe that modulating inflammation may be able to improve neurocognitive dysfunction.

2.3.2 KNOWN POTENTIAL BENEFITS

Study participants may not benefit from the intervention. So far, the management of lipohypertrophy in HIV remains unclear, and this study will comprehensively assess a potential strategy that could improve both fat alterations, insulin resistance, and cardiovascular disease risk.

In the HIV-uninfected population GLP-1RAs are well-tolerated, improve glycemic control with a low risk of hypoglycemia, improve indices of β-cell function, improve systolic blood pressure, and are associated with sustained weight loss.[25-30]

The durability and excellent safety record of GLP-1RAs were also shown in a 5-year study of weekly exenatide subcutaneous injections; the weight reduction effect was sustained, and there were no major hypoglycemia or safety concerns. Importantly, semaglutide has been shown to improve cardiovascular outcomes in type 2 diabetes on the long term.[32-34]

This study is very important to the HIV population, as currently the pathogenesis of HIV-related lipohypertrophy is not understood. We think that the potential for benefit outweighs the risk of participating in this study because of the low potential for adverse events.

2.3.3 ASSESSMENT OF POTENTIAL RISKS AND BENEFITS

There are multiple reasons why studying GLP-1RAs in the HIV population is of paramount importance. There is a pressing need for effective treatment of the body composition changes and cardiometabolic abnormalities associated with HIV infection. Evidence-based guidelines to manage these complications are lacking, as is the understanding of the consequences of reversing the excess fat (VAT and ectopic fat)

on the downstream CVD risk. Despite a proven safety and efficacy record of GLP-1RAs in HIV-uninfected populations, such data are lacking in the setting of HIV infection, and results from HIV-uninfected individuals cannot simply be extrapolated to the HIV-infected population.

HIV-infected individuals represent an aging population with a high risk for metabolic and cardiovascular alterations. The potential benefits of GLP1-RAs in HIV-infected individuals on fat accumulation, inflammation, immune activation and CVD prevention outweigh the risk of adverse events associated with this drug and the risk associated with this study protocol.

To minimize the known potential risks as described in Section 2.3.1, the following measures will be employed to protect participants:

- **Confidentiality:** We will make every effort to minimize this risk. Only IRB-approved research staff will have access to identifiable private information about the participants. Patient clinical and laboratory data will be collected on standardized forms which will be kept in locked filling cabinets in locked offices and/or stored on HIPAA-compliant, password-protected, cloud-based storage system "Box" and/or REDcap and only accessible to study staff. Each participant will be assigned a coded number, and this number will be used to identify the participant. All laboratory specimens, evaluation forms, reports, and other records will be identified by coded number only to maintain participant privacy. All computer entry and networking programs will be done with coded numbers only. Any material or data sent to non-study staff personnel will contain only de-identified information. No names or personal information will be used to identify the subjects. Clinical information will not be released to the participant's private physician, clinic, or any other entity without permission of the subject, except as necessary for monitoring by the IRB, FDA, NIH, or as required by law. Release of information will be documented from subjects before requesting any medical records from outside clinics, and associated documents will only be sent/received from HIPAA-compliant, secured fax lines. Study staff will only talk to participants or participants' representatives when phone calls are made for recruitment, retention, or adherence reasons, and any message left will have no mention of HIV. Recruitment material or any information given to participants (e.g., date reminders for study visits) will not mention HIV. Any publication of this study will not use the participant's name or any personal identifiers. Both study sites will ensure that appropriate measures are taken to protect the privacy and confidentiality of all personally-identifiable health information (PHI) for which it is responsible. The proposed studies will include compliance with all regulations set forth by the HIPAA Privacy Rule, as well as with existing State and Federal laws pertaining to PHI.

- **Phlebotomy:** Risks associated with blood draws will be minimized by proper blood drawing practices performed by a trained MD, RN, or phlebotomist. Universal precautions and sterile technique will be used. Participants will be monitored for adverse effects and counseled about the small risk of infection. In addition, the total amount of sample collection will be determined for each participant and reflect adaptations from several national guidelines, including the NIH Clinical Center. We will exclude any person with a hemoglobin <10 g/dL.

- **Fasting/MMTT:** We will observe for signs/symptoms associated with hypoglycemia during all fasting study visits. Glucose levels will be monitored closely throughout the MMTT. Meals will be given to participants once fasting is no longer required for the study visit. If any participant has clinical signs/symptoms associated with fasting, the study visit will be stopped, and the participant will be given juice/food or any other intervention, as necessary.

- **<u>Radiation exposure</u>:** Pregnant/lactating women will be excluded from study participation. A urine pregnancy test will be performed prior to obtaining any imaging studies that are associated with radiation exposure. If a person is deemed to be at an increased risk of any kind due to the minimal radiation associated with these tests, he/she will not be enrolled in the study or will be terminated from study participation.

- **<u>Side effects of study drug</u>:** Our exclusion criteria protect certain vulnerable people from potential side effects associated with GLP-1RAs. We will exclude anyone with diabetes or renal dysfunction, a history of alcohol abuse, pancreatitis, thyroid cancer, or a family/personal history of medullary thyroid carcinoma or MEN type 2. We will also exclude anyone with excessive alcohol intake, an elevated lipase or liver enzymes, and active gastrointestinal symptoms, as described in the inclusion/exclusion criteria. Pregnancy and lactation are exclusionary. Once enrolled in the study, participants will be monitored very closely for any side effects of the study drug and adverse events. Participants will undergo an 8-week titration period prior to receiving the full 1 mg dose. This titration period will consist of 4 weeks of 0.25 mg dose followed by 4 weeks of 0.5 mg dose. Their blood will be measured for serial safety laboratory tests, including comprehensive metabolic panels, which will include creatinine, lipase, among others. We will also monitor for clinical signs and symptoms suggestive of any concerning conditions, such as pancreatitis, thyroid dysfunction, etc. We will educate participants of important signs/symptoms, and they will be instructed to contact a member of the study team (24 hours/day by pager) with any concerns. In terms of the theoretical increased risk of thyroid cancers, the FDA did not find it useful to routinely monitor thyroid tests, thyroid ultrasound, or calcitonin levels, as these may lead to unnecessary evaluations. Participants with any of sign/symptoms concerning for this diagnosis will have his/her study drug withheld until the diagnosis is ruled out. Otherwise, participants will be permanently taken off study drug. Our interventional portion of the study is short (32 weeks only), and we will use an FDA-approved dose of semaglutide.

- **<u>Adverse events</u>:** All adverse events will be recorded in case report forms and graded per NIH guidelines. All >Grade 1 adverse events deemed possibly-, probably- or definitely-related to study participation will be reported to the local IRBs yearly and to the DSMB at the next meeting and will be presented in scientific publications. All >Grade 2 adverse events deemed to be possibly-, probably- or definitely-related to study participation will be reported to both institutions, the data safety and monitoring board (DSMB), NIH, and FDA within one week in writing. Any adverse event that requires medical attention will be addressed locally by the PI, and the participant will be referred to their PCP or emergency department, if deemed medically necessary.

- **<u>Incidental findings</u>:** Any incidental finding deemed clinically-relevant will be discussed with the participant, so that the participant can obtain medical treatment from his/her PCP or local emergency department. With permission from the participant, any relevant information will be sent to his/her PCP or emergency department, and the PI will contact the provider directly.

- **<u>Additional protection against risks</u>:** Participation of human participants in research is under the jurisdiction of federal regulations (45 CFR 46 and 21 CFR 50 and 56). Investigators at both sites are granted the privilege of working with human participants under normal assurance to the government that such research complies with regulations protecting human participants. Both universities have a federal-wide assurance for research with human participants and are in compliance with federal policy governing use of human participants. All human participant protocols are reviewed through the site's respective academic Institutional Review Boards (IRB) process that have been accredited by the

Association for Accreditation of Human Research Protection Programs (AAHRPP). All people involved have completed the University of Miami computer-based CITI Human Participants Research Education Course, which requires periodic re-certification. All research activity, informed consents and continuing reviews will be reviewed by the appropriate IRBs, in compliance with 45CFR46 before the research is started and continuing review will occur annually. The research staff will ensure that all information needed for continuing review is at the IRB in accordance with IRB requirements. After the study is completed, all data will be kept according to NIH and FDA regulations. All the procedures will be performed by the PI, trained study coordinators or study nurse. A data safety and monitoring board (DSMB) has been created to review interim data and oversee adverse events.

3 OBJECTIVES AND ENDPOINTS

OBJECTIVES	ENDPOINTS	JUSTIFICATION FOR ENDPOINTS
Primary		
To assess the effects of semaglutide on visceral and ectopic fat in HIV-infected participants with lipohypertrophy after 32 weeks of treatment	-Visceral and subcutaneous adipose tissue (by abdominal CT scan)<br><br>-Total, limb, trunk fat (by DXA scans)<br><br>-Pericardial fat (measured by non-contrast chest CT)	-DXA scans were previously used to assess longitudinal changes in fat after ART initiation or after interventions for lipodystrophy.[4,36,123,124] We will use the same standardized protocol on all study participants and each participant will have longitudinal measurements obtained on the same machine. DXA scans from both sites will be read at Case by a single radiologist, blinded to study arms and study information.<br><br>-The effect of GLP-1RA treatment on pericardial fat in HIV is unknown, as are the relationships between changes in pericardial fat and changes in insulin resistance and VAT. Thus, pericardial fat measurements will yield novel and informative data.
Secondary		
To examine the effects of semaglutide on lean body mass, inflammation, immune activation, cardiovascular disease risk, gut integrity, and gut hormones in HIV-infected individuals with lipohypertrophy after 32 weeks of treatment.	-Markers of monocyte activation: sCD14 and sCD163<br><br>-Markers of systemic inflammation: hsCRP, IL-6, sTNFR-1, sTNRF-II<br><br>-Gut integrity and microbial translocation will be assessed by I-FABP, zonulin-1, LPS	-The selected markers of monocyte activation and systemic inflammation have been previously shown to be associated with co-morbidities in HIV[38,39,136] and are relevant to adipose tissue, insulin resistance, and CVD.<br><br>-Pulse wave velocity is a non-invasive measure of arterial stiffness, and was shown to be associated with CVD events.[97]

OBJECTIVES	ENDPOINTS	JUSTIFICATION FOR ENDPOINTS
	-Markers of endothelial function: pulse wave velocity and EndoPat	-EndoPat, is non-invasive, reproducible, and strongly correlates with coronary endothelial function (r=0.79; P<0.001).[14] Endothelial dysfunction predicts atherosclerosis,[99,100]
Tertiary/Exploratory		
To elucidate potential mechanisms by which semaglutide affects visceral and ectopic fat.	-Adipokines measures (leptin, adiponectin, resistin, and visfatin)<br><br>-Natriuretic peptides (BNP and ANP)	-Adipokines and natriuretic peptides will be measured to help elucidate the potential mechanism by which GLP-1 may affect metabolic endpoints.

4 STUDY DESIGN

4.1 OVERALL DESIGN

The main objective of this study is to assess the potential beneficial effect of GLP1-RA on visceral and ectopic fat accumulation, insulin resistance, inflammatory markers and the downstream effect on cardiovascular disease risk assessment in HIV-infected individuals.

This study is a phase IIb, randomized, double-blinded, placebo-controlled clinical trial of the GLP1RA, semaglutide. The duration of the study will be 56 weeks. Patients will be recruited from 1 site (Cleveland, OH).

A population of HIV-infected individuals with lipohypertrophy will be studied. The interventional phase will last 32 weeks, followed by a 24-week observational phase to assess the sustainability of the intervention.

Patients will be randomized equally to one of 2 treatment arms:
1) Semaglutide 1.0 mg, given by weekly subcutaneous (SC) injection, after the initial 8-week titration.
2) Matching placebo given by weekly SC injection

Randomization will be performed at week 0. Those randomized to active semaglutide will be given 0.25 mg SC weekly for 4 weeks then 0.5 mg SC weekly for 4 weeks then start the 1 mg SC weekly until week 32. Those randomized to placebo will receive the equivalent titrated placebo for the first 8 weeks.

Study drugs will be provided free of charge for study participants. Antiretroviral therapy will not be provided by the study. The decision for antiretroviral therapy changes or discontinuation will be left to the primary HIV provider, but these changes will be documented in the study chart and will be accounted for during the analyses.

4.2 JUSTIFICATION FOR DOSE

We have selected once-weekly semaglutide 1.0 mg for this current study, as its properties and pharmacological profile make it, arguably, the most suited GLP-1RA to treat lipohypertrophy and cardiometabolic abnormalities in HIV.[47-49]

We chose a weekly SC route of administration instead of a daily regimen, which is likely to increase adherence and retention in HIV-infected individuals who are not accustomed to regularly receiving injections; it is the ideal agent to use in this current study.

A dose of 1 mg is the FDA-approved dose, and this dose showed superiority in reduction of body weight and glycemic control when compared to 0.5 mg dosage in SUSTAIN 1-6 trials. However, to adhere to the package insert and the need to improve tolerability and decrease the potential for nausea and vomiting, we will titrate the drug over an 8-week period.

4.3 END OF STUDY DEFINITION

A participant is considered to have completed the study if he or she has completed all study visits, including the last visit or the last scheduled procedure shown in the Schedule of Activities (SoA), Section 1.3.

The end of the study is defined as completion of the last visit or procedure shown in the SoA in the trial globally.

5 STUDY POPULATION

5.1 INCLUSION CRITERIA

To be eligible to participate in this study, participants must meet all of the following criteria:

1. Male or female, aged ≥18 years.

2. HIV-1 infection as documented by any licensed ELISA test kit and confirmed by Western blot at any time prior to study entry. HIV-1 culture, HIV-1 antigen, plasma HIV-1 RNA, or a second antibody test by a method other than ELISA is acceptable as an alternative confirmatory test.

3. Body mass index ≥25 kg/m^2.

4. Waist circumference and waist-to-hip ratio >95 cm and >0.94 cm, respectively, for men, and >94 cm and >0.88 cm, respectively, for women occurring in the context of HIV treatment.

5. Subjective evidence of increased abdominal girth occurring after initiation of HIV treatment.

6. HIV-1 RNA <400 copies/mL for ≥6 months.

7. Receiving a stable antiretroviral regimen for at least the last 12 weeks prior to study entry with cumulative duration of 1 year of treatment at the time of study entry.

8. Provision of signed and dated informed consent form and is capable of reading and comprehending the informed consent.

9. Stated willingness to comply with all study procedures and availability for the duration of the study.

10. All women of child-bearing potential (WOCBP) must have a negative serum or urine pregnancy test (minimum sensitivity 25 IU/L or equivalent units of HCG) within 72 hours prior to start of study medication. WOCBP is defined as any female who has experienced menarche and who has not undergone successful surgical sterilization (hysterectomy, bilateral tubal ligation, or bilateral oophorectomy), who is not postmenopausal (defined as amenorrhea 12 consecutive months), or is on hormone replacement therapy (HRT) with documented plasma follicle-stimulating hormone level 35 mIU/mL. Women who are using oral, implanted, or injectable contraceptive hormones or mechanical products such as an intrauterine device or barrier methods (diaphragm, condoms, spermicides) to prevent pregnancy or practicing abstinence or where partner is sterile (e.g., vasectomy), should be considered of child-bearing potential.

11. Female subjects who are not of reproductive potential (have reached menopause or undergone hysterectomy, bilateral oophorectomy or tubal ligation) or whose male partner has undergone successful vasectomy with resulting azoospermia or has azoospermia for any other reason, are eligible without requiring the use of contraception. Patient-reported history of menopause, sterilization, and azoospermia is considered acceptable documentation.

12. All subjects must not participate in a conception process (*e.g.* active attempt to become pregnant or to impregnate, sperm donation, in vitro fertilization), and if participating in sexual activity that could lead to pregnancy, the female subject/male partner must use condoms (male or female) in addition to one of the following forms of contraception while on study: either a spermicidal agent, diaphragm, cervical cap, IUD, or hormonal-based contraception.

13. Have no plans to alter antiretroviral therapy, or to undergo any weight loss program, formal exercise training or surgery during the study period, or initiate structured/strategic antiretroviral treatment interruptions.

5.2 EXCLUSION CRITERIA

Main exclusion criteria:

1. Known cardiovascular disease or diagnosed diabetes. If on metformin without a diabetes diagnoses metformin use has to be constant, uninterrupted for 6 months prior to entry.

2. Any active or chronic uncontrolled inflammatory condition, infection or cancer.

3. Women who are pregnant or breastfeeding.

4. Women with a positive pregnancy test on enrollment or prior to study drug administration.

5. A clinically-relevant illness within 14 days prior to study entry not explicitly excluded by the protocol, a physical or psychiatric disability, or a laboratory abnormality that might place the subject at increased risk by being exposed to the medications in this study or which might confound the interpretation of this investigation.

6. Active gastrointestinal symptom Grade >1 within the last month.

7. Regular use of immunomodulators/agents which could impact inflammation. Regular use of NSAIDS allowed if constant, uninterrupted for 6 months and no plans to alter. Statin use must also be constant, uninterrupted for 6 months prior to study entry. Thyroid medication allowed unless diagnosed with uncontrolled thyroid disease.

8. Inability to communicate effectively with study personnel.

9. Use of megestrol acetate, testosterone, or any steroid use beyond normal amounts found in the body within 6 months of study, or intend to start.

10. Glomerular filtration rate <50 cc/min/1.73 m^2.

11. Hemoglobin <10 g/dL.

12. Elevated lipase level >1.5 upper limit of normal

13. AST AND ALT >2.5x upper limit of normal.

14. Use of growth hormone or growth hormone-releasing hormone in the last year, or intent to start.

15. History of excessive alcohol use (on average 2 or more drinks a day), pancreatitis, thyroid cancer, or a diagnosis of multiple endocrine neoplasia (MEN) syndrome type 2.

16. Weight 450 lbs. or higher

5.3 LIFESTYLE CONSIDERATIONS

Individuals should have no plans to undergo any weight-loss program, formal exercise training or bariatric surgery during the study period. HIV-infected participants should additionally have no intention to initiate structured/strategic antiretroviral treatment interruptions.

For specific study visits (for the entry visit and week 32 of the interventional phase, and for week 56 of the observational phase), participants will be asked to be fasting for ≥12 hours prior to the study visit (as defined by no food or drink, except for plain water and medications). Participants will also be asked to be free of tobacco and caffeine for these visits, for at least 4 hours before PWV measurements.

5.4 SCREEN FAILURES

Screen failures are defined as participants who consent to participate in the clinical trial but are not subsequently randomly assigned to the study intervention or entered in the study. A minimal set of screen failure information is required to ensure transparent reporting of screen failure participants, to meet the Consolidated Standards of Reporting Trials (CONSORT) publishing requirements and to respond to queries from regulatory authorities. Minimal information includes demography, screen failure details, eligibility criteria, and any serious adverse event (SAE).

Patients who do not meet the criteria for participation in the trial due to modifiable factors (examples: active gastrointestinal symptoms >Grade 1 within the month prior to enrollment, pregnancy, lactation) might be rescreened with resolution of the specific factor/condition.

5.5 STRATEGIES FOR RECRUITMENT AND RETENTION

Recruitment:

Participants will be enrolled at the **Cleveland site**. Sources of study participants include: **1)** the 1,180 established patients currently followed in the HIV clinic at Case Western Reserve University (25% women, 40% African American, 3% Hispanic); **2)** patients seen at the HIV Metabolic Center, led by Dr. McComsey, a referral center established in 2004 that serves the entire northeastern portion of Ohio for evaluation and treatment of HIV-related metabolic complications. People are referred by their HIV providers or are self-referred for HIV-related metabolic concerns or for interest in participating in our metabolic clinical trials. The most common reason for referral has been, and continues to be, lipodystrophy. Each person undergoes a full metabolic assessment, including anthropometrics. The majority have failed attempts at diet and exercise, and among the last 82 patients seen for lipohypertrophy, only one has ever been treated with tesamorelin; **3)** Dr. Corrilynn Hileman, the Clinical Director of the HIV clinic at MetroHealth Medical Center (a Case affiliate and located within short distance geographically to Case) and close collaborator with both PIs, will identity and refer potential participants to Case. MetroHealth currently provides HIV care to 1615 adults (23% females, 52% African American, and 13% Hispanic).

Recruitment will be conducted like prior studies conducted by the PIs and will be maximized through a combination of education and outreach. Clinic recruitment methods will include: **1)** IRB-approved recruitment flyer in the clinics; **2)** study staff will approach patients during their routine medical visits after approval of their health care provider; and **3)** case manager/physician referrals.

Inclusion of Women: The enrollment of females with HIV in cardiometabolic studies is of great importance. There is a remarkable paucity of data regarding females living with HIV and ART-associated cardiovascular and metabolic complications. In addition, female gender has been identified as a risk factor for some metabolic toxicities, including lipodystrophy.

At the Case Western Reserve University site, the AIDS Clinical Trials Unit (ACTU), part of the NIH-funded AIDS Clinical Trials Group (ACTG), has been successful in recruiting women into their clinical trials.

Specifically, in Dr. McComsey's trials through the Case HIV Metabolic Center during the past 7 years, she has enrolled a proportion of women of 40%, which is higher than the proportion of female patients in their clinic (~20%). In prior non-ACTG metabolic studies conducted by Dr. McComsey at Case that required invasive tissue biopsies, 29% (60/206) of enrolled participants were females.

Dr. McComsey's prior success in enrolling female participants was due, in part, to constant outreach activities performed by Dr. McComsey with regular conferences and educational sessions in women focus groups. Enhancement of women's knowledge about metabolic co-morbidities associated with HIV helped her tremendously with enrollment in prior trials. Indeed, the study team will continue these efforts to improve women's knowledge about HIV, and, in particular, about metabolic and cardiovascular complications associated with HIV and HIV treatment.

Inclusion of Minorities: Case has been successful in enrolling minorities in clinical trials due to extensive outreach efforts geared toward local physicians in general and those treating people with HIV, AIDS service organizations, people live with HIV and those at risk for HIV, and the general public. During the past 6 years, the ACTU has enrolled in their trials a proportion of racial and ethnic minorities proportional to the population seen in their clinic and in Cuyahoga County (40% and 3%, respectively), where the clinic is located. Dr. McComsey's studies have had enormous success in enrolling racial minorities. For example, in a previous clinical trial of rosuvastatin in people with HIV, 101/147 (70%) of study participants were African American. Recruiting people from MetroHealth Medical Center, a Case affiliate, where the HIV clinic is comprised of 52% African Americans and 13% Hispanics will further enhance enrollment of minorities at the Case site.

Studying the effects of GLP-1RAs among African Americans is of high importance, as they are at an increased risk of obesity, diabetes, and cardiovascular disease compared to the general population. Therefore, MUSC has been added as a study site to increase enrollment of African-American participants. Her clinic is comprised of 73% African American patients with HIV, a proportion that is mirrored among the local clinics that have agreed to help refer eligible patients.

Inclusion of Children: Only adults 18 years of age and older will be enrolled in the proposed study, as there are limited data on the safety of GLP-1 receptor agonists in children. In addition, given the inclusion/exclusion criteria outlined in this proposal, few children would qualify for the study.

Illiterate Subjects: Subjects with all levels of literacy will be eligible for this study. The consent document will be read to those volunteers with less than an 8th grade equivalent level of literacy. Subsequently, the informed consent will be signed by the volunteer making their mark in the signature section in order to document their understanding. A witness will be present to confirm the consent process has taken place. Both the witness and person conducting the consent process will sign and date the consent. The investigator obtaining consent will ask each subject to reiterate what will be required from them, risks and benefits, and their rights as a participant in order to ensure their full understanding of the study.

Non-English-Speaking Subjects: Subjects who do not understand or speak English will also be eligible for this study. The consent form will be read to those non-English speaking study candidates in their primary language by a translator. A witness (who speaks English and the study subject's language) will be present to confirm the consent process has taken place. Both the witness and person conducting the consent process will sign and date the consent. The investigator obtaining consent will ask the study candidate via the translator to reiterate what will be required from him/her, risks and benefits, and his/her rights as a participant to ensure their full understanding of the study.

Employees: Anyone with an employment or academic relationship to the Principal Investigator will be informed that their participation in a study, or refusal to do so, will in no way influence their employment, or subsequent recommendations. Employees will never be made to feel that their job, promotion, salary, or status in any way depends on participation in research studies.

Retention:

The PIs and study staff will utilize and expand upon previously successful efforts for study retention and adherence strategies including: (1) excluding people who anticipate transportation or feasibility issues during the study period; (2) financial compensation for time and travel ($25 for the following study visits: screening, week 9, week 14, week 20, week 26 and week 31; $50 for the following study visits: -1, Week 0 and week 32; $75 for week 56. 3) paid parking for study visits; 4) food/drink during fasting study visits; 5) reminder phone calls the day prior to scheduled appointments and additional calls to reschedule those who miss appointments; 6) additional use of participant's preferred mode of communication including text messages and email for research visit scheduling, reminders, and rescheduling for missed appointments. A careful selection of study participants based on an established track record of kept clinic appointments, viral suppression, and stable ART will further improve study retention and adherence. In several prior 48-96-week trials requiring extensive metabolic assessments (*e.g.* tissue biopsies), our retention was >90%; thus, we do not anticipate any issues with study retention.

6 STUDY INTERVENTION

6.1 STUDY INTERVENTION(S) ADMINISTRATION

6.1.1 STUDY INTERVENTION DESCRIPTION

According to the drug package and FDA report (Semaglutide s.c. OW NDA 209637):

Semaglutide is a selective GLP-1 receptor agonist (GLP-1 RA) with a long plasma half-life suitable for once-weekly dosing. The long half-life was obtained by applying the fatty acid acylation technology that provides specific high-affinity albumin binding. Furthermore, semaglutide has full stability against DPP-4 degradation. The inherent long half-life together with a low molecular weight of semaglutide is believed to ensure optimal efficacy. Semaglutide exhibits GLP-1 receptor-mediated effects, leading to lowering of glucose and decreased appetite through physiologically relevant mechanisms. As a result, semaglutide provides strong glycemic control and weight loss. In addition, the cardiovascular safety of semaglutide has been confirmed. The mechanism of action of semaglutide was characterized in extensive nonclinical and clinical studies.

Ozempic® (semaglutide) injection 1 mg is indicated as an adjunct to diet and exercise to improve glycemic control in adults with type 2 diabetes mellitus and received FDA approval in December 2017:

- Ozempic® is not recommended as a first-line therapy for patients who have inadequate glycemic control on diet and exercise because of the uncertain relevance of rodent C-cell tumor findings to humans.
- Ozempic® has not been studied in patients with a history of pancreatitis. Consider other antidiabetic therapies in patients with a history of pancreatitis.

- Ozempic® is not a substitute for insulin. Ozempic® is not indicated for use in patients with type 1 diabetes mellitus or for the treatment of patients with diabetic ketoacidosis.
- It is not known if OZEMPIC® is safe and effective for use in children under 18 years of age.

6.1.2 DOSING AND ADMINISTRATION

After an initial 8-week period of titration, semaglutide 1.0 mg will be administered by SC injection into the abdomen, thigh, or upper arm at any time of day on the same day each week, with or without food. If changing the day of administration is necessary, allow at least 48 hours between two doses.

Rotate injection sites weekly if injecting in the same area of the body. Injection will not be mixed with other products (administer as separate injections).

Patient will be instructed to avoid adjacent injections if administering other agents in the same area of the body.

6.2 PREPARATION/HANDLING/STORAGE/ACCOUNTABILITY

6.2.1 ACQUISITION AND ACCOUNTABILITY

The study agent, semaglutide, is an FDA-approved drug indicated as an adjunct to diet and exercise to improve glycemic control in adults with type 2 diabetes mellitus. It is readily available from commercial/outpatient pharmacies and pharmacies affiliated with hospitals and tertiary-care academic centers. The investigational pharmacies (specialized pharmacies specifically designed for clinical trials) at the respective enrollment sites will oversee the acquisition, drug accountability, and dispensing of the study agent in accordance with local and federal regulations. The co-PIs (both MDs) and the study team's RNs at the respective sites will administer the medication to the study participants. Because semaglutide has not been studied in people with HV-associated lipohypertrophy, the study team will apply for an IND from the FDA for this study protocol to help ensure participant safety.

6.2.2 FORMULATION, APPEARANCE, PACKAGING, AND LABELING

According to the Ozempic® label:

NDC: 00169413602
Generic name: Semaglutide
Labeler: NOVO NORVODISK
Brand name: Ozempic
Color List: colorless
Clarity: clear
Strength Field Collection: 1 mg per dose (2 mg/1.5 mL)
Dosage Form: Solution Pen-injector
Discontinued: no
Route List: subcutaneous

Contains List: phenol; propylene glycol
Bioequivalency Rating: NR
Rx/OTC: Rx (single source)

6.2.3 PRODUCT STORAGE AND STABILITY

The investigational pharmacies at the respective sites will refrigerate the interventional drug according to the manufacturer's recommendations at 36°F to 46°F (2°C to 8°C) until dispensed.

6.2.4 PREPARATION

Investigational pharmacies will dispense study drug or matching placebo (saline) in a pre-filled syringe which will be administered by study staff.

6.3 MEASURES TO MINIMIZE BIAS: RANDOMIZATION AND BLINDING

Participants will be randomized 1:1 to receive semaglutide by SC injection once weekly, or matching placebo, for 32 weeks. The initial 8 weeks will be the titration phase (0.25 mg SC weekly for 4 weeks then 0.5 mg SC weekly for 4 weeks, then 1 mg SC weekly until week 32). The study statistician will generate randomization sheets which will be sent directly to the investigational pharmacists. After being provided to the pharmacists, the randomization schedule will be maintained in a locked, secured location at the pharmacy. Only site pharmacists can access the randomization schedule at the time of the intervention assignment. On the day of the visit, investigational pharmacies will dispense study drug or matching placebo (saline) in identical-appearing, pre-filled syringes that do not contain any indication of study arm.

Al participants, study staff, key personnel, consultants and DMSB members will be blinded to treatment allocation. All participants will receive identical information regarding potential side effects adverse events. Unblinding of treatment allocation will occur only if warranted to optimize management of an adverse event or for other safety reasons. In these specific circumstances, the investigational pharmacist will be responsible for breaking the blind to the principal investigator. This will occur in a private location to ensure the rest of the team remains blinded.

All statistical analyses will also be done in a blinded fashion. Data will be exported to the statistician as arm A versus arm B by the pharmacist to preserve blinding.

6.4 STUDY INTERVENTION COMPLIANCE

Study drug (active or placebo) will be injected SC by the research staff once weekly, which will help ensure adherence and improve retention, as study participants will not have to worry about self-injecting.

6.5 CONCOMITANT THERAPY

For this protocol, a prescription medication is defined as a medication that can be prescribed only by a properly authorized/licensed clinician. Medications that will be reported are prescription and over-the-counter medications and supplements.

Data regarding concomitant medications will be collected at the screening visit. Additionally, at all study visits, patients will be asked to report any changes to their medication list.

6.5.1 RESCUE MEDICINE

Not applicable.

7 STUDY INTERVENTION DISCONTINUATION AND PARTICIPANT DISCONTINUATION/WITHDRAWAL

7.1 DISCONTINUATION OF STUDY INTERVENTION

For any study participant who develops a ≥Grade 3 toxicity felt to be possibly- or probably-related to study drug, the study drug will be interrupted, and the participant promptly evaluated. If the ≥Grade 3 toxicity is deemed not to be related to the study drug, then the drug can be restarted (after symptoms resolve) with very close monitoring of recurrent symptoms. If a participant develops possible pancreatitis or thyroid cancer symptoms during the study, he/she will be promptly evaluated, and study drug withheld. If the diagnosis of pancreatitis or thyroid cancer is proven or likely, study drug will be permanently discontinued and the participant will be followed on-study, off-study drug. The investigators will also adhere to any recommendations made by the DSMB. In addition, if during the study, a participant develops diabetes requiring hypoglycemic agents, the study drug will be withheld, and the participant will be followed off study drug. At any point, if deemed medically-necessary, the participant's study arm (placebo and active drug) will be unblinded, and the participant will be followed off study drug.

7.2 PARTICIPANT DISCONTINUATION/WITHDRAWAL FROM THE STUDY

<u>CRITERIA FOR PERMANENT STUDY DISCONTINUATION</u>:

- Request by the participant to withdraw.
- At the discretion of the FDA, IRB, DSMB, or NIH.
- Pregnancy or breastfeeding.
- Participant judged by the investigator to be at significant risk of failing to comply with the provisions of the protocol.
- Request of the primary care physician if he/she thinks the study is no longer in the best interest of the participant.

7.3 LOST TO FOLLOW-UP

A participant will be considered lost to follow-up if he or she fails to return for scheduled visits and is unable to be contacted by the research staff.

The following actions must be taken if a participant fails to return to the clinic for a required study visit:

1. The research staff will attempt to contact the participant and reschedule the missed visit for another day during the same week and counsel the participant on the importance of maintaining the assigned visit schedule and ascertain if the participant wishes to and/or should continue in the study.
2. Before a participant is deemed lost to follow-up, the investigator or designee will make every effort to regain contact with the participant (where possible, 3 telephone calls). Contact (and attempts to contact) will be documented in the participant's medical record or study file.
3. Should the participant continue to be unreachable, he or she will be considered to have withdrawn from the study with a primary reason of lost to follow-up.
    a. Staff will ask the participant to return to clinic for a premature study discontinuation visit where week 56 evaluations will be followed.
    b.

8 STUDY ASSESSMENTS AND PROCEDURES

8.1 EFFICACY ASSESSMENTS

Screening and entry:

Potential participants will be asked to visit the clinic at least once to be screened and ensure that they meet the requirements for entry into the study. All screening evaluations to determine eligibility must be completed within 30 days prior to study entry. If a potential participant shows up ill with any acute symptom, the visit will be postponed until all symptoms have resolved for at least 7 days. Screening may take place on the same day as a normally-scheduled clinic appointment, if the person has been fasting for at least 12 hours. Before any tests or information can be obtained as part of this study, potential participants must undergo the informed consent process including providing written informed consent. The site PI or member of the study team will conduct the informed consent process and complete a checklist indicating date and time the consent was signed, who was present, version date, and if a copy was provided to participant that will be filed in the participant's chart.

As part of the screening process, participants will undergo:

- A series of health-related questions to determine their eligibility.
- A review of any applicable medical records and laboratory results as related to the inclusion/exclusion criteria.
- A targeted physical examination, including height, weight, blood pressure, and anthropometric measurements.
- A blood draw for hematology/chemistries as outlined in the inclusion/exclusion criteria.
- An HIV test (when applicable).
- For woman of reproductive potential, a urine sample will be taken for a pregnancy test.

If a participant does not meet inclusion criteria, the reason for ineligibility will be noted in the participant's study chart.

Lead-in phase: After passing screening, eligible participants will enter the 4-week lead-in phase, and they will receive standardized dietary and physical activity advice from the clinic dietician. It is very unlikely that the dietary advice will generate any significant change in lifestyle habits as patients already receive this advice as part of standard of care. The 4-week run-in phase (with weekly visits) will help assess the stability of the lifestyle habits and anthropometrics, and, importantly, will enhance our chances to enroll

the most committed participants with the highest likelihood to adhere to study requirements and weekly visits.

During the weekly visits, participants will undergo:

- A targeted physical examination, including weight, blood pressure, and anthropometric measurements.
- Detailed dietary and physical activity questionnaire.
- At visit -1, participants will also undergo a test to measure gut hormone levels (4-hour mixed meal tolerance test). For this visit, subjects will be compensated $25.00.

Interventional phase: The study statistician will generate randomization sheets which will be sent directly to the investigational pharmacists, and the respective sites' investigational pharmacy will oversee randomization and dispensing of study drugs. On the first study visit of the investigational phase, the investigational pharmacy will dispense study drugs or matching placebo (saline) in a pre-filled, blinded syringe. Study drug (active or placebo) will then be injected subcutaneously by the research staff once weekly, in the abdomen, thigh or upper arm, at the discretion of the study participants in concurrence with the staff and independent of meals. Participants will be seen once weekly for injections and close monitoring.

The interventional phase will consist of two phases: titration phase and full-dose phase.

Titration phase: Participants will receive at W1, W2, W3, and W4 0.25 mg of semaglutide and then will be increased to 0.5 mg semaglutide at W5, W6, W7 and W8. This titration phase allows potential side effects to be monitored and participants to gradually increase to the 1.0 mg dose. Participants will receive a bus pass or parking voucher for this visit.

After the 8-week titration phase, participants will start the 24-week full dose phase where semaglutide will be administered weekly at 1 mg SC dosing.

At entry (week 0), week 14, 20, 26 and 32 study visits, participants will undergo a comprehensive clinical assessment with performance of safety laboratories (chemistries with lipase, hematology, and pregnancy test for women). HIV-1 RNA and CD4/CD8 will also be measured as part of routine care every 12 weeks. Fasting (≥12 hours) will be required for entry and weeks 20 and 32. A period of at least 4 hours of smoking and tobacco abstinence will be also required before study visits at entry and 32 weeks.

Observational phase: After the 32-week interventional phase, participants will continue on to the post-treatment observational phase, off study drug, and they will be seen at week 56 for the last study evaluation, as outlined in the table of study procedures. This visit will assess the durability of the intervention, and it will assess body composition, inflammation markers and cardiovascular outcomes. This visit will also be performed in a fasting state (≥12 hours) and will require a period of at least 4 hours of smoking and tobacco abstinence.

Sources of materials: Sources of materials obtained from the participants will include self-reported data from questionnaires and interviews with study staff, medical records, physical examination/body measurements, non-invasive/non-radiative cardiovascular assessments, DXA scan, blood, urine, and stool.

DETAILED STUDY ASSESSMENTS AND PROCEDURES (timing of different procedures presented in Section 1.3.1)

- **Detailed dietary assessments** will be obtained by study staff, overseen by a registered dietician, and analyzed using the Nutrition Data System for Research (NDS-R) software version 2005.

- **Body image questionnaires** will assess changes in the amount of fat at specific body sites, adopted from the Study of Fat Redistribution and Metabolic Change in HIV Infection (FRAM).[121]

- **Physical activity status** will be assessed based on standardized questionnaires.[122]

- **Anthropometric measurements** including weight, height, waist and hip circumferences will be obtained using standardized techniques.

- **Whole-body DXA scans** will be used to assess changes in body composition, including measurements of total, limb, and trunk fat, and lean mass. We have used DXA to assess longitudinal changes in fat after ART initiation or after interventions for lipodystrophy.[4,36,123,124] We will use the same standardized protocol on all study participants and each participant will have longitudinal measurements obtained on the same machine. DXA scans from both sites will be read at Case by a single radiologist, the co-I, Dr. AnsariGilani, blinded to study arms and study information**.**

Abdominal CT (for body composition and liver fat) will be used to assess TAT, VAT, and SAT, as previously described.[7]

- Non-contrast CT of the abdomen will be obtained (Helical CT 3mm slice thickness with 3mm scan increment extending from the dome of the diaphragm through the symphysis pubis). Liver and spleen attenuation will be measured by averaging three circular regions of interest with an area of at least 2 cm2 within the parenchyma of each organ on different axial slices, taking care to avoid vessels and bile ducts. The ratio of liver to spleen attenuation will then be calculated. In healthy individuals, the mean CT radiodensity for the liver is consistently higher than that for the spleen. As the fatty liver has lower mean CT radiodensity than the spleen, the liver-to-spleen attenuation ratio is used as an index of liver fat, with increases in the ratio indicating reduced hepatic steatosis. We will also assess changes in serum liver transaminases to complement the CT results.
- Also, a single slice of the abdomen at the level of L4/L5 disc space will be selected and will be used for the measurement of VAT and SAT.
- Dr. Ansari-Gilani, a graduate from a Body Fellowship from Washington University, will oversee standardization of the images at both sites with the use of phantoms, will be the central reader for all scans, and will be blinded to clinical and treatment characteristics. Dr. McComsey has extensively used both DXA and CT scans in prior HIV studies.

- **Pericardial fat** content and density will be measured by non-contrast, chest CT scan, as previously described.[18,46] The effect of GLP-1RA treatment on pericardial fat in HIV is unknown, as are the relationships between changes in pericardial fat and changes in insulin resistance and VAT. Thus, pericardial fat measurements will add novel and informative data.
- A noncontrast electrocardiogram-gated low-dose cardiac computed tomography (CT) for calcium scoring will be done in all patients. Images will be used to measure the pericardial fat by single reader (Dr. Ansari-Gilani) who will be blinded to randomization arm and subject characteristics.

- **Resting energy expenditure** will be assessed using handheld, portable, indirect calorimetry (Medgem by HealtheTech, now part of MicroLife Corporation). Participants will be instructed to come fasting from food (≥12h) and tobacco-/caffeine-free (>4h). A single-use mouthpiece and nose clip will be used, and participants will be instructed to breathe through their mouths into the self-calibrating Medgem device. Oxygen consumption (VO_2) will be measured for 10 minutes to ensure achievement of a steady state, and energy expenditure will be calculated by the Weir equation.[125] We have previously shown that REE is elevated in ART-naïve women with HIV and continues to be elevated even with effective ART and virologic suppression,[126] suggesting an effect of HIV infection itself. Thus, this measurement will be particularly informative in the context of GLP-1RA treatment.

- **Natriuretic peptides (BNP and ANP)** will be measured to help elucidate a potential mechanism by which GLP-1RAs may affect the metabolic endpoints measured in this proposal. ANP decreases blood pressure and cardiac hypertrophy, while BNP acts to reduce ventricular fibrosis. We have shown in a previous HIV study that BNP is independently associated with several CVD markers.[127]

- **Adipokines** (leptin, adiponectin, resistin and visfatin) will be measured as previously described[128] to inform us on the potential mechanism by which GLP-1RAs may affect VAT and inflammation markers.

- **Gut hormones** will be measured from blood and collected during a 4-hour, mixed-meal tolerance test (MMTT). After fasting (≥12h), participants will be asked to drink an Ensure Plus (vanilla-flavored) nutritional supplement (5mL/kg of participant's screening body weight). If participants have a milk intolerance this test will NOT be obtained due to ensure being a milk based product. Blood samples will be obtained via peripheral intravenous catheter 15 minutes before consumption, immediately prior to consumption, and then at 20, 40, 60, 90, 120, 180, and 240 minutes after consumption for measurement of glucose, insulin, GLP-1, GIP, glucagon, and C-peptide. Blood will be collected into tubes containing DPP4 inhibitor, EDTA and trasylol and centrifuged immediately in a refrigerated centrifuge. Plasma samples will be processed, aliquoted into several aliquots (gut hormones, due to breakdown in the thawing process, should be assayed in aliquots thawed just once), and immediately frozen. Blood will be batched and sent to Dr. Egan at the Diabetes Section of the National Institute of Aging for measurements of gut hormones, as previously described.[129,130] Blood glucose levels will also be measured and monitored in real-time using Nova Biomedical STATStrip glucometer. A 4-hour follow-up period is necessary, as incretin hormones may be elevated for ≥3.5 hours after a meal. Participants will be given a meal after the test. As previously discussed, this study will be the first to measure GLP-1 or GIP in the setting of HIV-associated lipohypertrophy. Like GLP-1, GIP is secreted in the gut in response to dietary fat and glucose and is involved in adipocyte metabolism. Specifically, it enhances insulin-stimulated incorporation of fatty acids into triglycerides, stimulates lipoprotein lipase activity, and modulates fatty acid synthesis.[131,132] GIP treatment has been shown to reduce obesity-induced adipose tissue inflammation and improve insulin sensitivity in mice.[133]

- **Monocyte immune activation (sCD14 and sCD163)** will be measured by ELISA (R&D Systems), which correlates with coronary calcifications,[113] non-calcified coronary plaques,[134] and arterial inflammation[135] in HIV and has been shown to decrease significantly as early as 18-24 weeks after a successful intervention.

- **Systemic inflammation** will be assessed by measuring select markers that we have previously shown are associated with co-morbidities in HIV[38,39,136] and are relevant to adipose tissue, insulin resistance, and CVD. hsCRP, IL-6, sTNFR-I, and sTNFR-II will be measured by ELISA (R&D Systems).

- **Standard lipoprotein profile and oxidized-LDL levels** will be measured in a fasting state by ELISA (Mercodia).[137,138]

- **Insulin resistance and glucose tolerance** will be assessed by measuring fasting insulin and glucose and then again 2 hours after administration of an oral glucose load of 75 grams, as previously described.[139] We will also calculate HOMA-IR, a reproducible index of insulin resistance.[140]

- **Gut integrity and microbial translocation** will be measured to explore whether the improvement in VAT associated with GLP-1RA treatment is independent of changes in gut barrier integrity. We will use ELISA to measure plasma levels of LPS (a marker of microbial translocation; R&D Systems), intestinal fatty acid binding protein (I-FABP; a marker of enterocyte damage; R&D Systems),[141] and zonulin-1 (a protein that modulate the permeability between cells of the digestive tract wall;[142] Immundiagnostik). In HIV-uninfected populations, increased zonulin-1 levels are found in conditions associated with heightened inflammation and had been linked to obesity, insulin resistance, and liver steatosis.[143,144] In HIV, increased levels of zonulin have been linked to increased mortality.[145]

- **Pulse wave velocity** is a non-invasive measure of arterial stiffness that uses SphygmoCor technology. PWV involves the detection of pulse vibrations with the use of a tonometry pen pressed against the skin[146,147] and is associated with CVD events.[97] Augmentation index, another measure of arterial stiffness that correlates with CVD risk,[98] will also be measured, normalized to a heart rate of 75. The investigators have used PWV in other studies,[148] and previous in-house data at both sites show excellent measurement error (CV 3-4%). Designated technicians will perform the PWV tests with SphygmoCor equipment (CPVH model, v.9 software).

- **Endothelial function (EndoPAT):** a non-invasive, user-independent technique using the FDA-approved EndoPAT2000 (Itamar Medical Ltd, Israel) provides a reliable and reproducible assessment of endothelial function that predicts atherosclerosis and CVD events. Using modified plethysmographic biosensors, the PAT signal is measured from the fingertip by measuring arterial pulsatile volume changes. A cuff is inflated around the upper arm to obstruct flow and released. The surge of blood flow causes endothelial dependent FMD, manifested as a reactive hyperemia. The EndoPAT® calculate the RHI, the ratio of digital pulse volume during reactive hyperemia and the baseline. In addition to the RHI, we will obtain peripheral Aix, adjusted for HR of 75, which will be compared to central Aix obtained with PWV. We have excellent track record in performing vascular studies, and carefully control for factors that could influence endoPAT; studies are performed at 24 C with the patient resting quietly. Subjects will be fasting, without smoking, caffeine, and exercise for >4 hours.

- **Skin AGE Measurements*:*** There has been evidence that diet effects advanced glycation end products (AGE) therefore Skin AGE measurements will be obtained. This study has designated the AGE Reader instrument (manufactured by Diagnoptics Technologies B.V. located in Groningen Netherlands) as having a "nonsignificant risk" to study participants. Some individuals are known to have, or are at risk for, photosensitivity reactions (e.g., sensitive to ultraviolet light, or taking medication known to cause photosensitivity). We will exclude from the study those potential participants who report having these risk factors. However, there is a negligible risk that these participants might not be known until they develop a reaction.

- **Additional plasma, PBMCs, serum, urine, and stool** will be collected and frozen for future measurements of cellular immune activation, CVD and metabolic markers, other inflammatory or oxidative factors. Optional genetic material will be stored.

- In extreme situations where the participant is unable to come to the clinic for a weekly injection and the participant's home is within reasonable driving distance from the clinic to allow the IP to be administered within the required 30 minutes of being prepared by the pharmacy, with the participant's consent, the study nurse and second study personnel will go to the participant's residence to administer the injection. Participants will be informed that study staff who are visiting the participant's residence for this purpose are legally required to report any witnessed or suspected elder or child abuse or other suspicious activities to the appropriate authorities if they observe them. In home injections will only be given if a participant is physically unable to come to the study clinic.

8.2 SAFETY AND OTHER ASSESSMENTS

Our exclusion criteria protect certain vulnerable people from potential side effects associated with GLP-1RAs. We will exclude anyone with diabetes or renal dysfunction, a history of alcohol abuse, pancreatitis, thyroid cancer, or a family/personal history of medullary thyroid carcinoma or MEN type 2. We will also exclude anyone with excessive alcohol intake, an elevated lipase or liver enzymes, active gastrointestinal symptoms, a hemoglobin <10 g/dL, as described in the inclusion/exclusion criteria. Pregnancy and lactation are exclusionary.

Once enrolled in the study, participants will be monitored very closely for any side effects of the study drug and adverse events. They will be seen weekly and safety monitoring will occur at each of these study visits including evaluations for clinical signs and symptoms suggestive of any concerning conditions, such as pancreatitis, thyroid dysfunction, etc. At the comprehensive study visits, participants will be monitored closely for sign/symptoms and any adverse events associated with the procedures (*e.g.* during and after blood draws, while fasting, etc.).

Participants' blood will be measured for serial safety laboratory tests at week 14, 20, 26, 32 and 56 study visits, including comprehensive metabolic panels, which will include liver function tests, creatinine, lipase, among others. Urine pregnancy tests will be done on females of reproductive potential as outlined in the schedule of activities. HIV-1 RNA and CD4/CD8 will also be measured as part of routine care every 12 weeks.

We will educate participants about important signs/symptoms, and they will be instructed to contact a member of the study team (24 hours/day by pager) with any concerns. Participants with any of sign/symptoms concerning for this diagnosis will have his/her study drug withheld until a diagnosis is made. Participants will be permanently taken off study drug when indicated, as outlined in Section 7.1.
Our interventional portion of the study is short (32 weeks only), and we will use an FDA-approved dose of semaglutide.

8.3 ADVERSE EVENTS AND SERIOUS ADVERSE EVENTS

8.3.1 DEFINITION OF ADVERSE EVENTS (AE)

Adverse event means any untoward medical occurrence associated with the use of an intervention in humans, whether or not considered intervention-related (21 CFR 312.32 (a)).

8.3.2 DEFINITION OF SERIOUS ADVERSE EVENTS (SAE)

An adverse event (AE) or suspected adverse reaction is considered "serious" if, in the view of either the investigator or sponsor, it results in any of the following outcomes: death, a life-threatening adverse event, inpatient hospitalization or prolongation of existing hospitalization, a persistent or significant incapacity or substantial disruption of the ability to conduct normal life functions, or a congenital anomaly/birth defect. Important medical events that may not result in death, be life-threatening, or require hospitalization may be considered serious when, based upon appropriate medical judgment, they may jeopardize the participant and may require medical or surgical intervention to prevent one of the outcomes listed in this definition. Examples of such medical events include allergic bronchospasm requiring intensive treatment in an emergency room or at home, blood dyscrasias or convulsions that do not result in inpatient hospitalization, or the development of drug dependency or drug abuse.

8.3.3 CLASSIFICATION OF AN ADVERSE EVENT

8.3.3.1 SEVERITY OF EVENT

The following guidelines will be used to describe severity of adverse events:

1. **Mild** – Events require minimal or no treatment and do not interfere with the participant's daily activities.
2. **Moderate** – Events result in a low level of inconvenience or concern with the therapeutic measures. Moderate events may cause some interference with functioning.
3. **Severe** – Events interrupt a participant's usual daily activity and may require systemic drug therapy or other treatment. Severe events are usually potentially life-threatening or incapacitating. Of note, the term "severe" does not necessarily equate to "serious".

8.3.3.2 RELATIONSHIP TO STUDY INTERVENTION

All adverse events (AEs) will have their relationship to study intervention assessed by the study staff who examines and evaluates the participant based on temporal relationship and his/her clinical judgment. The degree of certainty about causality will be graded using the categories below:

1. **Definitely Related** – There is clear evidence to suggest a causal relationship, and other possible contributing factors can be ruled out. The clinical event, including an abnormal laboratory test result, occurs in a plausible time relationship to study intervention administration and cannot be explained by concurrent disease or other drugs or chemicals. The response to withdrawal of the study intervention (dechallenge) should be clinically plausible. The event must be pharmacologically or phenomenologically definitive, with use of a satisfactory rechallenge procedure if necessary.

2. **Possibly Related** – There is evidence to suggest a causal relationship, and the influence of other factors is unlikely. The clinical event, including an abnormal laboratory test result, occurs within a reasonable time after administration of the study intervention, is unlikely to be attributed to concurrent disease or other drugs or chemicals, and follows a clinically reasonable response on withdrawal (dechallenge). Rechallenge information is not required to fulfill this definition. Or, there is some evidence to suggest a causal relationship (e.g., the event occurred within a reasonable time after administration of the trial medication). However, other factors may have contributed to the event (e.g., the participant's clinical condition, other concomitant events). Although an AE may rate only as "possibly related" soon after discovery, it can be flagged as requiring more information and later be upgraded to "definitely related" as appropriate.
3. **Not related** – A clinical event, including an abnormal laboratory test result, whose temporal relationship to study intervention administration makes a causal relationship improbable (e.g., the event did not occur within a reasonable time after administration of the study intervention) and in which other drugs or chemicals or underlying disease provides plausible explanations (e.g., the participant's clinical condition, other concomitant treatments). Or, the AE is completely independent of study intervention administration, and/or evidence exists that the event is definitely related to another etiology. [There must be an alternative, definitive etiology documented by the clinician.]

8.3.3.3 EXPECTEDNESS

The principal investigator will be responsible for determining whether an adverse event (AE) is expected or unexpected. An AE will be considered unexpected if the nature, severity, or frequency of the event is not consistent with the risk information previously described for the study intervention.

8.3.4 TIME PERIOD AND FREQUENCY FOR EVENT ASSESSMENT AND FOLLOW-UP

The occurrence of an adverse event (AE) or serious adverse event (SAE) may come to the attention of research staff during study visits and interviews of a study participant presenting for medical care, or upon review by a study monitor. Principal Investigators and study team will review all safety labs and clinical assessments on a continual basis. All adverse events will be recorded in case report forms and graded per NIH guidelines.

All AEs including local and systemic reactions not meeting the criteria for SAEs will be captured on the appropriate case report form (CRF). Information to be collected includes event description, time of onset, clinician's assessment of severity, relationship to study product (assessed by principal investigator), and time of resolution/stabilization of the event. All AEs occurring while on study will be documented appropriately regardless of relationship. All AEs will be followed to adequate resolution.

Any medical condition that is present at the time that the participant is screened will be considered as baseline and not reported as an AE. However, if the study participant's condition deteriorates at any time during the study, it will be recorded as an AE.

Changes in the severity of an AE will be documented to allow an assessment of the duration of the event at each level of severity to be performed. AEs characterized as intermittent require documentation of onset and duration of each episode.

Research staff will record all reportable events and inform directly the Principal Investigators with start dates occurring any time after informed consent is obtained until 7 (for non-serious AEs) or 30 days (for SAEs) after the last day of study participation. At each study visit, the investigator will inquire about the occurrence of AE/SAEs since the last visit. Events will be followed for outcome information until resolution or stabilization.

All laboratory results will be assessed by the study team. Abnormal laboratory values are defined as values outside the defined reference range and will be graded as per the NIH laboratory grading scale. Grade 1 and 2 laboratory values will be discussed with the PI within one week and any Grade >2 laboratory value will be discussed in real-time with the PI. When deemed clinically-necessary, the participant will be contacted and brought back for repeat testing within 1 week. Critical values will be repeated without delay when deemed clinically-necessary. The participant's primary provider will be notified within one week if the participant had given prior permission for release of medical information.

8.3.5 ADVERSE EVENT REPORTING

Principal Investigators and study team will review all safety labs and clinical assessments on a continual basis. All adverse events will be recorded in case report forms and graded per NIH guidelines. All >Grade 1 adverse events deemed possibly-, probably- or definitely-related to study participation will be reported to the local IRBs yearly and to the DSMB and will be presented in scientific publications. All >Grade 2 adverse events deemed to be possibly-, probably- or definitely-related to study participation will be reported to both institutions, the DSMB, NIH, and FDA within one week in writing. DSMB findings will be submitted to the local IRBs with the yearly continuing review (or ad hoc, when indicated) in a report prepared by the PIs and study staff and will include enrollment and drop-out rates, protocol deviations, participant symptoms, and review of clinical and laboratory results. The PIs will notify the NIH, FDA, and DSMB of any action recommended by the IRBs that may affect the conduct of the trial.

8.3.6 SERIOUS ADVERSE EVENT REPORTING

Principal Investigators and study team will review all safety labs and clinical assessments on a continual basis. All adverse events will be recorded in case report forms and graded per NIH guidelines. All serious adverse events deemed to be possibly-, probably- or definitely-related to study participation will be reported to both institutions, the DSMB, NIH, and FDA within one week in writing. DSMB findings will be submitted to the local IRBs with the yearly continuing review (or ad hoc, when indicated) in a report prepared by the PIs and study staff and will include enrollment and drop-out rates, protocol deviations, participant symptoms, and review of clinical and laboratory results. The PIs will notify the NIH, FDA, and DSMB of any action recommended by the IRBs that may affect the conduct of the trial.

8.3.7 REPORTING EVENTS TO PARTICIPANTS

The study team will contact a participant within one week with any clinical and laboratory result Grade 1-2 that is deemed clinically-relevant. Participants will be contacted immediately when deemed clinically-necessary. With permission from the participant, any relevant information will be sent to his/her PCP or emergency department, and the PI will contact the provider directly.

Any incidental finding deemed clinically-relevant will be discussed with the participant, so that the participant can obtain medical treatment from his/her PCP or local emergency department. With permission from the participant, any relevant information will be sent to his/her PCP or emergency department, and the PI will contact the provider directly.

If there are any adverse events or study findings that may impact the safety of participants or change their willingness to participate in the trial, participants will be notified within one week.

8.3.8 EVENTS OF SPECIAL INTEREST

Not applicable.

8.3.9 REPORTING OF PREGNANCY

If a female participant has a confirmed pregnancy, she will immediately be taken off study immediately. This event will be reported to the DSMB, IRB and NIH as required by local and federal regulations.

8.4 UNANTICIPATED PROBLEMS

8.4.1 DEFINITION OF UNANTICIPATED PROBLEMS (UP)

The Office for Human Research Protections (OHRP) considers unanticipated problems involving risks to participants or others to include, in general, any incident, experience, or outcome that meets **all** of the following criteria:

- Unexpected in terms of nature, severity, or frequency given (a) the research procedures that are described in the protocol-related documents, such as the Institutional Review Board (IRB)-approved research protocol and informed consent document; and (b) the characteristics of the participant population being studied;
- Related or possibly related to participation in the research (“possibly related” means there is a reasonable possibility that the incident, experience, or outcome may have been caused by the procedures involved in the research); and
- Suggests that the research places participants or others at a greater risk of harm (including physical, psychological, economic, or social harm) than was previously known or recognized.

8.4.2 UNANTICIPATED PROBLEM REPORTING

Incidents or events that meet the OHRP criteria for unanticipated problems will initiate the creation and completion of an unanticipated problem report form that will be submitted to DSMB, IRB, FDA, and NIH as outlined. The following information will be included:

- Appropriate identifying information for the research protocol, such as the title, investigator's name, and the IRB project number;
- A detailed description of the adverse event, incident, experience, or outcome;
- An explanation of the basis for determining that the adverse event, incident, experience, or outcome represents an unanticipated problem;
- A description of any changes to the protocol or other corrective actions that have been taken or are proposed in response to the unanticipated problem.

To satisfy the requirement for prompt reporting, unanticipated problems will be reported using the following timeline:

- Unanticipated problems that are serious adverse events will be reported to the IRB, DSMB, FDA, and NIH within 7 days of the investigator becoming aware of the event.
- Any other unanticipated problem will be reported to the IRB, DSMB, and NCCIH within 14 days of the investigator becoming aware of the problem.

8.4.3 REPORTING UNANTICIPATED PROBLEMS TO PARTICIPANTS

Participants will be directly contacted by the study team and brought back for assessment, if necessary.

9 STATISTICAL CONSIDERATIONS

9.1 STATISTICAL HYPOTHESES

The primary objective of this innovative, prospective, longitudinal RCT study is to determine the efficacy of GLP-1RAs in treating lipohypertrophy among non-diabetic people living with HIV (PLWH) by reducing fat accumulation and ectopic fat deposition, altering adipokine levels, improving endothelial function and arterial stiffness, down-regulating key pro-inflammatory cytokines and immune activation without modifying microbial translocation and gut integrity markers (Aims 1 & 2).

9.2 SAMPLE SIZE DETERMINATION

The following sample size estimation and power analysis are based on the significance level 0.05, 90% power, and 20% dropout adjustment. We hypothesize that after the GLP-1RA treatment the primary outcomes of interest (VAT, hsCRP) will be improved in both groups of participants: PLWH and HIV-uninfected controls. Based on Li,[42] we assume that, at baseline, the mean ±SD of VAT in PLWH is 277.80 ± 68.75 cm^2. Using Frison[119] change method for repeated measures design, we estimated the sample size based on the difference in the two groups of the mean change of the post-treatment measurements at week 32 assuming that correlation between repeated measurements is 0.65. Conservatively assuming a mean difference of -34.33 cm^2 (average -42.31, 95% CI: -50.28, -34.33) in VAT change between groups, 43

participants per arm, in a longitudinal measures design with 3 longitudinal measurements, can be detected with 90% power. Similarly, based on Bunck,[72] we assume that the average hsCRP is 1.81 ± 1.37 mg/L at baseline and 1.15 ± 1.20 mg/L at 32 weeks. A mean difference of 0.66 mg/L (1.81–1.15) in hsCRP level change between two groups with 43 participants in each arm can be detected with >90% power. Sample size was inflated to 52 per arm to account for a conservative 20% loss-to-follow up/missing data. We hypothesize that there will be equivalent effects of GLP-1RA treatment on the HIV-uninfected participants as those PLWH, which suggests equal number of enrollment in both groups. Therefore, for the two-parallel studies (PLWH and controls), we will enroll total 104 + 104 = 208 participants. The sample size estimation was performed using STATA 12.0.[120]

9.3 POPULATIONS FOR ANALYSES

104 PLWH (52 active; 52 placebo) will be studied for a total of 56 weeks. During this study period, outcome variables and other measurements will be assessed at various time points. To know their trajectories after discontinuing GLP-1RA treatment, several measurements will also be assessed at week 56. All outcome variables will be analyzed, and estimated values will be portrayed graphically.

Tests for inequality between the treated and placebo groups at 0, 32, and 56 weeks will be performed using likelihood ratio tests with multiple testing corrections using the Benjamini-Hochberg procedure.[116] If there are large numbers of missing values, we will estimate the parameters by utilizing the "pattern mixture model," which assumes liner mixed model. In the presence of outliers and informative dropouts, we will consider using a likelihood-based method developed by Sinha and Sattar[118] for analyzing incomplete longitudinal continuous response measurements with non-ignorable missing and outliers. Alternatively, we may use generalized estimating equation with doubly-robust approach; however, this will be avoided, if possible, because it will lead to decreased power.

9.4 STATISTICAL ANALYSES

9.4.1 GENERAL APPROACH

Study variables, outcomes, and covariates will be summarized using mean, standard deviation, median, and interquartile range cross-sectional and longitudinally by group. The distributions of variables will be examined and necessary transformations (e.g. Box-Cox transformation) will be used to achieve normality with results back transformed for ease of interpretation. The outcome variables are in continuous scale; thus, flexible linear mixed-effects models[115] or a similar repeated measures analysis model (e.g., generalized estimating equations) will be used in analyzing the response trajectories and the benefits of the treatment. We will collect information on the covariates: demographics, health history, and potential moderators including: medication history, dietary and physical activity, HIV RNA, CD4, and weight/BMI. The effects of treatment may be adjusted for these covariates and moderators through the linear mixed effect model analyses.

For reproducibility of the results, the data quality will be reviewed rigorously, and necessary transformations performed to satisfy assumptions related to residuals for parametric models. Basic descriptive statistics will be used to summarize outcomes and covariates within each group of participants.

Statistical analysis will be conducted using software Stata 15.0 software.

9.4.2 ANALYSIS OF THE PRIMARY EFFICACY ENDPOINT(S)

To depict the effects of treatment, summary statistics and predicted values of longitudinally-measured outcome variables will be presented graphically. Models for longitudinally-measured outcome variables, analysis method, and computation of power will be conducted. Two-sided hypothesis tests will be performed assuming a 0.05 level of significance. Type I error rate in multiple comparisons will be controlled by the step-up Benjamini and Hochberg procedure[116] (for small number of comparisons, Bonferroni will be used). However, according to the American Statistical Association statement on p-value, we will emphasize on the magnitude of the outcome variable differences between the groups, and effect sizes in terms of nonzero beta coefficients and its 95% CI rather than adjusted p-values[117].

To evaluate the effects of GLP-1RA treatment, we will analyze primary outcome variables (VAT, hsCRP, and EndoPat) using flexible linear mixed models or a similar repeated measure analysis model (e.g., generalized estimating equations (GEE)). If an outcome distribution is highly skewed and no existing transformation can make it symmetric, we may apply quantile (median) regression for estimating the effects of the treatment. If an outcome distribution is highly skewed and no existing transformation can make it symmetric, we may apply quantile (median) regression for estimating the effects of the treatment. Let Y_{ij} denote an outcome variable from the *i*th participant at the *j*th week, where *i*=1, 2, ..., 104 HIV-infected participants; *j*=0 (entry), 32 weeks. Define T_{ij} to be the week when the measurements are obtained from participant *i*. The repeated measures (e.g., linear mixed) model for the outcome Y_{ij} will be developed. The model may include vectors of covariates, moderators, intervention or the GLP-1RA treatment, and the vectors of random effects $v_i = (v_{0i}, v_{1i})'$ are assumed to be independent multivariate normal with mean vector 0 and covariance matrix, $G(\theta) = G(\sigma_{v11}, \sigma_{v12}, \sigma_{v22})$. The error term in the model is assumed to be distributed as normal$(0, \sigma^2)$, and G is assumed to be an unstructured covariance matrix. From the fitted linear models, we will delineate the predicted outcome variables, association between outcomes, and GLP-1RA treatment. The model parameters will be estimated by the method of (restricted) maximum likelihood or method of moments if GEE is used. Likelihood ratio tests will be used to compare overall mean curves for the groups. Tests for inequality between the treated and placebo groups at 0, 32, and 56 weeks will be performed using likelihood ratio tests with multiple testing corrections using the Benjamini-Hochberg procedure.[116] If there are large numbers of missing values (i.e., non-ignorable missing), we will estimate the parameters by utilizing the "pattern mixture model," which assumes liner mixed model. In the presence of outliers and informative dropouts, we will consider using a likelihood-based method developed by Sinha and Sattar[118] for analyzing incomplete longitudinal continuous response measurements with non-ignorable missing and outliers. Alternatively, we may use generalized estimating equation with doubly-robust approach; however, this will be avoided, if possible, because it will lead to decreased power.

9.4.3 ANALYSIS OF THE SECONDARY ENDPOINT(S)

Refer to section 9.4.2 above.

9.4.4 SAFETY ANALYSES

Each participant will have all graded laboratory and clinical adverse events listed in his/her study chart and will include start/stop dates, severity, and relationship to study intervention, expectedness, outcome, and duration. Graded clinical and laboratory adverse events will then be listed for all participants

collectively and summarized with frequency and percent. Adverse events that occur more than once or with varying degrees of severity in the same participant will be identified, and analyzes will be repeated including only one adverse event per participant (the one with the highest grade or longest duration will be selected). Adverse events leading to premature discontinuation from the study intervention and serious treatment-emergent AEs will be highlighted. Adverse events will also be summarized by study population (HIV and controls) and frequencies between the two groups will be compared. The statistician will also prepare blinded reports based on randomization arm (for all participants and stratified by HIV status) to determine differences in adverse event frequency and severity between study drug and placebo.

9.4.5 BASELINE DESCRIPTIVE STATISTICS

We will collect information on the covariates: demographics, health history, and potential moderators including: medication history, dietary and physical activity, HIV RNA, CD4, and weight/BMI. The effects of treatment will be adjusted for these covariates and moderators through the linear mixed effect model analyses. For reproducibility of the results, the data quality will be reviewed rigorously, and necessary transformations performed to satisfy assumptions related to residuals for parametric models. Basic descriptive statistics will be used to summarize outcomes and covariates within each group of participants.

9.4.6 PLANNED INTERIM ANALYSES

Not applicable.

9.4.7 TABULATION OF INDIVIDUAL PARTICIPANT DATA

Individual participant data will be listed both by measure and time point.

9.4.8 EXPLORATORY ANALYSES

Not applicable.

10 SUPPORTING DOCUMENTATION AND OPERATIONAL CONSIDERATIONS

10.1 REGULATORY, ETHICAL, AND STUDY OVERSIGHT CONSIDERATIONS

10.1.1 INFORMED CONSENT PROCESS

10.1.1.1 CONSENT/ASSENT AND OTHER INFORMATIONAL DOCUMENTS PROVIDED TO PARTICIPANTS

Consent forms describing in detail the study intervention, study procedures, risks, HIPPA/privacy policies, and study staff information will be given to all participants.

10.1.1.2 CONSENT PROCEDURES AND DOCUMENTATION

Consent forms will be reviewed and approved by the institutional review board. Potential participants will be asked to visit the clinic at least once to be screened and ensure that they meet the requirements for entry into the study and are interested in study participation. Potential participants will provide written informed consent before any study procedure can occur. The site PI or designated member of the study team will conduct the informed consent process. The participant will be asked to read and review the document carefully. The PI or designated member of the study team will then explain the research study to the participant and answer any questions that may arise. A verbal explanation will be provided in terms suited to the participant's comprehension of the purposes, procedures, and potential risks of the study and of their rights as research participants. Participants will have the opportunity to further review the written consent form and ask questions prior to signing. The participants will have the opportunity to discuss the study with their family or surrogates or think about it prior to agreeing to participate. Participants will be informed that participation is voluntary and that they may withdraw from the study at any time, without prejudice. If he/she agrees to study participation, the informed consent form will be signed by both the participant and study team member. The site PI or designated member of the study team will complete a checklist indicating date and time the consent was signed, who was present, version date, and if a copy was provided to participant that will be filed in the participant's chart. A copy of the informed consent document will be given to the participants for their records. The site PI will review all signed consent forms for the respective site.

Illiterate Subjects: Subjects with all levels of literacy will be eligible for this study. The consent document will be read to those volunteers with less than an 8th grade equivalent level of literacy. Subsequently, the informed consent will be signed by the volunteer making their mark in the signature section in order to document their understanding. A witness will be present to confirm the consent process has taken place. Both the witness and person conducting the consent process will sign and date the consent. The investigator obtaining consent will ask each subject to reiterate what will be required from them, risks and benefits, and their rights as a participant in order to ensure their full understanding of the study.

Non-English-Speaking Subjects: Subjects who do not understand or speak English will also be eligible for this study. The consent form will be read to those non-English-speaking study candidates in their primary language by a translator. A witness (who speaks English and the study subject's language) will be present to confirm the consent process has taken place. Both the witness and person conducting the consent process will sign and date the consent. The investigator obtaining consent will ask the study candidate via the translator to reiterate what will be required from him/her, risks and benefits, and his/her rights as a participant in order to ensure their full understanding of the study.

10.1.2 STUDY DISCONTINUATION AND CLOSURE

This study may be temporarily suspended or prematurely terminated if there is sufficient reasonable cause. Written notification documenting the reason for study suspension or termination will be provided by the suspending or terminating party to study participants, investigators, NIH, and FDA. If the study is prematurely terminated or suspended, the Principal Investigator (PI) will promptly inform study participants, the Institutional Review Board (IRB), and NIH and will provide the reason(s) for the termination or suspension. Study participants will be contacted, as applicable, and be informed of changes to study visit schedule.

Circumstances that may warrant termination or suspension include, but are not limited to:

1. Determination of unexpected, significant, or unacceptable risk to participants
2. Insufficient compliance to protocol requirements
3. At the discretion of the FDA, IRB, or NIH
4. Data that are not sufficiently complete and/or evaluable
5. Determination that the primary endpoint has been met
6. Determination of futility

Study may resume once concerns about safety, protocol compliance, and data quality are addressed and satisfy the IRB, NIH, and FDA.

10.1.3 CONFIDENTIALITY AND PRIVACY

Efforts will be made to keep all personal information confidential. All research activities will be conducted in as private a setting as possible. Only IRB-approved research staff will have access to identifiable private information about the participants. Patient clinical and laboratory data will be collected on standardized forms which will be kept in locked filling cabinets in locked offices and/or stored on HIPAA-compliant, password-protected, cloud-based storage system "Box" and/or REDcap and only accessible to study staff. Each participant will be assigned a coded number, and this number will be used to identify the participant. All laboratory specimens, evaluation forms, reports, and other records will be identified by coded number only to maintain participant privacy. All computer entry and networking programs will be done with coded numbers only. Any material or data sent to non-study staff personnel will contain only de-identified information. No names or personal information will be used to identify the subjects. Clinical information will not be released to the participant's private physician, clinic, or any other entity without permission of the subject, except as necessary for monitoring by the IRB, FDA, NIH, or as required by law. Release of information will be documented from subjects before requesting any medical records from outside clinics, and associated documents will only be sent/received from HIPAA-compliant, secured fax lines. Study staff will only talk to participants or participants' representatives when phone calls are made for recruitment, retention, or adherence reasons, and any message left will have no mention of HIV. Recruitment material or any information given to participants (e.g., date reminders for study visits) will not mention HIV. Any publication of this study will not use the participant's name or any personal identifiers. Both study sites will ensure that appropriate measures are taken to protect the privacy and confidentiality of all personally-identifiable health information (PHI) for which it is responsible. The proposed studies will include compliance with all regulations set forth by the HIPAA Privacy Rule, as well as with existing State and Federal laws pertaining to PHI.

10.1.4 FUTURE USE OF STORED SPECIMENS AND DATA

With the participant's approval and as approved by local Institutional Review Boards (IRBs), de-identified biological samples of plasma, PBMCs, serum, urine, and stool will be collected and frozen at –80°C for future measurements of cellular immune activation, CVD and metabolic markers, other inflammatory or oxidative factors. Optional genetic material will be stored with participant approval.

During the conduct of the study, an individual participant can choose to withdraw consent to have biological specimens stored for future research. However, withdrawal of consent with regard to biosample storage may not be possible after the study is completed.

10.1.5 KEY ROLES AND STUDY GOVERNANCE

**Principal Investigator**	**Principal Investigator**
Grace A McComsey, MD, FIDSA<br>Professor of Pediatrics and Medicine	Allison Ross Eckard, MD, FIDSA, AAHIVS<br>Associate Professor of Pediatrics and Medicine
Case Western Reserve University	Medical University of South Carolina
Office | LKSD 1400 Mail Stop | LKSD 7061<br>11100 Euclid Ave. Cleveland, OH 44106	135 Rutledge Ave, Ste 1217, MSC 752<br>Charleston, SC 29425-7520
216-844-5936	843-876-1920
Grace.McComsey@UHhospitals.org	eckarda@musc.edu

The **Steering Committee**, comprised of the PIs, key personnel, and consultants will meet every 4 months, or more frequently if necessary, by conference call to discuss study progress, review interim data, and delineate plans for the next 6 months. The Steering Committee will help oversee decisions on minor changes in research direction, publications, data sharing, and reallocation of funds, if necessary.

10.1.6 SAFETY OVERSIGHT

A **data and safety monitoring board (DSMB)** was established to include three physicians (one HIV physician, one endocrinologist, one cardiologist) and a statistician. All DSMB members are independent of the study team. The DSMB will review interim data to detect evidence of adverse events to determine if the trial should continue as originally designed, should be changed, or even stopped based on the data. The DSMB will evaluate the progress of the trial, including periodic assessments of the recruitment goals, protocol adherence, accrual and retention of participants. The DSMB will also function to review and approve plans for data and safety monitoring for this trial, review data on a timely basis and ensure proper conduct and progress of study, review credentials of investigator and project staff, make recommendations to PIs and staff regarding issues of concern, and address adverse events. The DSMB will meet every 6 months (or earlier as needed) in Cleveland, Ohio or by phone conferences to review study protocols, progress, and safety data. Meetings will be held in an open format, except if privileged data are discussed. The DSMB will protect confidentiality of the study participants.

PIs and study team will review all safety labs and clinical assessments on a continual basis. As previously described, all adverse events will be recorded in case report forms and graded per NIH guidelines. All >Grade 1 adverse events deemed possibly-, probably- or definitely-related to study participation will be reported to the local IRBs yearly and to the DSMB as described above and will be presented in scientific publications. All >Grade 2 adverse events deemed to be possibly-, probably- or definitely-related to study participation will be reported to both institutions, the DSMB, NIH, and FDA within one week in writing. DSMB findings will be submitted to the local IRBs with the yearly continuing review (or *ad hoc*, when indicated) in a report prepared by the PIs and study staff and will include enrollment and drop-out rates,

protocol deviations, participant symptoms, and review of clinical and laboratory results. The PIs will notify the NIH, FDA, and DSMB of any action recommended by the IRBs that may affect the conduct of the trial. The PIs will review recommendations from the Steering Committee and all other relevant data available to reach informed decisions.

10.1.7 CLINICAL MONITORING

Clinical monitoring will occur at each respective site by way of the local institution's office of clinical research. Study staff will ask a designated official to perform an audit after the first 1-3 subjects have been enrolled to ensure that the conduction of the trial is in compliance with appropriate protocols and regulations. The monitor will compile a written report that will describe any issues and a point-by-point list of addressable items. The study team will have 30 days to address any issues and provide a written account of how each item was corrected. A complete report will be submitted to the IRB, the office of clinical research, and any other appropriate local or federal agency.

10.1.8 QUALITY ASSURANCE AND QUALITY CONTROL

Each clinical site will perform internal quality management of study conduct, data and biological specimen collection, documentation and completion. An individualized quality management plan will be developed to describe a site's quality management.

Quality control (QC) procedures will be implemented beginning with the data entry system and data QC checks that will be run on the database will be generated. Any missing data or data anomalies will be communicated to the site(s) for clarification/resolution.

Following written Standard Operating Procedures (SOPs), the monitors will verify that the clinical trial is conducted and data are generated and biological specimens are collected, documented (recorded), and reported in compliance with the protocol, International Conference on Harmonisation Good Clinical Practice (ICH GCP), and applicable regulatory requirements (e.g., Good Laboratory Practices (GLP), Good Manufacturing Practices (GMP)).

10.1.9 DATA HANDLING AND RECORD KEEPING.

10.1.9.1 DATA COLLECTION AND MANAGEMENT RESPONSIBILITIES

Clinical data will be collected systematically, captured onto standardized case report forms, and double-entered into REDCap, a secure web-based application that provides case report forms, real-time data entry validation, audit trails, and a de-identified data export mechanism. Data entry fields will be programmed for required entry/range checks. Both the study coordinators and PIs will review all case report forms continually to check for basic problems such as missing data. Periodically, data will be exported from REDCap and reviewed systematically by the biostatistician for errors. Specimens will be obtained and processed according to standardized procedures. Blood samples will be processed and stored appropriately at each respective study site and will be batched for measurements of inflammation, gut integrity, gut hormones, BNPs, adipokines, and other metabolic markers.

Each participating site will maintain appropriate medical and research records for this trial, in compliance with ICH GCP and regulatory and institutional requirements for the protection of confidentiality of participants. As part of participating in a NIH-sponsored or NIH-affiliated study, each site will permit authorized representatives of the NIH, sponsor, and regulatory agencies to examine (and when permitted by applicable law, to copy) clinical records for the purposes of quality assurance reviews, audits, and evaluation of the study safety, progress, and data validity.

10.1.9.2 STUDY RECORDS RETENTION

Study documents will be retained for a length of time in accordance with local and federal regulations.

10.1.10 PROTOCOL DEVIATIONS

A protocol deviation is any noncompliance with the clinical trial protocol, International Conference on Harmonisation Good Clinical Practice (ICH GCP), or Manual of Procedures (MOP) requirements. The noncompliance may be either on the part of the participant, the investigator, or the study site staff. Corrective actions will be developed by the site and implemented promptly.

These practices will be consistent with ICH GCP:

- 4.5 Compliance with Protocol, sections 4.5.1, 4.5.2, and 4.5.3
- 5.1 Quality Assurance and Quality Control, section 5.1.1
- 5.20 Noncompliance, sections 5.20.1, and 5.20.2.

Protocol deviations will be identified by study staff, recorded on a standardized protocol deviation log, and reported to IRB, DSMB, FDA, and NIH as required by local and federal regulations.

10.1.11 PUBLICATION AND DATA SHARING POLICY

Sharing of data generated by this project is an essential part of our proposed activities and will be carried out to comply with the NIH policy on Sharing Research Data Sharing. We wish to make our results available both to the community of scientists interested in HIV infection, immune activation and co-morbidities, as well as to people living with HIV infection. The data generated in this project will be presented at local, national and international conferences and published in peer-reviewed journals in a timely fashion. All final peer-reviewed manuscripts that arise from this project will be submitted to PubMed Central. The PI will also work with the institutions' publicity teams to disseminate findings on their respective websites and LISTSERVS. The PIs will also reach out to several national HIV advocacy groups to further disseminate findings (NATAP.org; thebody.com; IFARA, etc.) The PI will work to facilitate any request made for data produced under this proposal upon publication of data, using standard, university-approved material/data transfer agreements.

10.1.12 CONFLICT OF INTEREST POLICY

Any actual conflict of interest of persons who have a role in the design, conduct, analysis, publication, or any aspect of this trial will be disclosed and managed. Furthermore, persons who have a perceived conflict of interest will be required to have such conflicts managed in a way that is appropriate to their

participation in the design and conduct of this trial. The study leadership in conjunction with the NIH has established policies and procedures for all study group members to disclose all conflicts of interest and will establish a mechanism for the management of all reported dualities of interest.

10.2 ADDITIONAL CONSIDERATIONS

Not applicable.

10.3 ABBREVIATIONS

The list below includes abbreviations utilized in this template. However, this list should be customized for each protocol (i.e., abbreviations not used should be removed and new abbreviations used should be added to this list).

AE	Adverse Event
ANCOVA	Analysis of Covariance
CFR	Code of Federal Regulations
CLIA	Clinical Laboratory Improvement Amendments
CMP	Clinical Monitoring Plan
COC	Certificate of Confidentiality
CONSORT	Consolidated Standards of Reporting Trials
CRF	Case Report Form
DCC	Data Coordinating Center
DHHS	Department of Health and Human Services
DSMB	Data Safety Monitoring Board
DRE	Disease-Related Event
EC	Ethics Committee
eCRF	Electronic Case Report Forms
FDA	Food and Drug Administration
FDAAA	Food and Drug Administration Amendments Act of 2007
FFR	Federal Financial Report
GCP	Good Clinical Practice
GLP	Good Laboratory Practices
GMP	Good Manufacturing Practices
GWAS	Genome-Wide Association Studies
HIPAA	Health Insurance Portability and Accountability Act
IB	Investigator's Brochure
ICH	International Conference on Harmonisation
ICMJE	International Committee of Medical Journal Editors
IDE	Investigational Device Exemption
IND	Investigational New Drug Application
IRB	Institutional Review Board
ISM	Independent Safety Monitor
ISO	International Organization for Standardization
ITT	Intention-To-Treat
LSMEANS	Least-squares Means
MedDRA	Medical Dictionary for Regulatory Activities
MOP	Manual of Procedures

MSDS	Material Safety Data Sheet
NCT	National Clinical Trial
NIH	National Institutes of Health
NIH IC	NIH Institute or Center
OHRP	Office for Human Research Protections
PI	Principal Investigator
QA	Quality Assurance
QC	Quality Control
SAE	Serious Adverse Event
SAP	Statistical Analysis Plan
SMC	Safety Monitoring Committee
SOA	Schedule of Activities
SOC	System Organ Class
SOP	Standard Operating Procedure
UP	Unanticipated Problem
US	United States
HIV	human immunodeficiency virus
PLWH	People living with human immunodeficiency virus
GLP1RA	Glucagon like peptide-1 Receptor Agonist
SC	Subcutaneous
LPS	Lipopolysaccharide
I-FABP	Intestinal fatty acid binding proteins
MMTT	Mixed-meal tolerance test
PWV	Pulse Wave Velocity
PHI	personally-identifiable health information
RCT	Randomized controlled trial
WC	Waist circumference
WHR	Waist-hip ratio
DXA	DEXA Scan
ART	Antiretroviral Therapy

11 REFERENCES

1. McComsey GA, Walker UA. **Role of mitochondria in HIV lipoatrophy: insight into pathogenesis and potential therapies**. *Mitochondrion* 2004; 4(2-3):111-118.
2. McComsey G, Bai RK, Maa JF, Seekins D, Wong LJ. **Extensive investigations of mitochondrial DNA genome in treated HIV-infected subjects: beyond mitochondrial DNA depletion**. *J Acquir Immune Defic Syndr* 2005; 39(2):181-188.
3. McComsey GA, Paulsen DM, Lonergan JT, Hessenthaler SM, Hoppel CL, Williams VC, et al. **Improvements in lipoatrophy, mitochondrial DNA levels and fat apoptosis after replacing stavudine with abacavir or zidovudine**. *AIDS* 2005; 19(1):15-23.
4. McComsey GA, Kitch D, Sax PE, Tebas P, Tierney C, Jahed NC, et al. **Peripheral and central fat changes in subjects randomized to abacavir-lamivudine or tenofovir-emtricitabine with atazanavir-ritonavir or efavirenz: ACTG Study A5224s**. *Clin Infect Dis* 2011; 53(2):185-196. 3165963.
5. McComsey GA, Ward DJ, Hessenthaler SM, Sension MG, Shalit P, Lonergan JT, et al. **Improvement in lipoatrophy associated with highly active antiretroviral therapy in human immunodeficiency virus-infected patients switched from stavudine to abacavir or zidovudine: the results of the TARHEEL study**. *Clin Infect Dis* 2004; 38(2):263-270.
6. Grinspoon S, Carr A. **Cardiovascular risk and body-fat abnormalities in HIV-infected adults**. *N Engl J Med* 2005; 352(1):48-62.
7. McComsey GA, Moser C, Currier J, Ribaudo HJ, Paczuski P, Dube MP, et al. **Body Composition Changes After Initiation of Raltegravir or Protease Inhibitors: ACTG A5260s**. *Clin Infect Dis* 2016; 62(7):853-862. PMC4787610.
8. Bhagwat P, Ofotokun I, McComsey G, Brown T, Moser C, Sugar C, et al. **Predictors of severe weight/body mass index gain following antiretroviral initiation, Abstract 695.** In: *Conference on Retroviruses and Opportunistic Infections*. Seattle, WA; 2017.
9. Thamer C, Machann J, Stefan N, Haap M, Schafer S, Brenner S, et al. **High visceral fat mass and high liver fat are associated with resistance to lifestyle intervention**. *Obesity (Silver Spring)* 2007; 15(2):531-538.
10. Targher G, Bertolini L, Padovani R, Rodella S, Tessari R, Zenari L, et al. **Prevalence of nonalcoholic fatty liver disease and its association with cardiovascular disease among type 2 diabetic patients**. *Diabetes Care* 2007; 30(5):1212-1218.
11. Cherian S, Lopaschuk GD, Carvalho E. **Cellular cross-talk between epicardial adipose tissue and myocardium in relation to the pathogenesis of cardiovascular disease**. *Am J Physiol Endocrinol Metab* 2012; 303(8):E937-949.
12. Iacobellis G, Bianco AC. **Epicardial adipose tissue: emerging physiological, pathophysiological and clinical features**. *Trends Endocrinol Metab* 2011; 22(11):450-457.
13. Sacks HS, Fain JN. **Human epicardial fat: what is new and what is missing?** *Clin Exp Pharmacol Physiol* 2011; 38(12):879-887.
14. Hirata Y, Tabata M, Kurobe H, Motoki T, Akaike M, Nishio C, et al. **Coronary atherosclerosis is associated with macrophage polarization in epicardial adipose tissue**. *J Am Coll Cardiol* 2011; 58(3):248-255.
15. Kremen J, Dolinkova M, Krajickova J, Blaha J, Anderlova K, Lacinova Z, et al. **Increased subcutaneous and epicardial adipose tissue production of proinflammatory cytokines in cardiac surgery patients: possible role in postoperative insulin resistance**. *J Clin Endocrinol Metab* 2006; 91(11):4620-4627.

16. Iacobellis G, Ribaudo MC, Assael F, Vecci E, Tiberti C, Zappaterreno A, et al. **Echocardiographic epicardial adipose tissue is related to anthropometric and clinical parameters of metabolic syndrome: a new indicator of cardiovascular risk**. *J Clin Endocrinol Metab* 2003; 88(11):5163-5168.
17. Rosito GA, Massaro JM, Hoffmann U, Ruberg FL, Mahabadi AA, Vasan RS, et al. **Pericardial fat, visceral abdominal fat, cardiovascular disease risk factors, and vascular calcification in a community-based sample: the Framingham Heart Study**. *Circulation* 2008; 117(5):605-613.
18. Longenecker CT, Jiang Y, Yun CH, Debanne S, Funderburg NT, Lederman MM, et al. **Perivascular fat, inflammation, and cardiovascular risk in HIV-infected patients on antiretroviral therapy**. *Int J Cardiol* 2013; 168(4):4039-4045. 3805774.
19. Longenecker CT, Margevicius S, Liu Y, Schluchter MD, Yun CH, Bezerra HG, et al. **Effect of Pericardial Fat Volume and Density on Markers of Insulin Resistance and Inflammation in Patients With Human Immunodeficiency Virus Infection**. *Am J Cardiol* 2017; 120(8):1427-1433. PMC5614847.
20. Crum-Cianflone N, Roediger MP, Eberly L, Headd M, Marconi V, Ganesan A, et al. **Increasing rates of obesity among HIV-infected persons during the HIV epidemic**. *PLoS One* 2010; 5(4):e10106. PMC2856157.
21. Drucker DJ, Nauck MA. **The incretin system: glucagon-like peptide-1 receptor agonists and dipeptidyl peptidase-4 inhibitors in type 2 diabetes**. *Lancet* 2006; 368(9548):1696-1705.
22. Chia CW, Egan JM. **Incretin-based therapies in type 2 diabetes mellitus**. *J Clin Endocrinol Metab* 2008; 93(10):3703-3716. 2579648.
23. Yang M, Zhang L, Wang C, Liu H, Boden G, Yang G, et al. **Liraglutide increases FGF-21 activity and insulin sensitivity in high fat diet and adiponectin knockdown induced insulin resistance**. *PLoS One* 2012; 7(11):e48392. 3495944.
24. Chia CW, Egan JM. **Role and development of GLP-1 receptor agonists in the management of diabetes**. *Diabetes Metab Syndr Obes* 2009; 2:37. 2818016.
25. Marre M, Shaw J, Brandle M, Bebakar WM, Kamaruddin NA, Strand J, et al. **Liraglutide, a once-daily human GLP-1 analogue, added to a sulphonylurea over 26 weeks produces greater improvements in glycaemic and weight control compared with adding rosiglitazone or placebo in subjects with Type 2 diabetes (LEAD-1 SU)**. *Diabet Med* 2009; 26(3):268-278. 2871176.
26. Nauck M, Frid A, Hermansen K, Shah NS, Tankova T, Mitha IH, et al. **Efficacy and safety comparison of liraglutide, glimepiride, and placebo, all in combination with metformin, in type 2 diabetes: the LEAD (liraglutide effect and action in diabetes)-2 study**. *Diabetes Care* 2009; 32(1):84-90. 2606836.
27. Garber A, Henry R, Ratner R, Garcia-Hernandez PA, Rodriguez-Pattzi H, Olvera-Alvarez I, et al. **Liraglutide versus glimepiride monotherapy for type 2 diabetes (LEAD-3 Mono): a randomised, 52-week, phase III, double-blind, parallel-treatment trial**. *Lancet* 2009; 373(9662):473-481.
28. Zinman B, Gerich J, Buse JB, Lewin A, Schwartz S, Raskin P, et al. **Efficacy and safety of the human glucagon-like peptide-1 analog liraglutide in combination with metformin and thiazolidinedione in patients with type 2 diabetes (LEAD-4 Met+TZD)**. *Diabetes Care* 2009; 32(7):1224-1230. 2699702.
29. Russell-Jones D, Vaag A, Schmitz O, Sethi BK, Lalic N, Antic S, et al. **Liraglutide vs insulin glargine and placebo in combination with metformin and sulfonylurea therapy in type 2 diabetes mellitus (LEAD-5 met+SU): a randomised controlled trial**. *Diabetologia* 2009; 52(10):2046-2055. 2744824.
30. Buse JB, Rosenstock J, Sesti G, Schmidt WE, Montanya E, Brett JH, et al. **Liraglutide once a day versus exenatide twice a day for type 2 diabetes: a 26-week randomised, parallel-group, multinational, open-label trial (LEAD-6)**. *Lancet* 2009; 374(9683):39-47.
31. Wysham CH, MacConell LA, Maggs DG, Zhou M, Griffin PS, Trautmann ME. **Five-Year Efficacy and Safety Data of Exenatide Once Weekly: Long-term Results From the DURATION-1 Randomized Clinical Trial**. *Mayo Clin Proc* 2015; 90(3):356-365.

32. Marso SP, Bain SC, Consoli A, Eliaschewitz FG, Jodar E, Leiter LA, et al. **Semaglutide and Cardiovascular Outcomes in Patients with Type 2 Diabetes**. *N Engl J Med* 2016; 375(19):1834-1844.
33. Marso SP, Daniels GH, Brown-Frandsen K, Kristensen P, Mann JF, Nauck MA, et al. **Liraglutide and Cardiovascular Outcomes in Type 2 Diabetes**. *N Engl J Med* 2016; 375(4):311-322. PMC4985288.
34. Bethel MA, Patel RA, Merrill P, Lokhnygina Y, Buse JB, Mentz RJ, et al. **Cardiovascular outcomes with glucagon-like peptide-1 receptor agonists in patients with type 2 diabetes: a meta-analysis**. *Lancet Diabetes Endocrinol* 2018; 6(2):105-113.
35. Gagnon J, Sauve M, Zhao W, Stacey HM, Wiber SC, Bolz SS, et al. **Chronic Exposure to TNFalpha Impairs Secretion of Glucagon-Like Peptide-1**. *Endocrinology* 2015; 156(11):3950-3960.
36. McComsey G, Moser C, Currier J, Ribaudo H, Paczuski P, Dube M, et al. **Body Composition Changes after Initiation of Raltegravir or Protease Inhibitors-Oral Presentation**. In: *The 22th Conference on Retroviruses and Opportunistic Infections*. Seattle, WA; 2015.
37. McComsey GA, Kitch D, Daar ES, Tierney C, Jahed NC, Melbourne K, et al. **Inflammation markers after randomization to abacavir/lamivudine or tenofovir/emtricitabine with efavirenz or atazanavir/ritonavir**. *AIDS* 2012; 26(11):1371-1385.
38. McComsey G, Kitch D, Sax P, Tierney C, Jahed N, Melbourne K, et al. **Associations of Infammatory Markers with AIDS and non-AIDS Clinical Events after Initiation of Antiretroviral Therapy (ART):AIDS Clinical Trials Group A5224s, a substudy of ACTG A5202.** In: *The XIX International AIDS Conference* Washington, DC; 2012.
39. Ross AC, Rizk N, O'Riordan MA, Dogra V, El-Bejjani D, Storer N, et al. **Relationship between inflammatory markers, endothelial activation markers, and carotid intima-media thickness in HIV-infected patients receiving antiretroviral therapy**. *Clin Infect Dis* 2009; 49(7):1119-1127.
40. Brown TT, Tassiopoulos K, Bosch RJ, Shikuma C, McComsey GA. **Association between systemic inflammation and incident diabetes in HIV-infected patients after initiation of antiretroviral therapy**. *Diabetes Care* 2010; 33(10):2244-2249. 2945167.
41. Astrup A, Rossner S, Van Gaal L, Rissanen A, Niskanen L, Al Hakim M, et al. **Effects of liraglutide in the treatment of obesity: a randomised, double-blind, placebo-controlled study**. *Lancet* 2009; 374(9701):1606-1616.
42. Li CJ, Yu Q, Yu P, Yu TL, Zhang QM, Lu S, et al. **Changes in liraglutide-induced body composition are related to modifications in plasma cardiac natriuretic peptides levels in obese type 2 diabetic patients**. *Cardiovasc Diabetol* 2014; 13:36. 3923001.
43. Jendle J, Nauck MA, Matthews DR, Frid A, Hermansen K, During M, et al. **Weight loss with liraglutide, a once-daily human glucagon-like peptide-1 analogue for type 2 diabetes treatment as monotherapy or added to metformin, is primarily as a result of a reduction in fat tissue**. *Diabetes Obes Metab* 2009; 11(12):1163-1172.
44. Svegliati-Baroni G, Saccomanno S, Rychlicki C, Agostinelli L, De Minicis S, Candelaresi C, et al. **Glucagon-like peptide-1 receptor activation stimulates hepatic lipid oxidation and restores hepatic signalling alteration induced by a high-fat diet in nonalcoholic steatohepatitis**. *Liver Int* 2011; 31(9):1285-1297.
45. Cuthbertson DJ, Irwin A, Gardner CJ, Daousi C, Purewal T, Furlong N, et al. **Improved glycaemia correlates with liver fat reduction in obese, type 2 diabetes, patients given glucagon-like peptide-1 (GLP-1) receptor agonists**. *PLoS One* 2012; 7(12):e50117. 3516516.
46. Longenecker C, Liu Y, Schlucter M, McComsey G. **Pericardial Fat Density: A Novel Marker of Cardiometabolic Risk in HIV Infection**. In: *The 22th Conference on Retroviruses and Opportunistic Infections*. Seattle, WA; 2015.
47. Nauck MA, Petrie JR, Sesti G, Mannucci E, Courreges JP, Lindegaard ML, et al. **A Phase 2, Randomized, Dose-Finding Study of the Novel Once-Weekly Human GLP-1 Analog, Semaglutide, Compared With**

Placebo and Open-Label Liraglutide in Patients With Type 2 Diabetes. *Diabetes Care* 2016; 39(2):231-241.
48. Ahren B, Masmiquel L, Kumar H, Sargin M, Karsbol JD, Jacobsen SH, et al. **Efficacy and safety of once-weekly semaglutide versus once-daily sitagliptin as an add-on to metformin, thiazolidinediones, or both, in patients with type 2 diabetes (SUSTAIN 2): a 56-week, double-blind, phase 3a, randomised trial**. *Lancet Diabetes Endocrinol* 2017; 5(5):341-354.
49. Ahmann AJ, Capehorn M, Charpentier G, Dotta F, Henkel E, Lingvay I, et al. **Efficacy and Safety of Once-Weekly Semaglutide Versus Exenatide ER in Subjects With Type 2 Diabetes (SUSTAIN 3): A 56-Week, Open-Label, Randomized Clinical Trial**. *Diabetes Care* 2018; 41(2):258-266.
50. Taylor SI. **GLP-1 receptor agonists: differentiation within the class**. *Lancet Diabetes Endocrinol* 2018; 6(2):83-85. PMC5988583.
51. Wysham C, Bergenstal R, Malloy J, Yan P, Walsh B, Malone J, et al. **DURATION-2: efficacy and safety of switching from maximum daily sitagliptin or pioglitazone to once-weekly exenatide**. *Diabet Med* 2011; 28(6):705-714. 3123706.
52. Stonehouse A, Walsh B, Cuddihy R. **Exenatide once-weekly clinical development: safety and efficacy across a range of background therapies**. *Diabetes Technol Ther* 2011; 13(10):1063-1069. 3303092.
53. Gerich J. **DPP-4 inhibitors: what may be the clinical differentiators?** *Diabetes Res Clin Pract* 2010; 90(2):131-140.
54. Madsbad S. **Exenatide and liraglutide: different approaches to develop GLP-1 receptor agonists (incretin mimetics)--preclinical and clinical results**. *Best Pract Res Clin Endocrinol Metab* 2009; 23(4):463-477.
55. Houlden R, Ross S, Harris S, Yale JF, Sauriol L, Gerstein HC. **Treatment satisfaction and quality of life using an early insulinization strategy with insulin glargine compared to an adjusted oral therapy in the management of Type 2 diabetes: the Canadian INSIGHT Study**. *Diabetes Res Clin Pract* 2007; 78(2):254-258.
56. Nicolucci A, Cucinotta D, Squatrito S, Lapolla A, Musacchio N, Leotta S, et al. **Clinical and socio-economic correlates of quality of life and treatment satisfaction in patients with type 2 diabetes**. *Nutr Metab Cardiovasc Dis* 2009; 19(1):45-53.
57. Pratley R, Nauck M, Bailey T, Montanya E, Cuddihy R, Filetti S, et al. **One year of liraglutide treatment offers sustained and more effective glycaemic control and weight reduction compared with sitagliptin, both in combination with metformin, in patients with type 2 diabetes: a randomised, parallel-group, open-label trial**. *Int J Clin Pract* 2011; 65(4):397-407. 3085127.
58. Best JH, Rubin RR, Peyrot M, Li Y, Yan P, Malloy J, et al. **Weight-related quality of life, health utility, psychological well-being, and satisfaction with exenatide once weekly compared with sitagliptin or pioglitazone after 26 weeks of treatment**. *Diabetes Care* 2011; 34(2):314-319. 3024340.
59. Davies M, Pratley R, Hammer M, Thomsen AB, Cuddihy R. **Liraglutide improves treatment satisfaction in people with Type 2 diabetes compared with sitagliptin, each as an add on to metformin**. *Diabet Med* 2011; 28(3):333-337.
60. Pratley RE, Nauck MA, Bailey T, Montanya E, Filetti S, Garber AJ, et al. **Efficacy and safety of switching from the DPP-4 inhibitor sitagliptin to the human GLP-1 analog liraglutide after 52 weeks in metformin-treated patients with type 2 diabetes: a randomized, open-label trial**. *Diabetes Care* 2012; 35(10):1986-1993. 3447855.
61. Flint A, Raben A, Ersboll AK, Holst JJ, Astrup A. **The effect of physiological levels of glucagon-like peptide-1 on appetite, gastric emptying, energy and substrate metabolism in obesity**. *Int J Obes Relat Metab Disord* 2001; 25(6):781-792.
62. van Bloemendaal L, Ten Kulve JS, la Fleur SE, Ijzerman RG, Diamant M. **Effects of glucagon-like peptide 1 on appetite and body weight: focus on the CNS**. *J Endocrinol* 2014; 221(1):T1-16.

63. Flint A, Raben A, Astrup A, Holst JJ. **Glucagon-like peptide 1 promotes satiety and suppresses energy intake in humans**. *J Clin Invest* 1998; 101(3):515-520. 508592.
64. Verdich C, Flint A, Gutzwiller JP, Naslund E, Beglinger C, Hellstrom PM, et al. **A meta-analysis of the effect of glucagon-like peptide-1 (7-36) amide on ad libitum energy intake in humans**. *J Clin Endocrinol Metab* 2001; 86(9):4382-4389.
65. Gutzwiller JP, Drewe J, Goke B, Schmidt H, Rohrer B, Lareida J, et al. **Glucagon-like peptide-1 promotes satiety and reduces food intake in patients with diabetes mellitus type 2**. *Am J Physiol* 1999; 276(5 Pt 2):R1541-1544.
66. Flint A, Kapitza C, Hindsberger C, Zdravkovic M. **The once-daily human glucagon-like peptide-1 (GLP-1) analog liraglutide improves postprandial glucose levels in type 2 diabetes patients**. *Adv Ther* 2011; 28(3):213-226.
67. Shalev A, Holst JJ, Keller U. **Effects of glucagon-like peptide 1 (7-36 amide) on whole-body protein metabolism in healthy man**. *Eur J Clin Invest* 1997; 27(1):10-16.
68. Kim Chung le T, Hosaka T, Yoshida M, Harada N, Sakaue H, Sakai T, et al. **Exendin-4, a GLP-1 receptor agonist, directly induces adiponectin expression through protein kinase A pathway and prevents inflammatory adipokine expression**. *Biochem Biophys Res Commun* 2009; 390(3):613-618.
69. Ohki T, Isogawa A, Iwamoto M, Ohsugi M, Yoshida H, Toda N, et al. **The effectiveness of liraglutide in nonalcoholic fatty liver disease patients with type 2 diabetes mellitus compared to sitagliptin and pioglitazone**. *ScientificWorldJournal* 2012; 2012:496453. 3425807.
70. Vendrell J, El Bekay R, Peral B, Garcia-Fuentes E, Megia A, Macias-Gonzalez M, et al. **Study of the potential association of adipose tissue GLP-1 receptor with obesity and insulin resistance**. *Endocrinology* 2011; 152(11):4072-4079.
71. Cantini G, Di Franco A, Samavat J, Forti G, Mannucci E, Luconi M. **Effect of liraglutide on proliferation and differentiation of human adipose stem cells**. *Mol Cell Endocrinol* 2015; 402:43-50.
72. Bunck MC, Diamant M, Eliasson B, Corner A, Shaginian RM, Heine RJ, et al. **Exenatide affects circulating cardiovascular risk biomarkers independently of changes in body composition**. *Diabetes Care* 2010; 33(8):1734-1737. 2909051.
73. Yang M, Liu R, Li S, Luo Y, Zhang Y, Zhang L, et al. **Zinc-alpha2-glycoprotein is associated with insulin resistance in humans and is regulated by hyperglycemia, hyperinsulinemia, or liraglutide administration: cross-sectional and interventional studies in normal subjects, insulin-resistant subjects, and subjects with newly diagnosed diabetes**. *Diabetes Care* 2013; 36(5):1074-1082. 3631846.
74. Calvani M, Scarfone A, Granato L, Mora EV, Nanni G, Castagneto M, et al. **Restoration of adiponectin pulsatility in severely obese subjects after weight loss**. *Diabetes* 2004; 53(4):939-947.
75. Diaz-Soto G, de Luis DA, Conde-Vicente R, Izaola-Jauregui O, Ramos C, Romero E. **Beneficial effects of liraglutide on adipocytokines, insulin sensitivity parameters and cardiovascular risk biomarkers in patients with Type 2 diabetes: a prospective study**. *Diabetes Res Clin Pract* 2014; 104(1):92-96.
76. Kim M, Platt MJ, Shibasaki T, Quaggin SE, Backx PH, Seino S, et al. **GLP-1 receptor activation and Epac2 link atrial natriuretic peptide secretion to control of blood pressure**. *Nat Med* 2013; 19(5):567-575.
77. Sengenes C, Berlan M, De Glisezinski I, Lafontan M, Galitzky J. **Natriuretic peptides: a new lipolytic pathway in human adipocytes**. *FASEB J* 2000; 14(10):1345-1351.
78. Moro C, Pillard F, de Glisezinski I, Klimcakova E, Crampes F, Thalamas C, et al. **Exercise-induced lipid mobilization in subcutaneous adipose tissue is mainly related to natriuretic peptides in overweight men**. *Am J Physiol Endocrinol Metab* 2008; 295(2):E505-513.
79. Birkenfeld AL, Budziarek P, Boschmann M, Moro C, Adams F, Franke G, et al. **Atrial natriuretic peptide induces postprandial lipid oxidation in humans**. *Diabetes* 2008; 57(12):3199-3204. 2584124.

80. Bordicchia M, Liu D, Amri EZ, Ailhaud G, Dessi-Fulgheri P, Zhang C, et al. **Cardiac natriuretic peptides act via p38 MAPK to induce the brown fat thermogenic program in mouse and human adipocytes**. *J Clin Invest* 2012; 122(3):1022-1036. 3287224.
81. Chen-Tournoux A, Khan AM, Baggish AL, Castro VM, Semigran MJ, McCabe EL, et al. **Effect of weight loss after weight loss surgery on plasma N-terminal pro-B-type natriuretic peptide levels**. *Am J Cardiol* 2010; 106(10):1450-1455. 3170817.
82. Chainani-Wu N, Weidner G, Purnell DM, Frenda S, Merritt-Worden T, Kemp C, et al. **Relation of B-type natriuretic peptide levels to body mass index after comprehensive lifestyle changes**. *Am J Cardiol* 2010; 105(11):1570-1576.
83. Lee YS, Park MS, Choung JS, Kim SS, Oh HH, Choi CS, et al. **Glucagon-like peptide-1 inhibits adipose tissue macrophage infiltration and inflammation in an obese mouse model of diabetes**. *Diabetologia* 2012; 55(9):2456-2468.
84. Chaudhuri A, Ghanim H, Vora M, Sia CL, Korzeniewski K, Dhindsa S, et al. **Exenatide exerts a potent antiinflammatory effect**. *J Clin Endocrinol Metab* 2012; 97(1):198-207. 3251936.
85. Horton E, Cohen A, Gibson H, Lamparello B, Herzlinger S, McFarland L. **Effects of exenatide vs insulin glargine on cardiovascular risk factors in subjects with type 2 diabetes**. *Diabetologia* 2009; 52:S298-S299.
86. Doggrell SA. **Sgemaglutide in type 2 diabetes - is it the best glucagon-like peptide 1 receptor agonist (GLP-1R agonist)?** *Expert Opin Drug Metab Toxicol* 2018; 14(3):371-377.
87. Fujishima Y, Maeda N, Inoue K, Kashine S, Nishizawa H, Hirata A, et al. **Efficacy of liraglutide, a glucagon-like peptide-1 (GLP-1) analogue, on body weight, eating behavior, and glycemic control, in Japanese obese type 2 diabetes**. *Cardiovasc Diabetol* 2012; 11:107. 3459720.
88. Astrup A, Carraro R, Finer N, Harper A, Kunesova M, Lean ME, et al. **Safety, tolerability and sustained weight loss over 2 years with the once-daily human GLP-1 analog, liraglutide**. *Int J Obes (Lond)* 2012; 36(6):843-854. 3374073.
89. Hattori Y, Jojima T, Tomizawa A, Satoh H, Hattori S, Kasai K, et al. **A glucagon-like peptide-1 (GLP-1) analogue, liraglutide, upregulates nitric oxide production and exerts anti-inflammatory action in endothelial cells**. *Diabetologia* 2010; 53(10):2256-2263.
90. Irace C, De Luca S, Shehaj E, Carallo C, Loprete A, Scavelli F, et al. **Exenatide improves endothelial function assessed by flow mediated dilation technique in subjects with type 2 diabetes: results from an observational research**. *Diab Vasc Dis Res* 2013; 10(1):72-77.
91. Nystrom T, Gutniak MK, Zhang Q, Zhang F, Holst JJ, Ahren B, et al. **Effects of glucagon-like peptide-1 on endothelial function in type 2 diabetes patients with stable coronary artery disease**. *Am J Physiol Endocrinol Metab* 2004; 287(6):E1209-1215.
92. Tounian P, Aggoun Y, Dubern B, Varille V, Guy-Grand B, Sidi D, et al. **Presence of increased stiffness of the common carotid artery and endothelial dysfunction in severely obese children: a prospective study**. *Lancet* 2001; 358(9291):1400-1404.
93. Duffy SJ, Keaney JF, Jr., Holbrook M, Gokce N, Swerdloff PL, Frei B, et al. **Short- and long-term black tea consumption reverses endothelial dysfunction in patients with coronary artery disease**. *Circulation* 2001; 104(2):151-156.
94. Kim W, Jeong MH, Cho SH, Yun JH, Chae HJ, Ahn YK, et al. **Effect of green tea consumption on endothelial function and circulating endothelial progenitor cells in chronic smokers**. *Circ J* 2006; 70(8):1052-1057.
95. Zhu Y, Xia M, Yang Y, Liu F, Li Z, Hao Y, et al. **Purified anthocyanin supplementation improves endothelial function via NO-cGMP activation in hypercholesterolemic individuals**. *Clin Chem* 2011; 57(11):1524-1533.

96. Dong Y, Stallmann-Jorgensen IS, Pollock NK, Harris RA, Keeton D, Huang Y, et al. **A 16-week randomized clinical trial of 2000 international units daily vitamin D3 supplementation in black youth: 25-hydroxyvitamin D, adiposity, and arterial stiffness**. *J Clin Endocrinol Metab* 2010; 95(10):4584-4591.
97. Mitchell GF, Hwang SJ, Vasan RS, Larson MG, Pencina MJ, Hamburg NM, et al. **Arterial stiffness and cardiovascular events: the Framingham Heart Study**. *Circulation* 2010; 121(4):505-511. 2836717.
98. Nurnberger J, Keflioglu-Scheiber A, Opazo Saez AM, Wenzel RR, Philipp T, Schafers RF. **Augmentation index is associated with cardiovascular risk**. *J Hypertens* 2002; 20(12):2407-2414.
99. Halcox JP, Schenke WH, Zalos G, Mincemoyer R, Prasad A, Waclawiw MA, et al. **Prognostic value of coronary vascular endothelial dysfunction**. *Circulation* 2002; 106(6):653-658.
100. Mano T, Masuyama T, Yamamoto K, Naito J, Kondo H, Nagano R, et al. **Endothelial dysfunction in the early stage of atherosclerosis precedes appearance of intimal lesions assessable with intravascular ultrasound**. *Am Heart J* 1996; 131(2):231-238.
101. Muscelli E, Mari A, Casolaro A, Camastra S, Seghieri G, Gastaldelli A, et al. **Separate impact of obesity and glucose tolerance on the incretin effect in normal subjects and type 2 diabetic patients**. *Diabetes* 2008; 57(5):1340-1348.
102. Nauck M, Stockmann F, Ebert R, Creutzfeldt W. **Reduced incretin effect in type 2 (non-insulin-dependent) diabetes**. *Diabetologia* 1986; 29(1):46-52.
103. Alssema M, Rijkelijkhuizen JM, Holst JJ, Teerlink T, Scheffer PG, Eekhoff EM, et al. **Preserved GLP-1 and exaggerated GIP secretion in type 2 diabetes and relationships with triglycerides and ALT**. *Eur J Endocrinol* 2013; 169(4):421-430.
104. Vollmer K, Holst JJ, Baller B, Ellrichmann M, Nauck MA, Schmidt WE, et al. **Predictors of incretin concentrations in subjects with normal, impaired, and diabetic glucose tolerance**. *Diabetes* 2008; 57(3):678-687.
105. Nauck MA, Vardarli I, Deacon CF, Holst JJ, Meier JJ. **Secretion of glucagon-like peptide-1 (GLP-1) in type 2 diabetes: what is up, what is down?** *Diabetologia* 2011; 54(1):10-18.
106. Sharkey KA, Sutherland LR, Davison JS, Zwiers H, Gill MJ, Church DL. **Peptides in the gastrointestinal tract in human immunodeficiency virus infection. The GI/HIV Study Group of the University of Calgary**. *Gastroenterology* 1992; 103(1):18-28.
107. Schmitz H, Fromm M, Bentzel CJ, Scholz P, Detjen K, Mankertz J, et al. **Tumor necrosis factor-alpha (TNFalpha) regulates the epithelial barrier in the human intestinal cell line HT-29/B6**. *J Cell Sci* 1999; 112 (Pt 1):137-146.
108. Wensveen FM, Valentic S, Sestan M, Turk Wensveen T, Polic B. **The "Big Bang" in obese fat: Events initiating obesity-induced adipose tissue inflammation**. *Eur J Immunol* 2015; 45(9):2446-2456.
109. Li X, Watanabe K, Kimura I. **Gut Microbiota Dysbiosis Drives and Implies Novel Therapeutic Strategies for Diabetes Mellitus and Related Metabolic Diseases**. *Front Immunol* 2017; 8:1882. PMC5742320.
110. Bouter KE, van Raalte DH, Groen AK, Nieuwdorp M. **Role of the Gut Microbiome in the Pathogenesis of Obesity and Obesity-Related Metabolic Dysfunction**. *Gastroenterology* 2017; 152(7):1671-1678.
111. Lackner AA, Mohan M, Veazey RS. **The gastrointestinal tract and AIDS pathogenesis**. *Gastroenterology* 2009; 136(6):1965-1978. PMC3755960.
112. Kelesidis T, Kendall MA, Yang OO, Hodis HN, Currier JS. **Biomarkers of microbial translocation and macrophage activation: association with progression of subclinical atherosclerosis in HIV-1 infection**. *J Infect Dis* 2012; 206(10):1558-1567. PMC3475633.
113. Longenecker CT, Jiang Y, Orringer CE, Gilkeson RC, Debanne S, Funderburg NT, et al. **Soluble CD14 is independently associated with coronary calcification and extent of subclinical vascular disease in treated HIV infection**. *AIDS* 2014; 28(7):969-977. 4097603.

114. McComsey G, Kitch D, Sax PE, Tebas P, Tierney C, Jahed N, et al. **Peripheral and central fat Changes in Subjects Randomized to Abacavir/Lamivudine or Tenofovir/Emtricitabine with Atazanavir/Ritonavir or Efavirenz: ACTG study A5224s**. *CID* 2011.
115. Fitzmaurice G, Laird NM, Ware J. **Applied Longitudinal Analysis**. 2nd edition ed: Wiley Interscience; 2011.
116. Benjamini Y, Hochberg Y. **Journal of the Royal Statistical Society, Series B 57** 1995.
117. Wasserstein R, Lazar N. **The ASA's statement on p-values: context, process, and purpose**. *The American Statistician* 2016; 70(2):129-133.
118. Sinha SK, Sattar A. **Analysis of incomplete longitudinal data with informative drop-outs and outliers**. *Canadian Journals of Statistics* 2014; 42(4):670-695.
119. Frison L, Pocock J. **Repeated measures in clinical trials: Analysis using mean summary statistics and its implications for design.** . 1992.
120. **Stat Statistical Software Release 12**. In. College Station, TX: StatCorp LP; 2011.
121. Tien PC, Benson C, Zolopa AR, Sidney S, Osmond D, Grunfeld C. **The study of fat redistribution and metabolic change in HIV infection (FRAM): methods, design, and sample characteristics**. *Am J Epidemiol* 2006; 163(9):860-869. 3170407.
122. McComsey GA, Kendall MA, Tebas P, Swindells S, Hogg E, Alston-Smith B, et al. **Alendronate with calcium and vitamin D supplementation is safe and effective for the treatment of decreased bone mineral density in HIV**. *AIDS* 2007; 21(18):2473-2482.
123. McComsey GA, Walker UA, Budhathoki CB, Su Z, Currier JS, Kosmiski L, et al. **Uridine supplementation in the treatment of HIV lipoatrophy: results of ACTG 5229**. *AIDS* 2010; 24(16):2507-2515. 2956768.
124. McComsey GA, O'Riordan M, Choi J, Libutti D, Rowe D, Storer N, et al. **Mitochondrial function, inflammation, fat and bone in HIV lipoatrophy: randomized study of uridine supplementation or switch to tenofovir**. *Antivir Ther* 2012; 17(2):347-353. 3302940.
125. Weir JB. **New methods for calculating metabolic rate with special reference to protein metabolism**. *J Physiol* 1949; 109(1-2):1-9. 1392602.
126. Mittelsteadt AL, Hileman CO, Harris SR, Payne KM, Gripshover BM, McComsey GA. **Effects of HIV and antiretroviral therapy on resting energy expenditure in adult HIV-infected women-a matched, prospective, cross-sectional study**. *J Acad Nutr Diet* 2013; 113(8):1037-1043.
127. Dirajlal-Fargo S, Kinley B, Jiang Y, Longenecker CT, Hileman CO, Debanne S, et al. **Statin therapy decreases N-terminal pro-B-type natriuretic peptide in HIV: randomized placebo-controlled trial**. *AIDS* 2015; 29(3):313-321.
128. Longenecker CT, Dunn W, Jiang Y, Debanne SM, McComsey GA. **Adipokines and vascular health in treated HIV infection: an obesity paradox?** *AIDS* 2013; 27(8):1353-1356. 4140858.
129. Lee CJ, Brown T, Magnuson TH, Egan JM, Carlson O, Elahi D. **Hormonal response to a mixed-meal challenge after reversal of gastric bypass for hypoglycemia**. *J Clin Endocrinol Metab* 2013; 98(7):E1208-1212. PMC5393460.
130. Chia CW, Carlson OD, Kim W, Shin YK, Charles CP, Kim HS, et al. **Exogenous glucose-dependent insulinotropic polypeptide worsens post prandial hyperglycemia in type 2 diabetes**. *Diabetes* 2009; 58(6):1342-1349. PMC2682676.
131. Getty-Kaushik L, Song DH, Boylan MO, Corkey BE, Wolfe MM. **Glucose-dependent insulinotropic polypeptide modulates adipocyte lipolysis and reesterification**. *Obesity (Silver Spring)* 2006; 14(7):1124-1131.
132. Yip RG, Wolfe MM. **GIP biology and fat metabolism**. *Life Sci* 2000; 66(2):91-103.

133. Varol C, Zvibel I, Spektor L, Mantelmacher FD, Vugman M, Thurm T, et al. **Long-acting glucose-dependent insulinotropic polypeptide ameliorates obesity-induced adipose tissue inflammation**. *J Immunol* 2014; 193(8):4002-4009.
134. Burdo TH, Lo J, Abbara S, Wei J, DeLelys ME, Preffer F, et al. **Soluble CD163, a novel marker of activated macrophages, is elevated and associated with noncalcified coronary plaque in HIV-infected patients**. *J Infect Dis* 2011; 204(8):1227-1236. PMC3203384.
135. Subramanian S, Tawakol A, Burdo TH, Abbara S, Wei J, Vijayakumar J, et al. **Arterial inflammation in patients with HIV**. *JAMA* 2012; 308(4):379-386. PMC3724172.
136. Hileman CO, Labbato DE, Storer NJ, Tangpricha V, McComsey GA. **Is bone loss linked to chronic inflammation in antiretroviral-naive HIV-infected adults? A 48-week matched cohort study**. *AIDS* 2014; 28(12):1759-1767.
137. Beck J, Ferrucci L, Sun K, Fried LP, Varadhan R, Walston J, et al. **Circulating oxidized low-density lipoproteins are associated with overweight, obesity, and low serum carotenoids in older community-dwelling women**. *Nutrition* 2008; 24(10):964-968. 2656364.
138. Semba RD, Ferrucci L, Sun K, Walston J, Varadhan R, Guralnik JM, et al. **Oxidative stress is associated with greater mortality in older women living in the community**. *J Am Geriatr Soc* 2007; 55(9):1421-1425. 2645668.
139. Erlandson K, Jiang Y, debanne S, McComsey G. **Rosuvastatin Worsens Insulin Resistance in HIV-Infected Adults on Antiretroviral Therapy**. *Clinical Infectious Disease* 2015.
140. Muniyappa R, Lee S, Chen H, Quon MJ. **Current approaches for assessing insulin sensitivity and resistance in vivo: advantages, limitations, and appropriate usage**. *Am J Physiol Endocrinol Metab* 2008; 294(1):E15-26.
141. Pelsers MM, Namiot Z, Kisielewski W, Namiot A, Januszkiewicz M, Hermens WT, et al. **Intestinal-type and liver-type fatty acid-binding protein in the intestine. Tissue distribution and clinical utility**. *Clin Biochem* 2003; 36(7):529-535.
142. Fasano A. **Zonulin and its regulation of intestinal barrier function: the biological door to inflammation, autoimmunity, and cancer**. *Physiol Rev* 2011; 91(1):151-175.
143. Pacifico L, Bonci E, Marandola L, Romaggioli S, Bascetta S, Chiesa C. **Increased circulating zonulin in children with biopsy-proven nonalcoholic fatty liver disease**. *World J Gastroenterol* 2014; 20(45):17107-17114. 4258579.
144. Zhang D, Zhang L, Yue F, Zheng Y, Russell R. **Serum zonulin is elevated in women with polycystic ovary syndrome and correlates with insulin resistance and severity of anovulation**. *Eur J Endocrinol* 2015; 172(1):29-36.
145. Hunt PW, Sinclair E, Rodriguez B, Shive C, Clagett B, Funderburg N, et al. **Gut epithelial barrier dysfunction and innate immune activation predict mortality in treated HIV infection**. *J Infect Dis* 2014; 210(8):1228-1238. 4192038.
146. Salvi P, Lio G, Labat C, Ricci E, Pannier B, Benetos A. **Validation of a new non-invasive portable tonometer for determining arterial pressure wave and pulse wave velocity: the PulsePen device**. *J Hypertens* 2004; 22(12):2285-2293.
147. Rouxinol-Dias A, Araujo S, Silva JA, Barbosa L, Polonia J. **Association between ambulatory blood pressure values and central aortic pressure in a large population of normotensive and hypertensive patients**. *Blood Press Monit* 2018; 23(1):24-32.
148. Eckard AR, Raggi P, Ruff JH, O'Riordan MA, Rosebush JC, Labbato D, et al. **Arterial stiffness in HIV-infected youth and associations with HIV-related variables**. *Virulence* 2017:1-9.
149. Takase B, Uehata A, Akima T, Nagai T, Nishioka T, Hamabe A, et al. **Endothelium-dependent flow-mediated vasodilation in coronary and brachial arteries in suspected coronary artery disease**. *Am J Cardiol* 1998; 82(12):1535-1539, A1537-1538.

150. Malm-Erjefalt M, Bjornsdottir I, Vanggaard J, Helleberg H, Larsen U, Oosterhuis B, et al. **Metabolism and excretion of the once-daily human glucagon-like peptide-1 analog liraglutide in healthy male subjects and its in vitro degradation by dipeptidyl peptidase IV and neutral endopeptidase**. *Drug Metab Dispos* 2010; 38(11):1944-1953.
