# Supplementary material for: Semaglutide slows epigenetic aging in a randomized trial of HIV-associated lipohypertrophy
Source: Nat Commun. 2026 May 19;17:6606. doi: 10.1038/s41467-026-72861-3 (PMC13381876; doi:10.1038/s41467-026-72861-3)
Supplement: Supplementary file 2 — Reporting summary [file 41467_2026_72861_MOESM2_ESM.pdf]

nature portfolio

Corresponding author(s): *Double-anonymous peer review submissions: write DAPR and your manuscript number here instead of author names.*

Last updated by author(s): 05/07/2026

# Reporting Summary

Nature Portfolio wishes to improve the reproducibility of the work that we publish. This form provides structure for consistency and transparency in reporting. For further information on Nature Portfolio policies, see our Editorial Policies and the Editorial Policy Checklist.

## Statistics

For all statistical analyses, confirm that the following items are present in the figure legend, table legend, main text, or Methods section.

| n/a | Confirmed | |
|---|---|---|
| ☐ | ☒ | The exact sample size (*n*) for each experimental group/condition, given as a discrete number and unit of measurement |
| ☐ | ☒ | A statement on whether measurements were taken from distinct samples or whether the same sample was measured repeatedly |
| ☐ | ☒ | The statistical test(s) used AND whether they are one- or two-sided<br>*Only common tests should be described solely by name; describe more complex techniques in the Methods section.* |
| ☐ | ☒ | A description of all covariates tested |
| ☐ | ☒ | A description of any assumptions or corrections, such as tests of normality and adjustment for multiple comparisons |
| ☐ | ☒ | A full description of the statistical parameters including central tendency (e.g. means) or other basic estimates (e.g. regression coefficient) AND variation (e.g. standard deviation) or associated estimates of uncertainty (e.g. confidence intervals) |
| ☐ | ☒ | For null hypothesis testing, the test statistic (e.g. *F*, *t*, *r*) with confidence intervals, effect sizes, degrees of freedom and *P* value noted<br>*Give P values as exact values whenever suitable.* |
| ☒ | ☐ | For Bayesian analysis, information on the choice of priors and Markov chain Monte Carlo settings |
| ☒ | ☐ | For hierarchical and complex designs, identification of the appropriate level for tests and full reporting of outcomes |
| ☐ | ☒ | Estimates of effect sizes (e.g. Cohen's *d*, Pearson's *r*), indicating how they were calculated |

*Our web collection on statistics for biologists contains articles on many of the points above.*

## Software and code

Policy information about availability of computer code

| | |
|---|---|
| Data collection | Genome-wide DNA methylation profiling was performed using the Infinium MethylationEPICV2 BeadChip (Illumina), which covers >850,000 CpG sites, following manufacturer protocols. |
| Data analysis | We examined three generations of epigenetic clocks: first-generation clocks that estimate chronological age (Horvath1, Horvath2, and Hannum clocks); second-generation clocks that predict mortality/morbidity risk (PhenoAge and GrimAge, including a principal-component version of GrimAge denoted PCGrimAge); and a third-generation clock known as DunedinPACE, which measures the pace of aging (years aged per chronological year). To improve robustness for longitudinal analysis, we used principal component-based versions of the first- and second-generation clocks (denoted "PC clocks") wherever applicable. These PC clocks leverage principal components of DNAm data associated with the original clock algorithms, enhancing technical reliability and reducing noise in repeated measures. Published epigenetic clocks were calculated according to published methods from processed DNA methylation data. To calculate the principal component-based epigenetic clock for the Horvath multi-tissue clock, Hannum clock, DNAmPhenoAge clock, GrimAge clock, and telomere length we used the custom R script available via GitHub (https://github.com/MorganLevineLab/PC-Clocks). Non-principal component-based (non-PC) Horvath, Hannum, and DNAmPhenoAge epigenetic metrics were calculated using the methyAge function in the ENMix R package. The pace of aging clock, DunedinPACE, was calculated using the PACEProjector function from the DunedinPACE package available via GitHub (https://github.com/danbelsky/DunedinPACE). We used a 12 cell immune deconvolution method to estimate cell type proportions42. For Biolearn, DNA methylation beta values (ssNoob-normalized) and matched sample metadata were imported into R (v4.3.2). Python integration was managed via the reticulate package, linking to a virtual environment with BioLearn installed. Missing CpGs were imputed using dataset-wide means via impute_from_average(), and the resulting matrix was combined with metadata into a GeoDataobject. The GrimAgeV1 and GrimAgeV2 models, obtained from the BioLearn ModelGallery, were applied using default parameters. Both models are based on Cox proportional hazards regression, trained to predict time-to-death from DNA methylation profiles. Internally, the models first extract a subset of CpGs relevant to DNAm surrogates for plasma proteins and smoking pack-years, followed by transformation through weighted linear combinations. |

These component predictors are then integrated into a multivariate Cox-PH model to estimate mortality risk, which is scaled to generate biological age equivalents.: All epigenetic clock algorithms were applied using publicly available code and R/Python packages. Principal component–based epigenetic clocks (PCHorvath1, PCHorvath2, PCHannum, PCPhenoAge, PCGrimAge) were calculated using the PC-Clocks repository (https://github.com/albertchen42/PC-Clocks). Non-PC Horvath, Hannum, and DNAmPhenoAge clocks were calculated using the methyAge function in the ENmix R package (v1.44.3). DunedinPACE was calculated using the PACEProjector function from the DunedinPACE R package (v0.99.0; https://github.com/danbelsky/DunedinPACE). GrimAge V1 and V2 were computed using the BioLearn Python library (https://bio-learn.github.io/). OMICmAge was calculated using code available at https://github.com/LaskySuLab/OMICmAge/. CausAge, DamAge, and AdaptAge were calculated using algorithms available in the Supplementary Information of the original publication and via the ClockBase platform (www.clockbase.org) and the BioLearn Python package (https://bio-learn.github.io/). RetroAge was calculated using retroelement-based clock coefficients and scripts deposited on Zenodo (DOI: 10.5281/zenodo.11099870), which support DNA methylation EPIC v1.0 and v2.0 datasets. SystemsAge was calculated using the methylCIPHER package (https://github.com/HigginsChenLab/methylCIPHER). The Intrinsic Capacity epigenetic clock was calculated using code available at https://github.com/msfuentealba/IC_clock. DNAmFitAge and additional epigenetic metrics were calculated via TruDiagnostic's DNAm Analysis Software, available at no cost for research purposes upon request (https://www.trudiagnostic.com/softwarerequest/); access requires agreement to a standard noncommercial software license for academic, nonprofit research use. DNA methylation preprocessing was performed using the minfi R package (v1.54.1). No custom analysis code beyond the application of these published tools was developed for this study.

For manuscripts utilizing custom algorithms or software that are central to the research but not yet described in published literature, software must be made available to editors and reviewers. We strongly encourage code deposition in a community repository (e.g. GitHub). See the Nature Portfolio guidelines for submitting code & software for further information.

## Data

Policy information about availability of data

All manuscripts must include a data availability statement. This statement should provide the following information, where applicable:

- Accession codes, unique identifiers, or web links for publicly available datasets
- A description of any restrictions on data availability
- For clinical datasets or third party data, please ensure that the statement adheres to our policy

The DNA methylation data generated in this study for longitudinal semaglutide and placebo samples,have been deposited in the GEO database under accession number: GSE327270. The clinical trial data are available under restricted access due to data privacy laws. All controlled-access clinical trial datasets provided by Dr. McComsey can be applied for by emailing grace.mccomsey@uhhospitals.org. Data use agreements and institutional IRB will be required. The processesd data are available in the Source data. Source data are provided with this paper.

## Research involving human participants, their data, or biological material

Policy information about studies with human participants or human data. See also policy information about sex, gender (identity/presentation), and sexual orientation and race, ethnicity and racism.

**Reporting on sex and gender**

Sex reported in Table 1. Sex-stratified subgroup analyses of epigenetic aging outcomes were not pre-specified and the study was not powered to detect sex-specific effects. Our model for each aging measure included the baseline value of that measure (to adjust for regression to the mean), treatment group, sex, baseline BMI, baseline HsCRP, and baseline sCD163.

**Reporting on race, ethnicity, or other socially relevant groupings**

Race or ethnicity reported in Table 1.

**Population characteristics**

For the epigenetic analysis, at baseline the 84 participants (45 semaglutide,39 placebo) were middle aged, with a mean±SD age of 49±12years and well balanced between treatment arms (48±13 vs. 50±12years). 42% were women overall, but men were slightly over represented in the semaglutide group (67% vs. 49%). The cohort included 58% Black, 38% White and 11% Hispanic participants with nearly identical distributions across groups. Immunologically, CD4 counts were high (median 762cells$\mu L^{-1}$) and CD4/CD8 ratios near 1.0, reflecting immune reconstitution; nadir CD4 counts were lower, as expected, but similar between arms. Viral suppression was durable: only 9% had HIV 1 RNA above the lower limit of quantification, and ART duration averaged ~14years. Participants were obese (median BMI 32.9$kgm^{-2}$) with comparable anthropometry in each group. One third were current smokers, another quarter former smokers. Glycemic control was normal (median HbA1c 5.5%), although insulin resistance was evident (median fasting HOMA IR 2.9), again without meaningful group differences. Estimated 10 year ASCVD risk was moderate at 4.7% (IQR2.2–8.0). Inflammatory biomarkers showed low grade activation: median high sensitivity CRP 4.1µg/mL and sCD163 605$pgmL^{-1}$, with slightly higher values in placebo. Overall, baseline characteristics of participants assayed in the epigenetic analysis were well matched.

**Recruitment**

To determine whether semaglutide treatment could impact biological aging, we conducted a post hoc exploratory epigenetic analysis of participants enrolled in a previously reported 32-week, randomized, double-blind, placebo-controlled phase 2b clinical trial evaluating semaglutide in people with HIV (PWH) and lipohypertrophy. A total of 154 individuals were screened, 108 were randomized (54 to each group). Eight participants (15%) in each arm discontinued prematurely, leaving 92 who completed 32 weeks; of these, 84 had paired samples available for epigenetic analysis. Randomization was 1:1 to semaglutide or placebo, stratified by sex, using block sizes of six via an online randomization system. Both participants and investigators were blinded to treatment assignment. All participants provided written informed consent.

**Ethics oversight**

The trial protocol was approved by the University Hospitals Cleveland Medical Center Institutional Review Board, and all participants provided written informed consent.

Note that full information on the approval of the study protocol must also be provided in the manuscript.

# Field-specific reporting

Please select the one below that is the best fit for your research. If you are not sure, read the appropriate sections before making your selection.

☒ Life sciences ☐ Behavioural & social sciences ☐ Ecological, evolutionary & environmental sciences

For a reference copy of the document with all sections, see nature.com/documents/nr-reporting-summary-flat.pdf

# Life sciences study design

All studies must disclose on these points even when the disclosure is negative.

| | |
|---|---|
| Sample size | A total of 154 individuals were screened, 108 were randomized (54 to each group). Eight participants (15%) in each arm discontinued prematurely, leaving 92 who completed 32 weeks; of these, 84 had paired samples available for epigenetic analysis. Randomization was 1:1 to semaglutide or placebo, stratified by sex, using block sizes of six via an online randomization system. |
| Data exclusions | 84 had paired samples available for epigenetic analysis. |
| Replication | Multiple epigenetic clocks were used for replication. As an orthogonal computational approach to assess biological aging with second-generation mortality-based epigenetic clocks, we used Biolearn, an open-source library for biomarkers of aging, to examine whether semaglutide treatment significantly impacted DNA methylation-based age estimates for GrimAge V1 and the updated GrimAge V2. |
| Randomization | Randomization was 1:1 to semaglutide or placebo, stratified by sex, using block sizes of six via an online randomization system. |
| Blinding | Both participants and investigators were blinded to treatment assignment. Blinding also occurred during epigenetic age assessments. |

# Reporting for specific materials, systems and methods

We require information from authors about some types of materials, experimental systems and methods used in many studies. Here, indicate whether each material, system or method listed is relevant to your study. If you are not sure if a list item applies to your research, read the appropriate section before selecting a response.

## Materials & experimental systems

| n/a | Involved in the study |
|---|---|
| ☒ | ☐ Antibodies |
| ☒ | ☐ Eukaryotic cell lines |
| ☒ | ☐ Palaeontology and archaeology |
| ☒ | ☐ Animals and other organisms |
| ☐ | ☒ Clinical data |
| ☒ | ☐ Dual use research of concern |
| ☒ | ☐ Plants |

## Methods

| n/a | Involved in the study |
|---|---|
| ☒ | ☐ ChIP-seq |
| ☒ | ☐ Flow cytometry |
| ☒ | ☐ MRI-based neuroimaging |

## Clinical data

Policy information about clinical studies

All manuscripts should comply with the ICMJE guidelines for publication of clinical research and a completed CONSORT checklist must be included with all submissions.

| | |
|---|---|
| Clinical trial registration | NCT04019197 |
| Study protocol | ClinicalTrials.gov NCT04019197 |
| Data collection | The Once-Weekly Semaglutide in People with HIV-Associated Lipohypertrophy study was a single-center, randomized, double-blind, placebo-controlled phase 2b trial (ClinicalTrials.gov NCT04019197) conducted at University Hospitals Cleveland Medical Center (Cleveland, OH). The primary trial objective was to evaluate semaglutide's effects on body fat distribution in PLWH over 32 weeks. |
| Outcomes | While the parent trial's primary outcomes were changes in adipose tissue volume (measured by CT) and body composition (DXA scans) at 32 weeks, our current analysis focuses on epigenetic aging markers as secondary/exploratory outcomes. |

## Plants

| | |
|---|---|
| Seed stocks | *Report on the source of all seed stocks or other plant material used. If applicable, state the seed stock centre and catalogue number. If plant specimens were collected from the field, describe the collection location, date and sampling procedures.* |
| Novel plant genotypes | *Describe the methods by which all novel plant genotypes were produced. This includes those generated by transgenic approaches, gene editing, chemical/radiation-based mutagenesis and hybridization. For transgenic lines, describe the transformation method, the number of independent lines analyzed and the generation upon which experiments were performed. For gene-edited lines, describe the editor used, the endogenous sequence targeted for editing, the targeting guide RNA sequence (if applicable) and how the editor was applied.* |
| Authentication | *Describe any authentication procedures for each seed stock used or novel genotype generated. Describe any experiments used to assess the effect of a mutation and, where applicable, how potential secondary effects (e.g. second site T-DNA insertions, mosiacism, off-target gene editing) were examined.* |
