# Supplementary material for: Semaglutide slows epigenetic aging in a randomized trial of HIV-associated lipohypertrophy
Source: Nat Commun. 2026 May 19;17:6606. doi: 10.1038/s41467-026-72861-3 (PMC13381876; doi:10.1038/s41467-026-72861-3)
Supplement: Supplementary file 3 — Transparent Peer Review file [file 41467_2026_72861_MOESM3_ESM.pdf]

nature portfolio

# Peer Review File

# Semaglutide Slows Epigenetic Aging in a Randomized Trial of HIV-Associated Lipohypertrophy

Corresponding Author: Dr Michael Corley

**This file contains all reviewer reports in order by version, followed by all author rebuttals in order by version.**

Version 0:

Reviewer comments:

Reviewer #1

(Remarks to the Author)
This is an important and novel study providing randomized clinical trial evidence that semaglutide can influence DNA methylation based biomarkers of aging in people with HIV-associated lipohypertrophy. The findings are timely, well conducted, and relevant for the emerging field of geroscience-informed therapeutics. The manuscript could be strengthened by additional discussion of contextual limitations and methodological considerations, as indicated below.

Comments
1. The analysis does not explicitly account for differences in antiretroviral treatment history, which could influence adiposity, metabolic outcomes, and epigenetic aging.
2. Epigenetic clocks may be sensitive to leukocyte composition, yet immune cell distribution and its potential impact on the results were not discussed.
3. The organ-specific epigenetic clocks provide intriguing evidence of multi-system benefit, but these measures are derived from peripheral blood and may not fully represent tissue-level aging.

Recommendations
4. Discuss the potential modifying role of ART history and recommend future analyses stratified by prior regimen exposures.
5. Acknowledge the absence of immune cell composition analysis and suggest incorporation of cell deconvolution or immune profiling in future studies.
6. Clarify that organ-system clocks are indirect and highlight the opportunity for confirmatory biopsy-based studies of adipose and muscle to validate tissue-level aging effects.

Reviewer #2

(Remarks to the Author)
This paper provided evidence using a randomized clinical trial that once-weekly semaglutide can modulate biological aging. Different approaches were applied to construct clocks across organs. This work is potentially important to better understand how semaglutide modulates epigenetic biomarkers of aging. I have two comments that may further improve this paper:

1. Although the epigenetic aging clock is a novel and interesting concept, there are several critiques regarding the validity and interpretability of analyses relying solely on these metrics. My main concern is what the deliverables of a statistically significant P-value actually imply—does it reflect shorter longevity, or is it clinically meaningful? Given these concerns, I recommend that the authors also evaluate other, more objective metrics that are recognized as biomarkers of aging to further validate their results. For example, in the context of brain aging, white matter microstructural integrity in the fornix could be a particularly informative marker. The following paper may be helpful.

Chen, C., Chen, S., Ye, Z., Shi, X., Ma, T., & Shardell, M. (2025). The Effect of Alcohol intake on Brain White Matter Microstructural Integrity: A New Causal Inference Framework for Incomplete Phenomic Data. Journal of the American Statistical Association, 1-18.

2. Given the small sample size, I wonder whether the predicted clocks perform as expected. Validation of these outcomes is

essential to ensure the validity of downstream analyses—for example, by evaluating their association with chronological age and assessing prediction accuracy in an independent validation dataset.

Reviewer #3

(Remarks to the Author)
The authors used blood samples from a completed clinical trial with the GLP-1 receptor agonist semaglutide. This drug was given to HIV patients with HIV-associated lipohypertrophy and obesity. DNA was isolated from peripheral blood mononuclear cells (PBMCs) at the beginning of the trial and 32 weeks after treatment. DNA methylation was analyzed using Illumina arrays and the methylation was characterized across multiple published epigenetic aging clocks. Semaglutide significantly decreased epigenetic aging with eleven clocks tested whereas two other clocks showed no such trend. This data suggests a remarkable effect of semaglutide on epigenetic clocks. The analysis of multiple available clocks in a randomized placebo-controlled trial is a strength of this manuscript. While these observations are very interesting, we still completely lack a mechanistic understanding of how these clocks work and whether the clock-like changes do in fact contribute to aging.
I have several specific concerns and questions:

1) The authors used peripheral blood mononuclear cell samples collected at baseline and at week 32 of treatment. How can they exclude the possibility that there was a change in PBMC composition over the treatment period, perhaps different between controls and treatment groups? Altered blood cell composition would translate into DNA methylation differences that are erroneously scored with the different DNA methylation clocks.

2) How can epigenetic signatures be “detrimental,” as the manuscript states. This gets us to the point where one must consider whether clock-like epigenetic methylation changes are causative to aging or not. I think they are not. Instead, they likely reflect stochastic changes at CpG sites that are most susceptible to change over time or under several conditions that tend to induce a change.
The authors should read and cite PMID 40968181.

3) It is mysterious how DNA methylation–based “system clocks” derived from a blood cell methylation assay can predict specific organ decline with aging. The authors should try to come up with an explanation for this phenomenon, otherwise this all remains unexplained observation. The relevant work cited in this context (reference 31) has not been peer-reviewed.

4) The authors used the 850k Methylation EpicV2 bead chips from Illumina. These assays tend to score very small differences in DNA methylation. In many cases such small differences may have questionable biological meaning. Can the authors indicate what kind of differences, in terms of beta values or percent differences, they are measuring when comparing the placebo and semaglutide samples.

5) The authors should make an attempt to propose a mechanism how semaglutide would change methylation at clock-associated CpG sites.

6) Supplementary Figure 1 should be a main Figure.

7) Supplementary Figure 3:
I don’t understand what the authors are trying to say with this diagram. Most squares are empty.

8) In the Methods section the authors state that “the models first extract a subset of CpGs relevant to DNAm surrogates for plasma proteins and smoking pack-years..”
Plasse explain what that means.

Version 1:

Reviewer comments:

Reviewer #1

(Remarks to the Author)
I recommend accepting the revised manuscript. The authors have carefully addressed my comments and concerns. This manuscript is anticipated to significantly impact the field of HIV and aging, in the context of GLP-1 receptor agonists.

Reviewer #2

(Remarks to the Author)
The authors have addressed my comments

Reviewer #3

(Remarks to the Author)
The authors have addressed all my concerns. I recommend publication.

**Open Access** This Peer Review File is licensed under a Creative Commons Attribution 4.0 International License, which permits use, sharing, adaptation, distribution and reproduction in any medium or format, as long as you give appropriate credit to the original author(s) and the source, provide a link to the Creative Commons license, and indicate if changes were made.
In cases where reviewers are anonymous, credit should be given to 'Anonymous Referee' and the source.
The images or other third party material in this Peer Review File are included in the article's Creative Commons license, unless indicated otherwise in a credit line to the material. If material is not included in the article's Creative Commons license and your intended use is not permitted by statutory regulation or exceeds the permitted use, you will need to obtain permission directly from the copyright holder.
To view a copy of this license, visit https://creativecommons.org/licenses/by/4.0/

**Reviewers' Comments to Author:**

**Reviewer: 1**

**Comments to the Author**

This is an important and novel study providing randomized clinical trial evidence that semaglutide can influence DNA methylation based biomarkers of aging in people with HIV-associated lipohypertrophy. The findings are timely, well conducted, and relevant for the emerging field of geroscience-informed therapeutics. The manuscript could be strengthened by additional discussion of contextual limitations and methodological considerations, as indicated below.

Reviewer #1 Comment 1: *The analysis does not explicitly account for differences in antiretroviral treatment history, which could influence adiposity, metabolic outcomes, and epigenetic aging.*

***Response:*** **We appreciate the reviewer's important point regarding the potential influence of antiretroviral treatment (ART) history on adiposity, metabolic outcomes, and epigenetic aging. While ART regimen and treatment history were not included as covariates in the present epigenetic analyses, we previously examined this issue in post hoc analyses of the parent randomized clinical trial (PMID: 38964353). In that analysis, we observed no differences at baseline or in unadjusted changes over 32 weeks in weight or BMI among participants randomized to semaglutide when stratified by current use of integrase strand transfer inhibitors (INSTIs) and tenofovir alafenamide, or by prior exposure to thymidine analogue nucleoside reverse transcriptase inhibitors (PMID: 38964353). These findings suggest that differential ART exposure was unlikely to have materially influenced the primary adiposity-related outcomes of the trial. Nonetheless, we acknowledge that ART history may have independent effects on epigenetic aging biomarkers, and we have added text to the Discussion noting this as a limitation and an important area for future investigation in larger studies specifically powered to examine ART-specific epigenetic effects.**

Reviewer #1 Comment 2: *Epigenetic clocks may be sensitive to leukocyte composition, yet immune cell distribution and its potential impact on the results were not discussed.*

***Response:***

**We agree that epigenetic clock measures can be influenced by underlying leukocyte composition. To address this, we performed additional analysis and leveraged an enhanced DNA methylation–based reference deconvolution methods that use highly distinct, cell-type–specific methylation signatures to estimate immune cell proportions (PMID: 35140201). Specifically, we deconvoluted proportions of immune cell subtypes (monocytes, naïve and memory B cells, naïve and memory CD4 + and CD8 + T cells, natural killer, and T regulatory cells) in PBMC samples at both baseline and week 32 in participants randomized to placebo or semaglutide. These estimates were derived using validated reference libraries developed with the IDOL (IDentifying Optimal Libraries) algorithm, which enables robust discrimination among leukocyte subtypes using DNA methylation microarray data. This approach has been extensively validated against artificial mixtures and whole-blood samples with known cellular composition by flow cytometry. We did not observe systematic differences in immune cell proportions between treatment groups over the study period, suggesting that changes in epigenetic aging biomarkers were unlikely to be driven by shifts in leukocyte composition at this level of resolution. We have included this data as new supplementary figure 2 and also expanded the Discussion to acknowledge the sensitivity of epigenetic clocks to immune cell distribution and to clarify limitations to account for this in our analyses.**

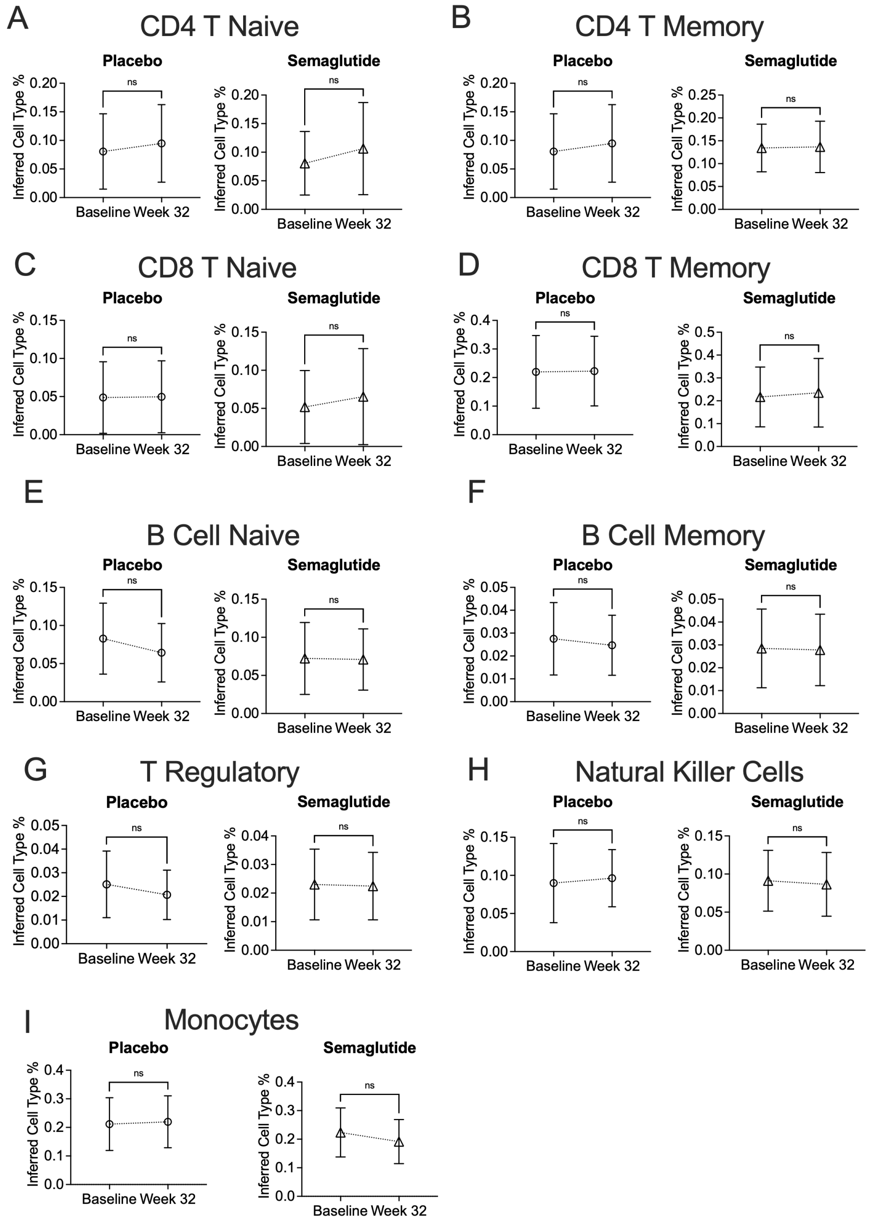


***Supplemental Figure 2: Immune cell-type DNA methylation deconvolution analysis shows no significant differences between treatment groups over time. Estimated proportions of major peripheral immune cell populations were derived using cell-type deconvolution and compared between placebo and semaglutide groups at baseline and Week 32. Panels show (A) CD4 T naïve, (B) CD4 T memory, (C) CD8 T naïve, (D) CD8 T memory, (E) B cell naïve, (F) B cell memory, (G) regulatory T cells, (H) natural killer cells, and (I) monocytes. Data are presented as mean ± SEM. Within-group longitudinal comparisons (baseline vs. Week 32) revealed no statistically significant changes for any immune cell subtype (ns), indicating that semaglutide treatment did not alter immune cell composition relative to placebo over the study period.***

**Importantly, several of the epigenetic clocks included in our analyses were explicitly designed to be minimally influenced by leukocyte composition. In particular, DunedinPACE and related second-generation clocks emphasize longitudinal pace-of-aging signals and incorporate features that reduce sensitivity to variation in blood cell-type distributions, as demonstrated in prior validation studies. Consistent effects across clocks with differing sensitivity to immune cell composition further support that the observed treatment-related differences are unlikely to be driven primarily by changes in leukocyte proportions. Nonetheless, we acknowledge that residual confounding by immune cell composition cannot be fully excluded, particularly in PBMC-based analyses.**

*<u>Reviewer #1 Comment 3:</u> The organ-specific epigenetic clocks provide intriguing evidence of multi-system benefit, but these measures are derived from peripheral blood and may not fully represent tissue-level aging.*

***<u>Response:</u>*** **We agree that these organ-specific epigenetic clocks are derived from peripheral blood and therefore do not directly measure tissue-resident epigenetic aging. However, as now demonstrated in the recently published *Nature Aging* study describing these organ-specific epigenetic clocks, they were explicitly trained to reflect system-level aging processes as captured by circulating protein biomarkers that have been widely used to quantify systems-specific biological aging and functional decline. We have revised the Discussion to clarify that these systems clocks should be interpreted as blood-based integrative biomarkers of organ-system aging, grounded in and validated against established proteomic measures, while acknowledging that future studies incorporating direct tissue methylation or longitudinal clinical outcomes will be important to further validate tissue-specific effects of semaglutide.**

**The parent randomized controlled trial comprehensively assessed organ-specific aging-related features through quantitative imaging and metabolic profiling. Semaglutide treatment over 32 weeks led to substantial reductions in total body fat, including marked loss of abdominal visceral adipose tissue (approximately 30.6%), which is strongly linked to cardiometabolic risk and HIV-associated lipohypertrophy. Improvements were also seen in liver density, indicative of reduced hepatic fat content, while ectopic fat in muscle was unchanged. Key metabolic parameters reflecting organ function, such as glucose tolerance, insulin resistance (HOMA-IR), blood pressure, and lipid profiles (notably increased HDL cholesterol and decreased triglycerides and VLDL cholesterol), improved significantly with semaglutide. Lean body mass and muscle volume showed modest decreases that were not statistically significant. Importantly, these clinically meaningful changes in fat distribution and metabolic health provide physiological context supporting the observed multi-system epigenetic age deceleration, as reductions in visceral and ectopic fat and improved metabolic parameters represent key facets of organ aging and dysfunction. The trial also demonstrated that these benefits occurred independently of baseline factors such as BMI, HIV treatment regimen, or sex. Nonetheless, epigenetic age analyses of cell types from biopsies or accessible organs should be pursued in future studies.**

*<u>Reviewer #1 Comment 4:</u> Discuss the potential modifying role of ART history and recommend future analyses stratified by prior regimen exposures.*

***<u>Response:</u>*** **In the parent study, exploratory analyses evaluated whether baseline or longitudinal outcomes differed according to antiretroviral therapy (ART) history, including current use of tenofovir alafenamide (TAF) or integrase strand transfer inhibitors (INSTIs), and prior exposure to thymidine analogue nucleoside reverse transcriptase inhibitors (tNRTIs). No statistically significant differences were observed at baseline or in changes in weight, body composition, or metabolic parameters over 32 weeks between participants stratified by these ART exposures. Similarly, in the semaglutide group, baseline BMI, HbA1c, CD4 T-cell count, current TAF or INSTI use, and prior NRTI exposure were not predictive of achieving ≥5% reductions in total body weight, fat mass, or visceral adipose tissue. These findings suggest that the observed multi-system effects of semaglutide, including reductions in epigenetic age and improvements in organ-related measures, are likely independent of ART history.**
**Nonetheless, given the known impact of specific ART regimens on fat distribution, liver function, and metabolic health in people with HIV, future studies with larger sample sizes should pre-specify stratified analyses by prior and current ART exposure. Such analyses could further clarify whether specific ART histories modify semaglutide's effects on epigenetic aging, organ-specific outcomes, or metabolic improvements, thereby enhancing personalized treatment strategies in people with HIV. We thank the reviewer for the comment of ART history and have revised our discussion to highlight this important area of investigation.**

_Reviewer #1 Comment 5:_ *Acknowledge the absence of immune cell composition analysis and suggest incorporation of cell deconvolution or immune profiling in future studies.*

***Response:*** **We have expanded our discussion to address this and have included cell deconvolution analyses. To address this, we incorporated DNA methylation–based deconvolution methods in our analyses to estimate proportions of 12 leukocyte subtypes (including naïve and memory CD4+ and CD8+ T cells, T regulatory cells, B cells, natural killer cells, monocytes, and granulocytes). These estimates were derived using well-validated reference libraries and the IDOL algorithm, which has been shown to accurately capture immune-cell profiles in PBMCs using DNA methylation data. While these analyses provide a robust, high-resolution view of baseline immune composition and allow us to account for potential confounding effects on epigenetic clocks, we recognize that future studies would benefit from direct flow cytometry or single-cell profiling to more precisely define immune cell dynamics in response to semaglutide.**

_Reviewer #1 Comment 6:_ *Clarify that organ-system clocks are indirect and highlight the opportunity for confirmatory biopsy-based studies of adipose and muscle to validate tissue-level aging effects.*

***Response:*** **We agree with the reviewer that organ-system epigenetic clocks derived from peripheral blood are indirect measures of tissue-specific aging. These clocks capture systemic aging signals that reflect, in part, the impact of metabolic, inflammatory, and immune processes across multiple organs but do not directly measure aging within individual tissues themselves. While blood-based clocks provide a valuable and clinically accessible approach to monitoring multi-system aging, they are not a direct proxy for tissue-specific epigenetic changes.**

**To further validate the tissue-level aging effects observed in our study, we recognize the importance of future research incorporating biopsy-based studies of adipose and muscle tissue. Direct tissue sampling would provide a more accurate representation of organ-specific aging processes, allowing for a more precise comparison with the systemic effects captured by blood-based clocks. Such studies would be essential to better understand the tissue-specific mechanisms driving the changes in fat, muscle, and metabolic health observed with semaglutide treatment. We have included this suggestion in the Discussion to highlight the potential for confirmatory studies in larger cohorts with tissue samples, which would further enhance our understanding of the molecular basis underlying semaglutide's multi-system effects.**

**Reviewer: 2**

**Comments to the Author**

This paper provided evidence using a randomized clinical trial that once-weekly semaglutide can modulate biological aging. Different approaches were applied to construct clocks across organs. This work is potentially important to better understand how semaglutide modulates epigenetic biomarkers of aging. I have two comments that may further improve this paper:

_Reviewer #2 Comment 1:_ *Although the epigenetic aging clock is a novel and interesting concept, there are several critiques regarding the validity and interpretability of analyses relying solely on these metrics. My main concern is what the deliverables of a statistically significant P-value actually imply—does it reflect shorter longevity, or is it clinically meaningful? Given these concerns, I recommend that the authors also evaluate other, more objective metrics that are recognized as biomarkers of aging to further validate their results. For example, in the context of brain aging, white matter microstructural integrity in the fornix could be a particularly informative marker. The following paper may be helpful.*

*Chen, C., Chen, S., Ye, Z., Shi, X., Ma, T., & Shardell, M. (2025). The Effect of Alcohol intake on Brain White Matter Microstructural Integrity: A New Causal Inference Framework for Incomplete Phenomic Data. Journal of the American Statistical Association, 1-18.*

***Response:*** **To address the reviewer's concern about the clinical meaning of epigenetic aging metrics, we have expanded our discussion. We agree and also plan to incorporate additional, well-established biomarkers of aging in future studies to better contextualize these findings. Specifically, biomarkers such as white matter microstructural integrity, including measurements in regions like the fornix, offer a promising alternative for assessing brain aging. We thank the reviewer for highlighting the recent study by (Chen et al., 2025). We agree that these objective, imaging measures of brain aging could serve as a meaningful complement to the molecular changes captured by epigenetic clocks and help to validate our findings on aging. We would like to acknowledge recent work by our group examining the impact of semaglutide on cognitive measures (PMID: 41098140). After adjusting for sex and absolute CD4 count, only the visuospatial score remained statistically significant (p=0.05). Semaglutide's total natural direct effect (TNDE) maintained a positive effect on the visuospatial score while accounting for potential changes in high sensitivity C-reactive protein and soluble CD163 levels (p=0.04).This aligns with a hypothesis that semaglutide may have broader neuroprotective effects, potentially through its modulation of inflammatory pathways, which are implicated in cognitive decline in this population.**

*Reviewer #2 Comment 2: Given the small sample size, I wonder whether the predicted clocks perform as expected. Validation of these outcomes is essential to ensure the validity of downstream analyses—for example, by evaluating their association with chronological age and assessing prediction accuracy in an independent validation dataset.*

***Response:*** **We appreciate the reviewer's important concern regarding the small sample size and its potential impact on the performance of the predicted epigenetic clocks. We agree that validation is critical to ensure the robustness and accuracy of these clocks, especially when using them for downstream analyses. Importantly, the clocks used in our analyses have been extensively validated in large, independent cohorts. As demonstrated recently in *Mavrommatis et al.* Nature Communications(PMC12708718), epigenetic clocks were first evaluated for their expected strong associations with chronological age, confirming accurate age prediction performance, which is a foundational requirement for their validity. Beyond chronological age, the study further assessed clock performance in an independent cohort of approximately 18,859 individuals, examining prediction accuracy and longitudinal associations with incident disease and mortality outcomes over up to 10 years of follow-up. This large-scale external validation showed that several clocks, particularly second- and third-generation clocks, exhibited robust and biologically meaningful associations with health outcomes, supporting their reliability and appropriateness for downstream analyses despite smaller sample sizes in individual studies.**

**We have performed additional analyses for our study and examined all epigenetic clocks utilized and their associations with chronological age, a well-established method for testing the accuracy of epigenetic clocks. Our analysis demonstrated that the predicted clocks performed as expected, with significant correlations between predicted epigenetic age and chronological age, which supports the validity of the clocks within this sample. This result is consistent with previous studies validating similar epigenetic clocks, providing confidence in their use for our primary analyses. We have included these results as a new supplementary figure 1.**

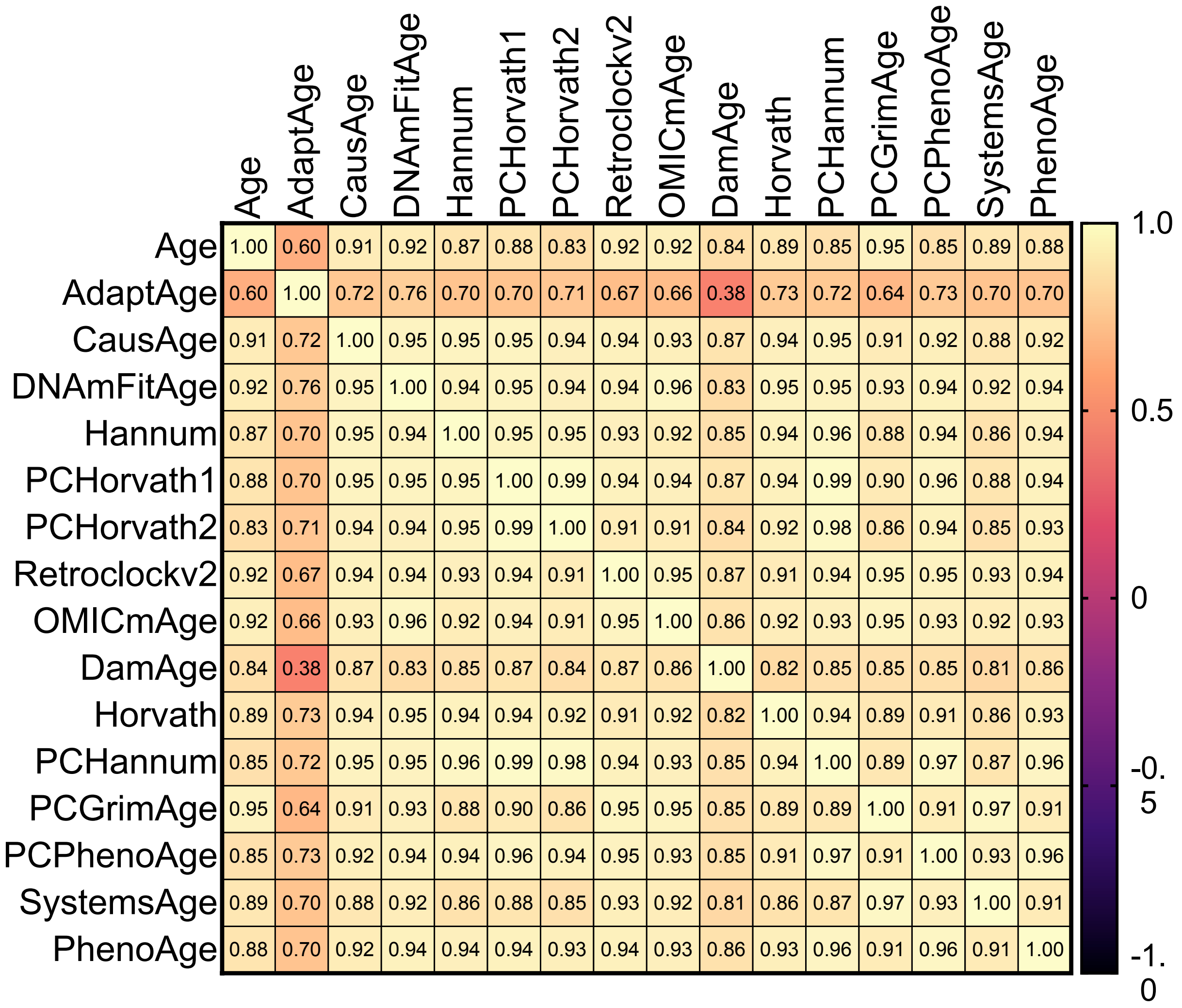


| | Age | AdaptAge | CausAge | DNAmFitAge | Hannum | PCHorvath1 | PCHorvath2 | Retroclockv2 | OMICmAge | DamAge | Horvath | PCHannum | PCGrimAge | PCPhenoAge | SystemsAge | PhenoAge |
|---|---|---|---|---|---|---|---|---|---|---|---|---|---|---|---|---|
| Age | 1.00 | 0.60 | 0.91 | 0.92 | 0.87 | 0.88 | 0.83 | 0.92 | 0.92 | 0.84 | 0.89 | 0.85 | 0.95 | 0.85 | 0.89 | 0.88 |
| AdaptAge | 0.60 | 1.00 | 0.72 | 0.76 | 0.70 | 0.70 | 0.71 | 0.67 | 0.66 | 0.38 | 0.73 | 0.72 | 0.64 | 0.73 | 0.70 | 0.70 |
| CausAge | 0.91 | 0.72 | 1.00 | 0.95 | 0.95 | 0.95 | 0.94 | 0.94 | 0.93 | 0.87 | 0.94 | 0.95 | 0.91 | 0.92 | 0.88 | 0.92 |
| DNAmFitAge | 0.92 | 0.76 | 0.95 | 1.00 | 0.94 | 0.95 | 0.94 | 0.94 | 0.96 | 0.83 | 0.95 | 0.95 | 0.93 | 0.94 | 0.92 | 0.94 |
| Hannum | 0.87 | 0.70 | 0.95 | 0.94 | 1.00 | 0.95 | 0.95 | 0.93 | 0.92 | 0.85 | 0.94 | 0.96 | 0.88 | 0.94 | 0.86 | 0.94 |
| PCHorvath1 | 0.88 | 0.70 | 0.95 | 0.95 | 0.95 | 1.00 | 0.99 | 0.94 | 0.94 | 0.87 | 0.94 | 0.99 | 0.90 | 0.96 | 0.88 | 0.94 |
| PCHorvath2 | 0.83 | 0.71 | 0.94 | 0.94 | 0.95 | 0.99 | 1.00 | 0.91 | 0.91 | 0.84 | 0.92 | 0.98 | 0.86 | 0.94 | 0.85 | 0.93 |
| Retroclockv2 | 0.92 | 0.67 | 0.94 | 0.94 | 0.93 | 0.94 | 0.91 | 1.00 | 0.95 | 0.87 | 0.91 | 0.94 | 0.95 | 0.95 | 0.93 | 0.94 |
| OMICmAge | 0.92 | 0.66 | 0.93 | 0.96 | 0.92 | 0.94 | 0.91 | 0.95 | 1.00 | 0.86 | 0.92 | 0.93 | 0.95 | 0.93 | 0.92 | 0.93 |
| DamAge | 0.84 | 0.38 | 0.87 | 0.83 | 0.85 | 0.87 | 0.84 | 0.87 | 0.86 | 1.00 | 0.82 | 0.85 | 0.85 | 0.85 | 0.81 | 0.86 |
| Horvath | 0.89 | 0.73 | 0.94 | 0.95 | 0.94 | 0.94 | 0.92 | 0.91 | 0.92 | 0.82 | 1.00 | 0.94 | 0.89 | 0.91 | 0.86 | 0.93 |
| PCHannum | 0.85 | 0.72 | 0.95 | 0.95 | 0.96 | 0.99 | 0.98 | 0.94 | 0.93 | 0.85 | 0.94 | 1.00 | 0.89 | 0.97 | 0.87 | 0.96 |
| PCGrimAge | 0.95 | 0.64 | 0.91 | 0.93 | 0.88 | 0.90 | 0.86 | 0.95 | 0.95 | 0.85 | 0.89 | 0.89 | 1.00 | 0.91 | 0.97 | 0.91 |
| PCPhenoAge | 0.85 | 0.73 | 0.92 | 0.94 | 0.94 | 0.96 | 0.94 | 0.95 | 0.93 | 0.85 | 0.91 | 0.97 | 0.91 | 1.00 | 0.93 | 0.96 |
| SystemsAge | 0.89 | 0.70 | 0.88 | 0.92 | 0.86 | 0.88 | 0.85 | 0.93 | 0.92 | 0.81 | 0.86 | 0.87 | 0.97 | 0.93 | 1.00 | 0.91 |
| PhenoAge | 0.88 | 0.70 | 0.92 | 0.94 | 0.94 | 0.94 | 0.93 | 0.94 | 0.93 | 0.86 | 0.93 | 0.96 | 0.91 | 0.96 | 0.91 | 1.00 |

**Supplemental Figure 1: Correlation matrix of participants observed chronological age across multiple epigenetic clocks. Heatmap showing pairwise Pearson correlation coefficients (r) between predicted age (from epigenetic clock / DNA methylation-based model) and actual chronological age. Values represent correlation coefficients (r), ranging from 0.38 (lowest, highlighted in deep red) to 1.00 (perfect correlation).**

**Reviewer: 3**

**Comments to the Author**

The authors used blood samples from a completed clinical trial with the GLP-1 receptor agonist semaglutide. This drug was given to HIV patients with HIV-associated lipohypertrophy and obesity. DNA was isolated from peripheral blood mononuclear cells (PBMCs) at the beginning of the trial and 32 weeks after treatment. DNA methylation was analyzed using Illumina arrays and the methylation was characterized across multiple published epigenetic aging clocks. Semaglutide significantly decreased epigenetic aging with eleven clocks tested whereas two other clocks showed no such trend. This data suggests a remarkable effect of semaglutide on epigenetic clocks. The analysis of multiple available clocks in a randomized placebo-controlled trial is a strength of this manuscript. While these observations are very interesting, we still completely lack a mechanistic understanding of how these clocks work and whether the clock-like changes do in fact contribute to aging.
I have several specific concerns and questions:

*<u>Reviewer #3 Comment 1:</u> The authors used peripheral blood mononuclear cell samples collected at baseline and at week 32 of treatment. How can they exclude the possibility that there was a change in PBMC composition over the treatment period, perhaps different between controls and treatment groups? Altered blood cell composition would translate into DNA methylation differences that are erroneously scored with the different DNA methylation clocks.*

***<u>Response:</u>*** **We appreciate the reviewer's thoughtful comment regarding the possibility that changes in peripheral blood mononuclear cell (PBMC) composition could influence the DNA methylation profiles observed over the treatment period, especially between the treatment and control groups. We recognize that alterations in immune cell composition could potentially confound the interpretation of DNA methylation clocks, as changes in cell types can lead to differential methylation patterns that might be misinterpreted as biological aging effects.**

**To address this concern, we employed DNA methylation–based deconvolution techniques to estimate immune cell proportions in PBMC samples at both baseline and week 32 of treatment. This approach allows us to account for any changes in immune cell composition by estimating the relative proportions of different immune cell types, such as CD4+ and CD8+ T cells, B cells, monocytes, and other leukocyte subtypes. These proportions were then incorporated into our analysis to adjust for any potential confounding effects of immune cell distribution on DNA methylation changes.**

**Furthermore, in post hoc analyses of the parent study, we observed no significant differences in immune cell composition between the treatment and placebo groups over the 32-week period, suggesting that the treatment did not induce substantial shifts in PBMC composition that would likely influence DNA methylation markers. This was consistent with the absence of significant differences in weight loss, BMI, or metabolic parameters across subgroups stratified by ART history (e.g., INSTI or tenofovir alafenamide use).**

**Despite this, we acknowledge that immune cell distribution could still have a subtle impact on the results, and we have highlighted this limitation in the Discussion. We suggest that future studies could employ more detailed flow cytometry or single-cell RNA sequencing to further confirm immune cell composition over time, ensuring a more precise measurement of its potential impact on DNA methylation changes.**

*<u>Reviewer #3 Comment 2:</u> How can epigenetic signatures be "detrimental," as the manuscript states. This gets us to the point where one must consider whether clock-like epigenetic methylation changes are causative to aging or not. I think they are not. Instead, they likely reflect stochastic changes at CpG sites that are most susceptible to change over time or under several conditions that tend to induce a change.*
*The authors should read and cite PMID 40968181.*

***<u>Response:</u>*** **We thank the reviewer for this important conceptual point. We agree that describing epigenetic signatures as "detrimental" could be misleading, as clock-like CpG methylation changes are unlikely to be causal drivers of aging and instead largely reflect stochastic variation and systemic influences over time. In response, we have removed the term "detrimental" from the manuscript and revised the Discussion to emphasize that accelerated epigenetic age is a biomarker associated with adverse health outcomes, rather than a direct mechanistic cause. We have also added the suggested reference (PMID 40968181) to support the interpretation that age-associated methylation changes arise from stochastic processes at CpG sites susceptible to change under multiple conditions. These edits clarify that epigenetic clocks serve as integrative readouts of biological aging while acknowledging that the underlying CpG changes are predominantly non-causal.**

*<u>Reviewer #3 Comment 3:</u> It is mysterious how DNA methylation–based "system clocks" derived from a blood cell methylation assay can predict specific organ decline with aging. The authors should try to come up with an explanation for this phenomenon, otherwise this all remains unexplained observation. The relevant work cited in this context (reference 31) has not been peer-reviewed.*

***Response:*** **We note that the previously cited work (reference 31) has now been published, and we have updated the manuscript accordingly. In addition, we have expanded the Discussion to better articulate this conceptual framework and explicitly added limitations acknowledging that the precise causal mechanisms linking blood methylation changes to organ-specific aging remain incompletely understood and warrant further mechanistic investigation.**

*Reviewer #3 Comment 4: The authors used the 850k Methylation EpicV2 bead chips from Illumina. These assays tend to score very small differences in DNA methylation. In many cases such small differences may have questionable biological meaning. Can the authors indicate what kind of differences, in terms of beta values or percent differences, they are measuring when comparing the placebo and semaglutide samples.*

***Response:*** **We agree that individual CpG-level differences detected by the Illumina Infinium MethylationEPIC v2 array are often small in magnitude, and that isolated CpG changes may have limited biological interpretability. In our study, the observed differences between placebo and semaglutide groups at individual clock-associated CpG sites were generally modest, typically on the order of ~10% absolute differences in beta values ($\Delta\beta \approx 0.10$).**

**Importantly, our primary analyses do not rely on interpreting biological meaning at individual CpG sites. Instead, we focus on epigenetic clock outputs (e.g., GrimAge and related measures), which are composite biomarkers derived from weighted combinations of hundreds to thousands of CpGs. Prior work has demonstrated that small, coordinated shifts across many CpG sites can translate into meaningful changes in epigenetic age estimates and are predictive of morbidity and mortality, even when individual CpG differences are subtle.**

**Furthermore, the magnitude of methylation differences observed in our study is comparable to those reported in prior intervention and longitudinal aging studies using the EPIC platform, including studies of lifestyle modification, pharmacologic treatment, and weight loss, where modest CpG-level changes nevertheless resulted in detectable shifts in epigenetic age acceleration.**

**We have revised the Methods and Discussion to clarify the typical beta-value differences observed and to emphasize that the biological interpretation pertains to aggregated clock-based measures rather than single-CpG effects.**

*Reviewer #3 Comment 5: The authors should make an attempt to propose a mechanism how semaglutide would change methylation at clock-associated CpG sites.*

***Response:*** **We agree that the precise molecular mechanisms by which semaglutide may influence DNA methylation at clock-associated CpG sites are not yet fully established. However, several biologically plausible pathways can be proposed.**

**Semaglutide is a glucagon-like peptide-1 receptor agonist (GLP-1RA) that improves glycemic control, promotes weight loss, and reduces systemic inflammation and cardiometabolic risk. These downstream physiological effects are known modulators of epigenetic regulation. In particular, chronic hyperglycemia, insulin resistance, obesity, and inflammation have all been associated with accelerated epigenetic aging and altered methylation at clock-associated CpGs. By improving insulin sensitivity, reducing adiposity, and lowering inflammatory burden, semaglutide may indirectly shift the cellular environment toward a more metabolically favorable and less pro-inflammatory state, leading to remodeling of DNA methylation patterns captured by epigenetic clocks.**

**In addition, GLP-1 signaling has been shown to influence mitochondrial function, oxidative stress, and nutrient-sensing pathways (e.g., AMPK, mTOR), which are closely linked to aging biology and epigenetic maintenance. Alterations in these pathways can affect the activity of DNA methyltransferases and demethylases, providing a mechanistic link between semaglutide exposure and changes in methylation at clock-relevant CpG sites.**

**Importantly, epigenetic clocks such as GrimAge and related measures are composite biomarkers that integrate methylation changes associated with smoking exposure, inflammation, metabolic dysregulation, and circulating protein levels. Therefore, changes in clock estimates following semaglutide treatment likely reflect downstream consequences of improved cardiometabolic and inflammatory profiles rather than direct drug effects on individual CpG sites.**
**We have added text to the Discussion to clarify that the observed methylation changes are most plausibly mediated by semaglutide's systemic metabolic and inflammatory effects, while acknowledging that causal molecular mechanisms remain to be elucidated in future experimental studies.**

*Reviewer #3 Comment 6: Supplementary Figure 1 should be a main Figure.*

***Response:*** **We agree and have included this figure as new main Figure 2.**

*Reviewer #3 Comment 7: Supplementary Figure 3:*
*I don't understand what the authors are trying to say with this diagram. Most squares are empty.*

***Response:*** **We have removed this supplemental figure for clarity.**

*Reviewer #3 Comment 8: In the Methods section the authors state that "the models first extract a subset of CpGs relevant to DNAm surrogates for plasma proteins and smoking pack-years.."*
*Plasse explain what that means.*

***Response:*** **This procedure follows the original methodology used to construct the GrimAge epigenetic clock, and here we applied the same framework to our dataset.**
**Specifically, GrimAge was originally developed by first identifying subsets of CpG sites whose DNA methylation (DNAm) levels serve as surrogate predictors for smoking pack-years and for circulating plasma protein levels. Smoking pack-years quantify cumulative cigarette exposure and are calculated as the number of packs smoked per day multiplied by the number of years smoked. In the Framingham Heart Study (FHS), smoking pack-years were computed using smoking data. For plasma proteins, GrimAge leveraged immunoassay-based measurements of circulating biomarkers including cardiovascular disease–related markers such as C-reactive protein and growth differentiation factor 15.**

**Each target variable (smoking pack-years or an individual plasma protein) was regressed on genome-wide CpG methylation levels in the FHS training dataset using elastic net regression (implemented via the glmnet R package). Ten-fold cross-validation was used to select the regularization parameter (λ). This penalized regression approach simultaneously performs prediction and CpG selection, yielding a subset of CpGs with non-zero coefficients that together form DNAm-based surrogate estimators.**

**In summary, the statement that the models "extract a subset of CpGs relevant to DNAm surrogates for plasma proteins and smoking pack-years" refers to this GrimAge-derived, penalized regression framework, which we applied unchanged to our dataset.**
